# Complementary, alternative, and integrative medicine therapies for knee osteoarthritis: a visual analytics analysis of the research domain

**DOI:** 10.3389/fmed.2025.1586357

**Published:** 2025-06-11

**Authors:** Chuqing Luo, Jing Wang, Jiaojiao Guo

**Affiliations:** ^1^Health Management Center, General Practice Medical Center, West China Hospital, Sichuan University, Chengdu, Sichuan, China; ^2^West China School of Nursing, Sichuan University, Chengdu, Sichuan, China

**Keywords:** knee osteoarthritis, aging, complementary therapies, integrative medicine, pain management, musculoskeletal health

## Abstract

**Introduction:**

Knee osteoarthritis (KOA) affects 528 million people globally, with prevalence projected to rise by 74.9% by 2050. Traditional treatments face limitations in long-term safety and efficacy, driving interest in complementary, alternative, and integrative medicine therapies (CAIMT) for symptom relief and disease management. This article present an analysis of this research domain.

**Methods:**

A systematic search of Web of Science (1994–2024) identified 1,389 publications using CAIMT-related keywords. Bibliometric tools analyzed publication volume, citations, authorship, institutional collaborations, geographical distribution, keyword co-occurrence, and thematic evolution. Metrics assessed academic impact, and Bradford's Law identified core journals.

**Results:**

Publications surged post-2010, peaking in 2022 (123 papers). China (345 publications) and the USA (282 publications) dominated output. International co-authorship comprised 22.46%. Bennell K. L. (*n* = 39), Hinman R. S. (*n* = 29), and Henriksen M. (*n* = 13) were top authors. The University of Melbourne led institutional contributions. Keyword analysis revealed eight clusters: such as Pain management, exercise/rehabilitation, Nutritional interventions, Emerging therapies. Thematic Evolution: Focus shifted from singular therapies pre-2010 to integrative strategies post-2020, emphasizing personalized and evidence-based approaches.

**Conclusion:**

CAIMT offers promising adjunctive strategies for KOA management, particularly for aging populations seeking non-pharmacological options. Future research should prioritize personalized, integrative approaches and rigorous evidence generation to refine clinical guidelines.

## 1 Introduction

Knee osteoarthritis (KOA) is a common degenerative joint disease that affects approximately 528 million people worldwide, with a prevalence rate as high as 23% among those aged 40 and older (1). With the aging population and increasing life expectancy, the incidence of KOA is expected to rise by 74.9% by 2050 (2). Traditional treatment methods, such as non-steroidal anti-inflammatory drugs (NSAIDs), analgesics, and intra-articular corticosteroid injections, can effectively relieve symptoms in the short term. However, due to the potential adverse effects associated with long-term use and their inherent limitations in halting disease progression (3), an increasing number of patients are seeking complementary and alternative medicine (CAM) as alternative or adjunct treatment options (4, 5). It is noteworthy that while “complementary” and “alternative” are often combined conceptually, they represent distinct clinical approaches: complementary medicine refers to non-mainstream practices used alongside conventional treatments, whereas alternative medicine denotes approaches substituting conventional therapy (6).

Contemporary CAM modalities for KOA primarily encompass acupuncture, herbal medicine, manual therapies, and mind-body practices. These interventions aim to alleviate pain, improve function, and potentially modify disease progression through multimodal mechanisms (7–10). The emergence of integrative medicine has further emphasized the systematic combination of evidence-based conventional treatments with selected complementary therapies, creating personalized treatment paradigms (11–13).^1^ This evolution reflects growing patient demand for non-pharmacological, individualized care approaches while maintaining scientific rigor. Recent years have witnessed exponential growth in research examining complementary, alternative, and integrative medicine therapies (CAIMT) for KOA, necessitating bibliometric analysis to map scientific output, identify research trends, and clarify the evolving therapeutic landscape. Such analysis is crucial for optimizing clinical decision-making and advancing patient-centered care models.

We employed bibliometric analysis and narrative review methods to review the research on CAIMT for KOA from the database's inception 1994 to 2024. Through bibliometric analysis, we assessed not only the volume and growth trends of the relevant literature but also utilized visualization tools to reveal research themes, author collaboration networks, and the distribution of research institutions. Moreover, we also conducted an in-depth analysis of the current application status of common CAIMT in KOA to uncover the actual effects and potential applications of these therapies in clinical practice. To our knowledge, this study represents the first comprehensive analysis in the field to integrate bibliometric methods with a narrative review framework, providing a multidimensional perspective on CAIMT for KOA. By combining quantitative mapping of research trends with critical review, this approach enhances the depth and scope of understanding in this evolving domain. Furthermore, we utilized the comprehensive standardized search string developed by Ng et al. (14), which includes 604 keywords or phrases related to CAIMT. Additionally, we also employed scientific metrics encompassing the h-index, g-index, and m-index, instead of citation counts, to assess the contributions and impact of the publications. This methodology has not been applied in previous research. Through this comprehensive analysis, we aim to provide solutions for academia and clinical practice to address the challenges of an aging society, enabling them to better enhance the integrated management of elderly patients with KOA, advance the application of innovative treatment strategies, and promote the development of holistic medical approaches, ultimately improving the quality of life in this population.

## 2 Methods

### 2.1 Data sources and retrieval strategy

We conducted a bibliometric analysis using the Web of Science Core Collection (WOS-CC) database, selected for its multidisciplinary coverage of high-impact journals, robust citation tracking capabilities, and compatibility with bibliometric software tools (e.g., VOSviewer and Bibliometrix). While databases like PubMed and EMBASE focus on clinical literature, WOS-CC's broader disciplinary scope aligns with our objective to map the full spectrum of complementary, alternative, and integrative medicine therapies (CAIMT) research, which spans biomedical, social, and humanities domains. The limitations of this single-database approach are addressed in Section 4.1.

The search strategy followed the BIBLIO guidelines for reporting bibliometric reviews (15). We adapted the search string developed by Ng et al. (14), which systematically identified CAIMT-related terms across seven databases (MEDLINE, EMBASE, AMED, PsycINFO, CINAHL, Scopus, and WOS). The full search syntax, combining CAIMT terms with knee osteoarthritis (KOA) subject headings using Boolean operators, is provided in Supplementary material. To ensure comprehensive coverage of the research field's evolution, no time restrictions were applied, and all records from the database's inception to the search date (December 21, 2024) were included. The search was restricted to English-language “articles” and “review articles” to maintain consistency in bibliometric analysis.

### 2.2 Literature screening

Inclusion criteria: only articles that mentioned one or more CAIMT approaches for the potential prevention, treatment, or management of KOA in older adults were included in this study. Eligible study designs comprised randomized clinical trials, semi-randomized clinical trials, observational studies, case series, case reports, systematic reviews, and meta-analyses. Exclusion criteria: Articles that focused on interventions other than CAIMT, did not involve older adults with KOA, were animal studies, duplicated publications, non-English publications, or lacked systematic and in-depth analysis (e.g., Editorial Material, Letters, Meeting Abstracts, Book Reviews, Data Papers, Notes, Retractions, Early Access articles, Corrections, Proceedings Papers, Retracted Publications, News Items, etc.) were excluded. We specify that two researchers (Luo C.Q. and Wang J.) independently screened titles and abstracts, resolving discrepancies through discussion or consultation with a third researcher (Guo J.J.). This ensures reproducibility and minimizes bias. All articles from the search results were exported to EndNote (Version 21), where duplicates were identified and removed.

### 2.3 Analytical tools and methods

We conducted the descriptive analysis of data and visualization work with VOSviewer (Version1.6.19) and Bibliometrix (within RStudio, Version2024.12.0+467), which are well-suited for processing and analyzing large-scale literature datasets (16). Before the analysis, we first conducted a systematic preprocessing and cleaning of the metadata, which included merging synonyms and removing meaningless terms, to ensure accuracy and reliability. Subsequently, we employed the aforementioned visualization tools to perform descriptive analysis of data on the publication volume and its impact, while examining journal distribution, author affiliations, geographical distribution, and collaboration networks. Additionally, we carried out keyword co-occurrence clustering and trend theme evolution analyses to uncover the prevailing topics and their evolutionary paths in the research field. To present the research results more intuitively and effectively, we also utilized scientific visualization tools such as Pajek (Vervion 64 5.19) (17), SCImago Graphica (Vervion Beta 1.0.46) (18), and OriginPro 2025 (Learning Edition) for data visualization (19). In addition, we conducted a review of the current status of the use of common CAIMT in KOA to assess their effectiveness and safety and provide guidance for clinical practice.

## 3 Results

### 3.1 Main information and publication trends

**Figure 1:**
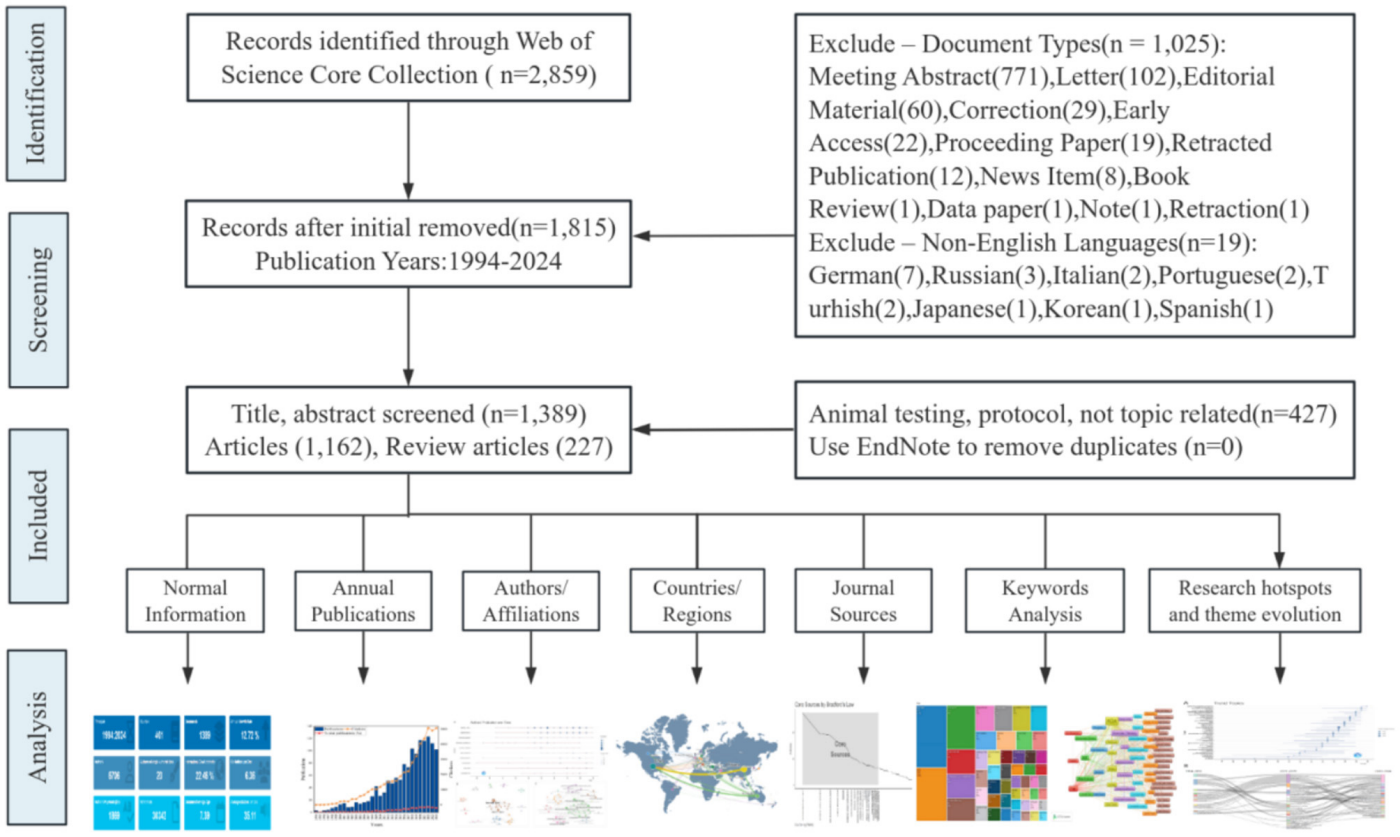
Screening process and quantitative analysis steps of publications. This diagram outlines the systematic process of identifying, screening, and including records from the Web of Science Core Collection for a research review, detailing the number of records at each stage and the criteria for exclusion based on document type, language, and relevance. The analysis phase highlights various aspects analyzed from the selected articles.

**Figure 2:**
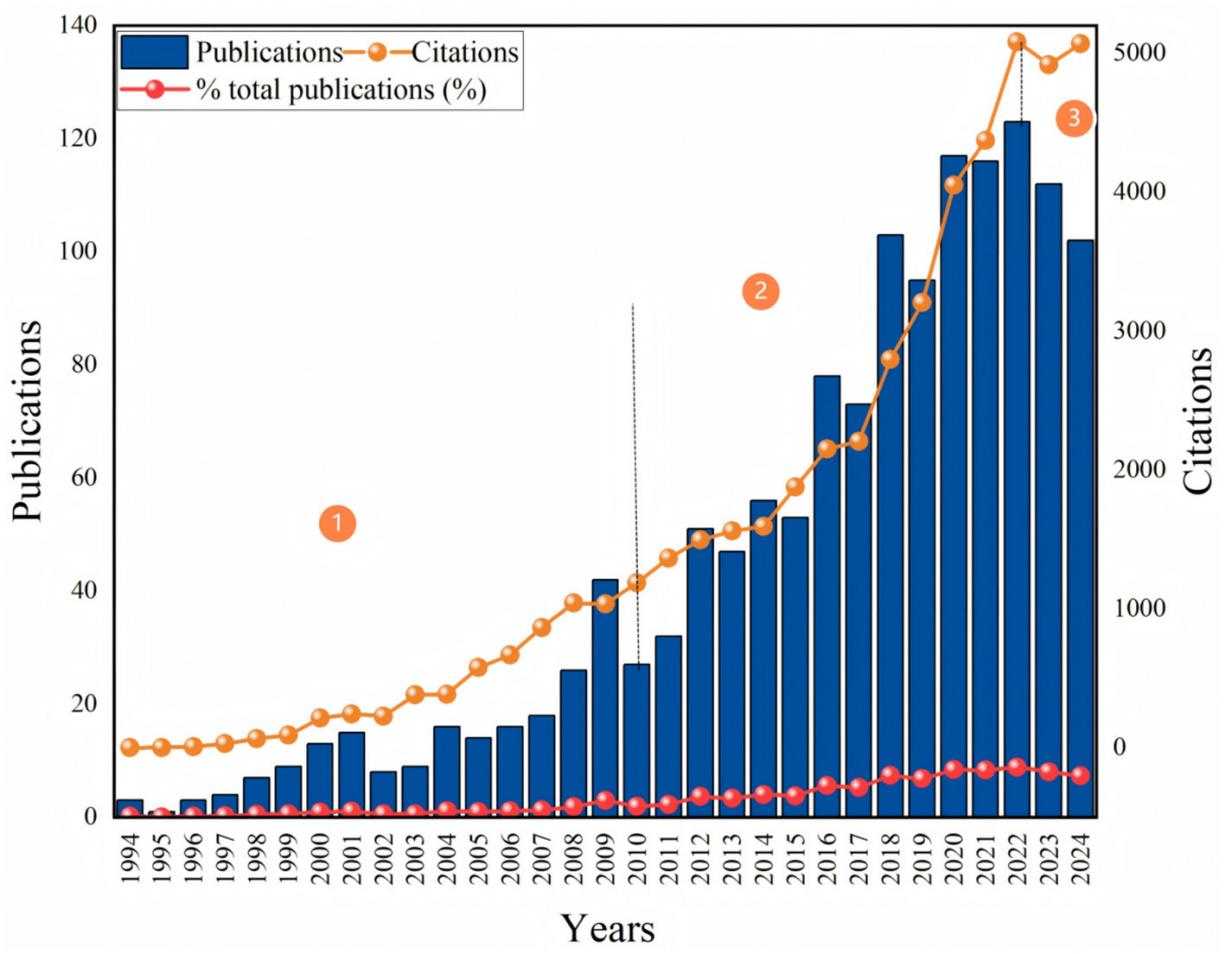
Trends in the number of publications vs. citations between 1994 and 2024. Blue bars indicate the number of publications per year, orange line graphs indicate the number of citations for the corresponding year, and red lines show the percentage of total publications. Markers 1, 2, and 3 point out key moments, respectively, indicating significant increases in the number of publications and citations and their correlation.

Figure 1 illustrates the screening process of publications and the bibliometric analysis method. Initially, 2,859 publications were retrieved. After screening and excluding 1,452 records that did not meet the criteria, we ultimately identified 1,389 papers (1,162 articles and 227 review articles) authored by 6,706 authors and published in 401 different journals. Figure 2 shows the publication and citation data from 1994 to 2024. We observed that both publications and citations have demonstrated consistent growth over time, particularly starting around 2010, peaking in 2022 with 123 publications, which accounted for 8.8% of all publications, indicating an increasing body of work and recognition in the field. Despite a slight decline in the number of publications over the past 2 years, this upward trend is expected to continue in the foreseeable future, given the increasing attention of global scholars toward the burden of osteoarthritis (20, 21). The rise in citation counts, especially the sharp increase in recent years, reflects the impact and relevance of the published research. We summarized the general findings of the publications in Table 1.

**Table 1:** General information included in the publication.

**Description**	**Results**	**Description**	**Results**
Timespan	1994:2024	Authors	6,706
Documents	1,389	Authors of single-authored docs	20
Article	1,162	Single-authored docs	21
Review	227	Co-authors per doc	6.36
Annual growth rate %	12.72	International co-authorships %	22.46
Document average age	7.39	Affiliations	1,347
Average citations per doc	35.11	Countries/regions	67
References	30,342	Sources (Journals, Books, etc.)	401
Keywords plus	1,954	Open access types	6
Author's keywords (after merging)	1,427	Funding agencies	100

### 3.2 Authors and affiliations

**Figure 3:**
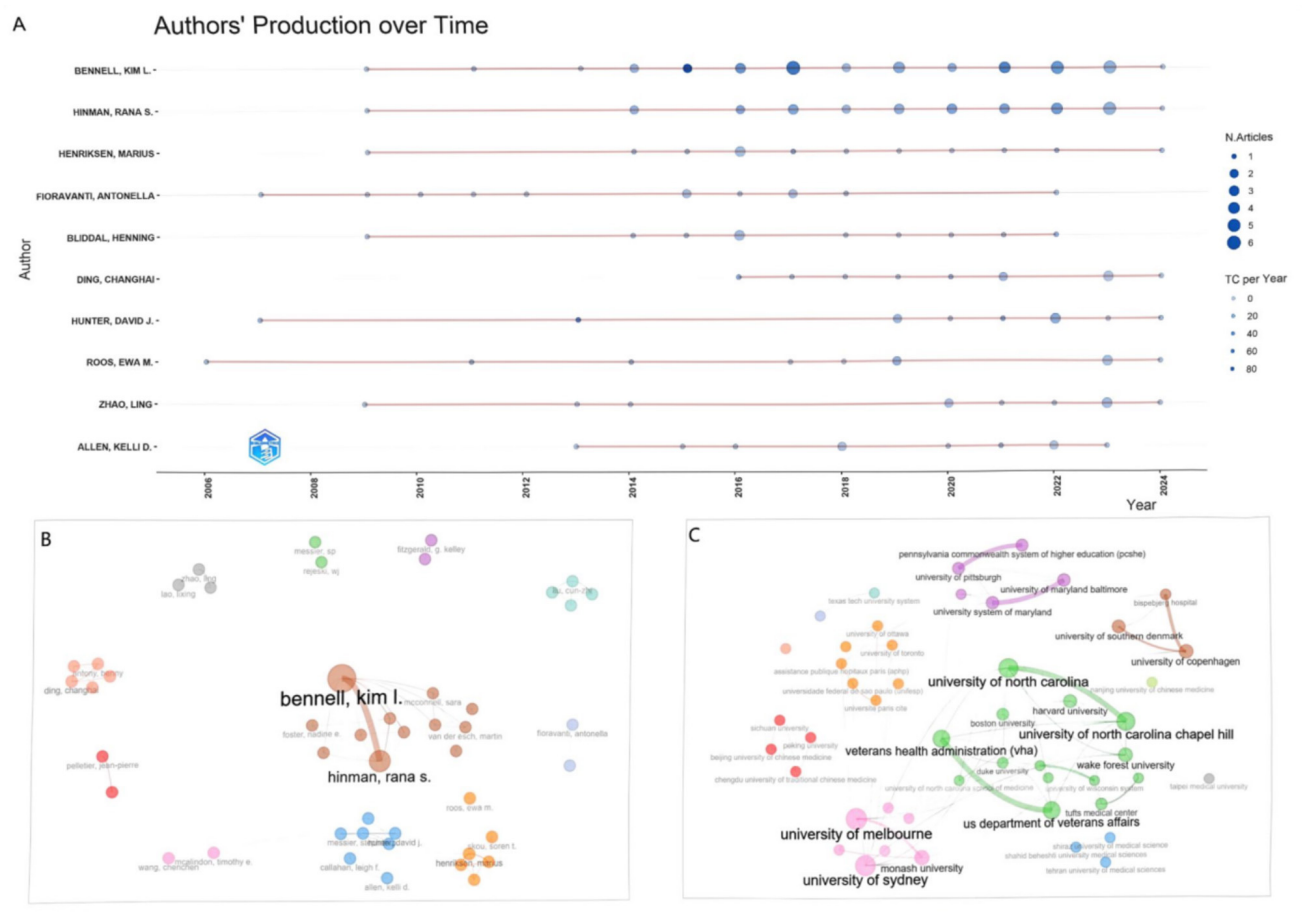
Visualization of authors' production and collaboration networks. **(A)** illustrates the publication trends over time for selected authors, highlighting the number of articles (N. Articles) and total citations (TC per Year). **(B, C)** depict collaborative networks, showcasing relationships among authors **(B)** and institutional connections **(C)** based on their publication history.

The top three authors in terms of publication volume are Bennell K.L. (*n* = 39), Hinman R.S. (*n* = 29), and Henriksen M. (*n* = 13). They have remained consistently active since 2009, continuing to the present (Figure 3A). Based on the Price's Law formula $(N=0.749\sqrt{n_{\max}}$, *n*_max_ is the number of papers with the highest productive authors) (22), we identified 85 core authors (approximately 1.27% of all authors) within this field, who collectively contributed the majority of the outputs, with an average publication count of 8.19 (ranging from 6 to 39). Unlike previous studies, this research integrated the h-index, g-index, and m-index to conduct a comprehensive assessment of the academic impact of authors in the field of KOA, to reveal their true contributions (23). Table 2 lists the top ten most influential and contributing authors. Only 20 authors published research findings independently, while strong collaborative relationships were formed among other authors, leading to the establishment of multiple research teams (Figure 3B). The research interests of these authors span a wide range, including exercise therapy, physical therapy, nutrition, and so on. Meanwhile, 55.6% of affiliated institutions published no fewer than 2 research outputs. The top three institutions in terms of publication output were *the University of Melbourne* (71 publications), *Wake Forest University* (67 publications), and *the University of North Carolina* (66 publications). The co-occurrence and collaboration network of institutions (Figure 3C) revealed that research institutions in this field exhibited diversified characteristics. These institutions encompass comprehensive universities, medical schools, traditional Chinese medicine universities, hospital systems, research institutions, and more, and they maintain close collaborative relationships. They played pivotal roles in research on CAIMT for KOA, collectively driving advancements in this field.

**Table 2:** The top ten most influential authors.

**Rank**	**Authors**	**h_index**	**g_index**	**m_index**	**Citations**	**Publications**
1	Bennell, Kim L.	24	39	1.5	2,672	39
2	Hinman, Rana S.	18	29	1.125	1,133	29
3	Fioravanti, Antonella	11	12	0.611	498	12
4	van der Esch, Martin	10	10	1	1,298	10
5	Bliddal, Henning	9	11	0.563	333	11
6	Henriksen, Marius	9	13	0.563	476	13
7	David Rabago	9	10	0.692	389	10
8	Bartholdy, Cecilie	8	10	0.727	432	10
9	Dekker, Joost	8	9	0.727	269	9
10	Fitzgerald, G. Kelley	8	8	0.5	302	8

### 3.3 Countries/regions distribution and funding agencies

**Figure 4:**
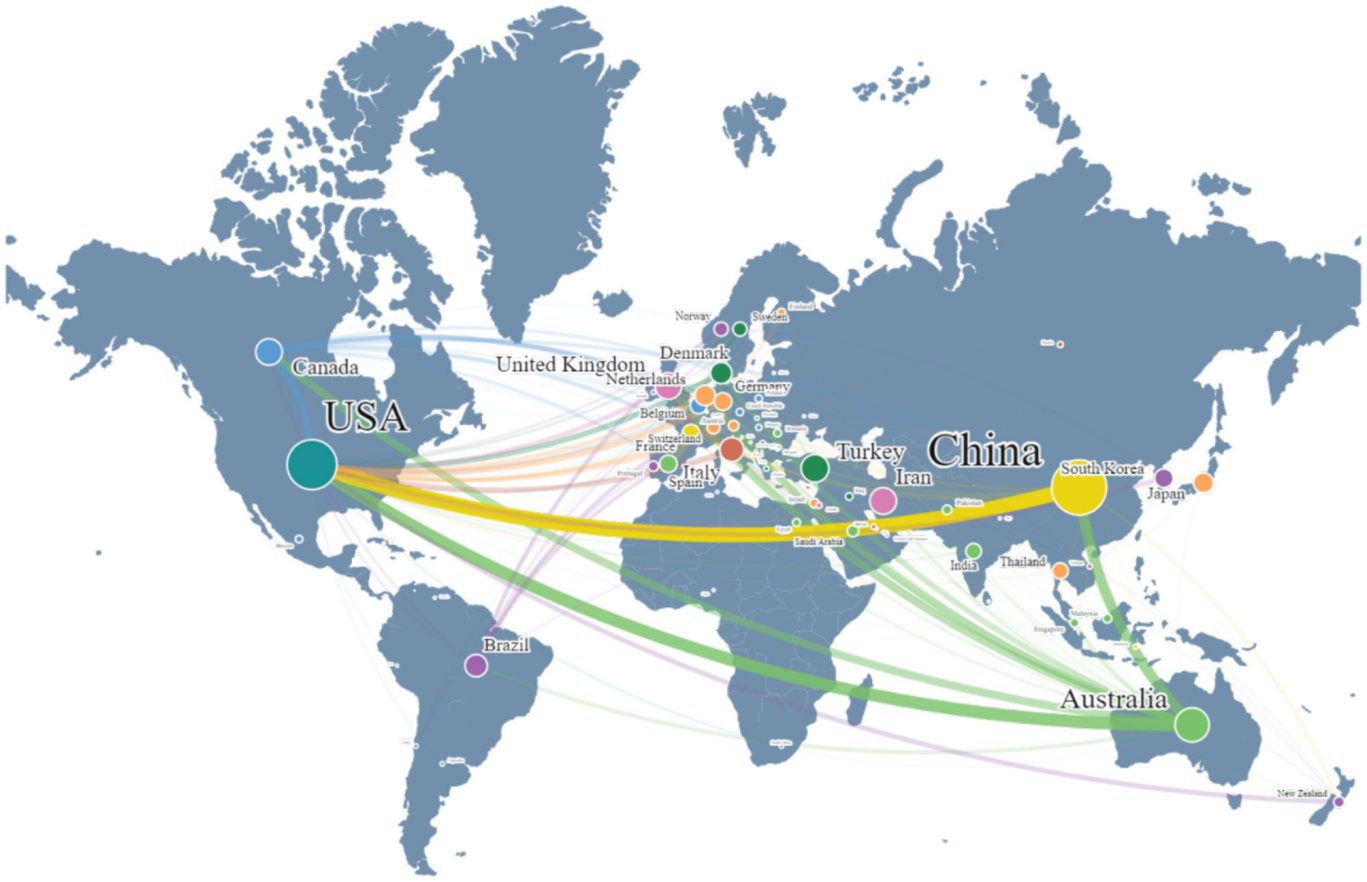
Global publication quantity and collaboration relationship distribution map. The size of the spheres and the font represent the number of publications in each country; the more publications, the larger the sphere and font. Different colors of lines indicate the collaborative relationships between countries, with thicker lines representing closer collaborations.

Sixty seven countries/regions worldwide have contributed to publications in this field. The top 10 countries in the number of publications were China (*n* = 345), the United States of America (*n* = 282), Australia (*n* = 133), Turkey (*n* = 87), the United Kingdom (*n* = 82), Iran (*n* = 80), Canada (*n* = 79), Italy (*n* = 66), Brazil (*n* = 56), and Denmark (*n* = 52). Figure 4 illustrates the distribution of their publications. Through the visualization of publication quantities by country, the size of the spheres represents the volume of publications, with larger spheres indicating a higher number of publications; similarly, the font size reflects this trend. Furthermore, the connecting lines reveal collaboration relationships between countries, with thicker lines indicating closer cooperation. It was evident that China and the United States led significantly in publication numbers, with particularly large spheres representing their dominance in the global publishing landscape on related topics. The distribution of connecting lines indicated that, in addition to the close cooperation between China and the U.S., other countries such as Australia, Canada, Brazil, and several European nations also demonstrated strong collaborative networks, reflecting active academic interactions within the region. Further analysis reveals that international co-authorship accounts for 22.46%. These results underscore the importance of international academic collaboration and its facilitative role in research outcomes. To further understand the funding support in this research field, we conducted a descriptive analysis of data of the funding agencies. We found that 995 publications received funding from 100 different institutions. Among them, the U.S. Department of Health and Human Services (*n* = 115) and the National Institutes of Health (NIH) (*n* = 111) from the United States, as well as the National Natural Science Foundation of China (NSFC) (*n* = 74), are the institutions with the highest number of funded publications, together accounting for 30.2% of the funding support. Additionally, several organizations and government funding agencies from Australia, Canada, Japan, and Denmark have also contributed to the development of this research field.

### 3.4 Core sources of publications

**Figure 5:**
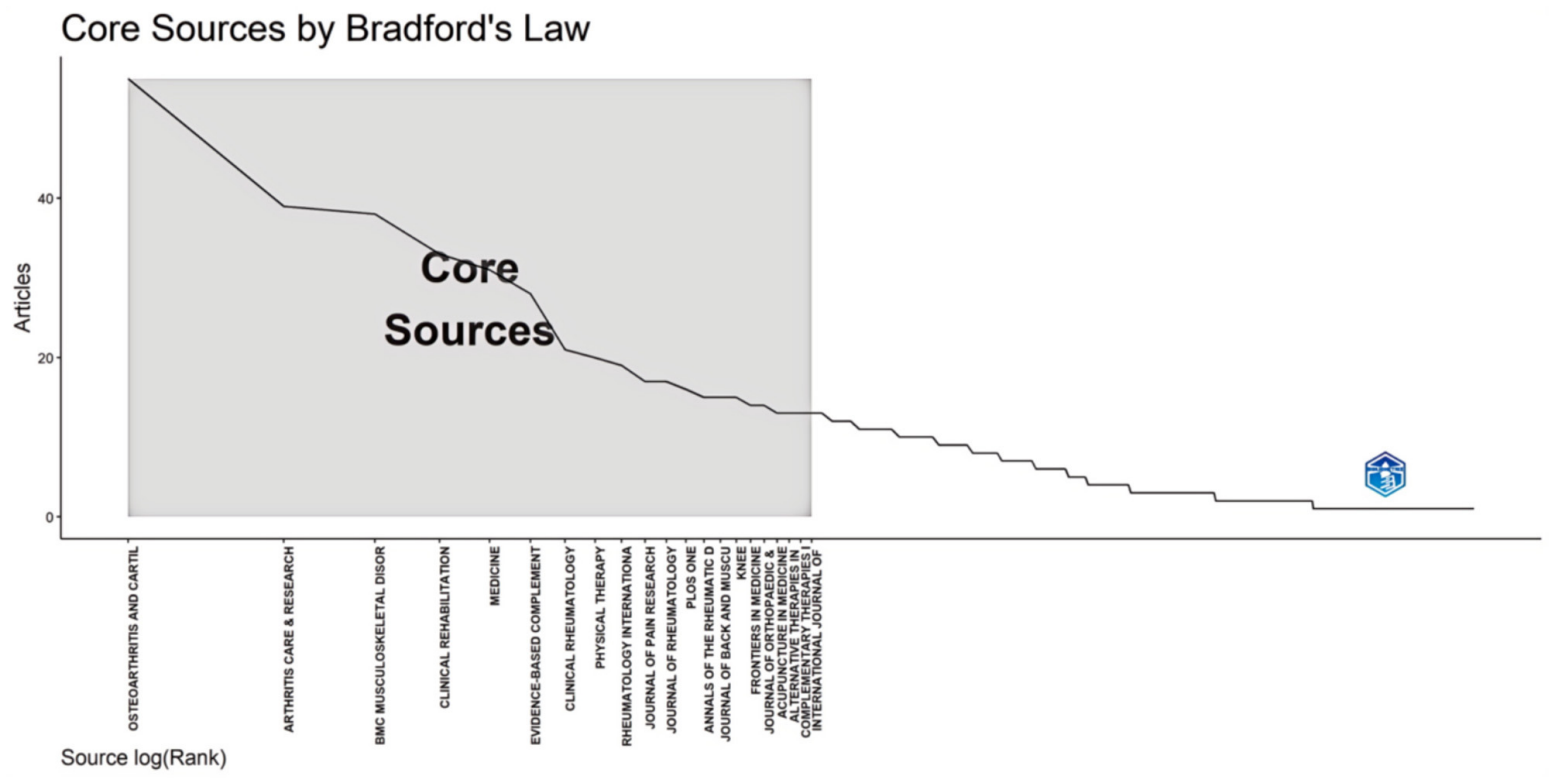
Core Sources (Journals, Books, etc.) by Bradford's Law. The X-axis represents the ranking of literature sources (scaled logarithmically), while the Y-axis indicates the number of relevant publications. The highlighted “core sources” region in the figure shows that the number of articles published in these sources is significantly higher than in others, reflecting the importance of a few key journals in contributing to the literature within a specific research field.

Bradford's Law is often employed to reveal the distribution patterns of literature across source journals, indicating that a small number of core journals account for the majority of high-quality literature in this field (24). Figure 5 illustrates the main sources of literature analyzed according to Bradford's Law. Detailed information regarding these core journals was provided in Table 3. Twenty one core journals published a total of 459 articles on CAIMT for KOA in older adults from 1994 to 2024, accounting for 33.05% of all publications. In addition, we observed that *Osteoarthritis and Cartilage* leads with 55 publications (accounting for 3.96% of the total), with a 5-year impact factor of 7 and a citation index of 2.21, both falling within the Q1 range. *Arthritis Care & Research* and *BMC Musculoskeletal Disorders* follow closely, with 39 publications (2.81%) and 38 publications (2.74%), respectively; their impact factors are 4.3 and 2.6, categorizing them as Q1 and Q2 journals. Additionally, several Q1 journals such as *Clinical Rehabilitation* and *Physical Therapy* also demonstrate strong publication numbers and influence. The five-year impact factors for these journals range from 1.6 to 18.4, indicating a significant quantity and quality in academic publications related to osteoarthritis and musculoskeletal diseases. Overall, the academic influence and collaboration potential of these journals in relevant fields deserve attention and showcase diverse research directions and clinical applications.

**Table 3:** The number of publications of core source journals and their impact factors and citation indicators.

**Sources**	**Publications (%)**	**IF**	**CI**	**CQ**
*Osteoarthritis and Cartilage*	55 (3.96%)	7	2.21	Q1
*Arthritis Care and Research*	39 (2.81%)	4.3	1.44	Q1
*BMC Musculoskeletal Disorders?*	38 (2.74%)	2.6	0.82	Q2
*Clinical Rehabilitation*	33 (2.38%)	3.4	1.09	Q1
*Medicine*	31 (2.23%)	1.6	0.35	Q2
*Evidence-based Complementary and Alternative Medicine*	28 (2.02%)	3.014	0.75	Q3
*Clinical Rheumatology*	21 (1.51%)	2.8	0.77	Q2
*Physical Therapy*	20 (1.44%)	3.9	1.62	Q1
*Rheumatology International*	19 (1.37%)	2.9	0.87	Q2
*Journal of Pain Research*	17 (1.22%)	2.8	0.68	Q2
*Journal of Rheumatology*	17 (1.22%)	3.9	1.02	Q2
*PLoS ONE*	16 (1.16%)	3.3	0.88	Q1
*Annals of the Rheumatic Diseases*	15 (1.08%)	18.4	5.1	Q1
*Journal Of Back And Musculoskeletal Rehabilitation*	15 (1.08%)	1.5	0.76	Q3
*Knee*	15 (1.08%)	2	0.79	Q2
*Frontiers in Medicine*	14 (1.01%)	3.4	0.84	Q1
*Journal Of Orthopaedic and Sports Physical Therapy*	14 (1.01%)	6.3	2.06	Q1
*Acupuncture In Medicine*	13 (0.94%)	2.4	0.61	Q2
*Alternative Therapies in Health and Medicine*	13 (0.94%)	1.7	0.48	Q3
*Complementary Therapies in Clinical Practice*	13 (0.94%)	2.8	0.81	Q2
*International Journal of RheumaticDiseases*	13 (0.94%)	2.5	0.62	Q2

IF, Impact Factor (5 Year); CI, Citation Indicator (2023); CQ, Category Quartile of JCR.

### 3.5 High frequency keywords

**Figure 6:**
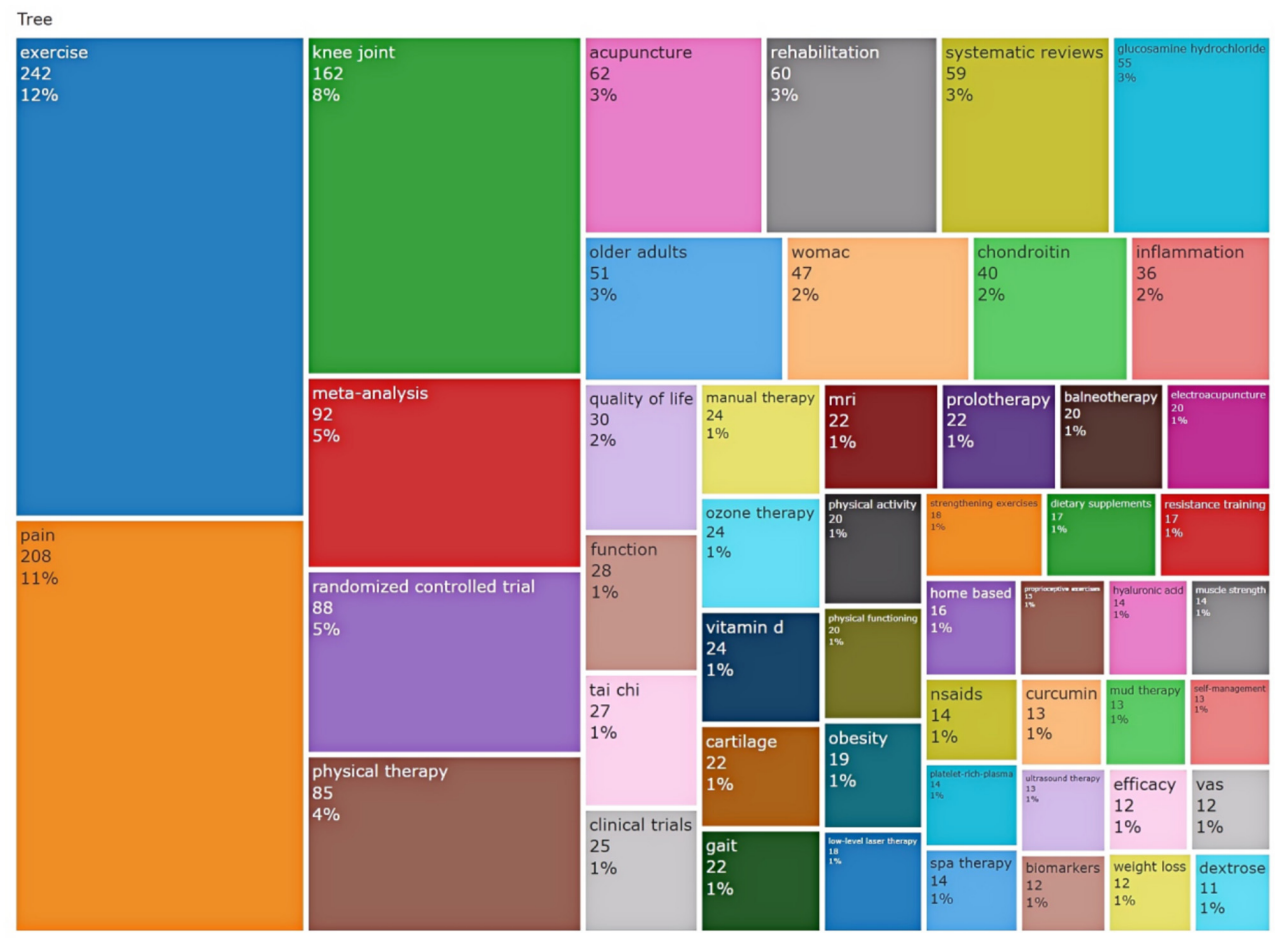
Treemap of research topics and their distribution. This treemap visualizes the distribution of key research topics in the dataset, highlighting the number of publications associated with each topic and their relative proportions. The largest categories include “exercise,” “pain,” and “knee joint,” indicating areas of significant research focus.

To identify the popular topics in the field of osteoarthritis research related to CAIMT, we generated a TreeMap (Figure 6) by using Bibliometrix, highlighting the top 50 frequent keywords (excluding KOA). The map visualizes the number of publications on various themes through differently sized blocks and color coding. The results showed that the keywords “exercise,” “pain,” and “knee joint” dominated (31%), indicating the significant role of exercise therapy in alleviating pain and improving joint function in patients with KOA. We found that among the top 10 keywords ranked by frequency, the most popular complementary therapies include: physical therapy (*n* = 85), acupuncture (*n* = 62), rehabilitation (*n* = 60), glucosamine hydrochloride (*n* = 55), chondroitin (*n* = 40), tai chi (*n* = 27), manual therapy (*n* = 24), ozone therapy (*n* = 24), vitamin D (*n* = 24), and prolotherapy (*n* = 22). These keywords highlighting the diversity and complexity of current treatment approaches for KOA. Additionally, the TreeMap illustrated emerging topics such as “platelet-rich plasma” and “biomarkers,” which warrant further exploration. Furthermore, Themes related to older adults, such as “elderly” and “function,” as well as studies focusing on quality of life, constitute a notable proportion, suggesting a significant impact of KOA on this population. Notably, the frequent occurrence of research designs such as “meta-analysis (5%),” “randomized controlled trial (5%),” and “systematic review (3%)” reflected an increasing emphasis on evidence-based medical approaches. Overall, the distribution of these keywords not only reveals the hotspots in KOA research but also provides important references for future research directions, encouraging researchers to continue exploring more effective treatment strategies.

### 3.6 Keywords co-occurrence clustering

**Figure 7:**
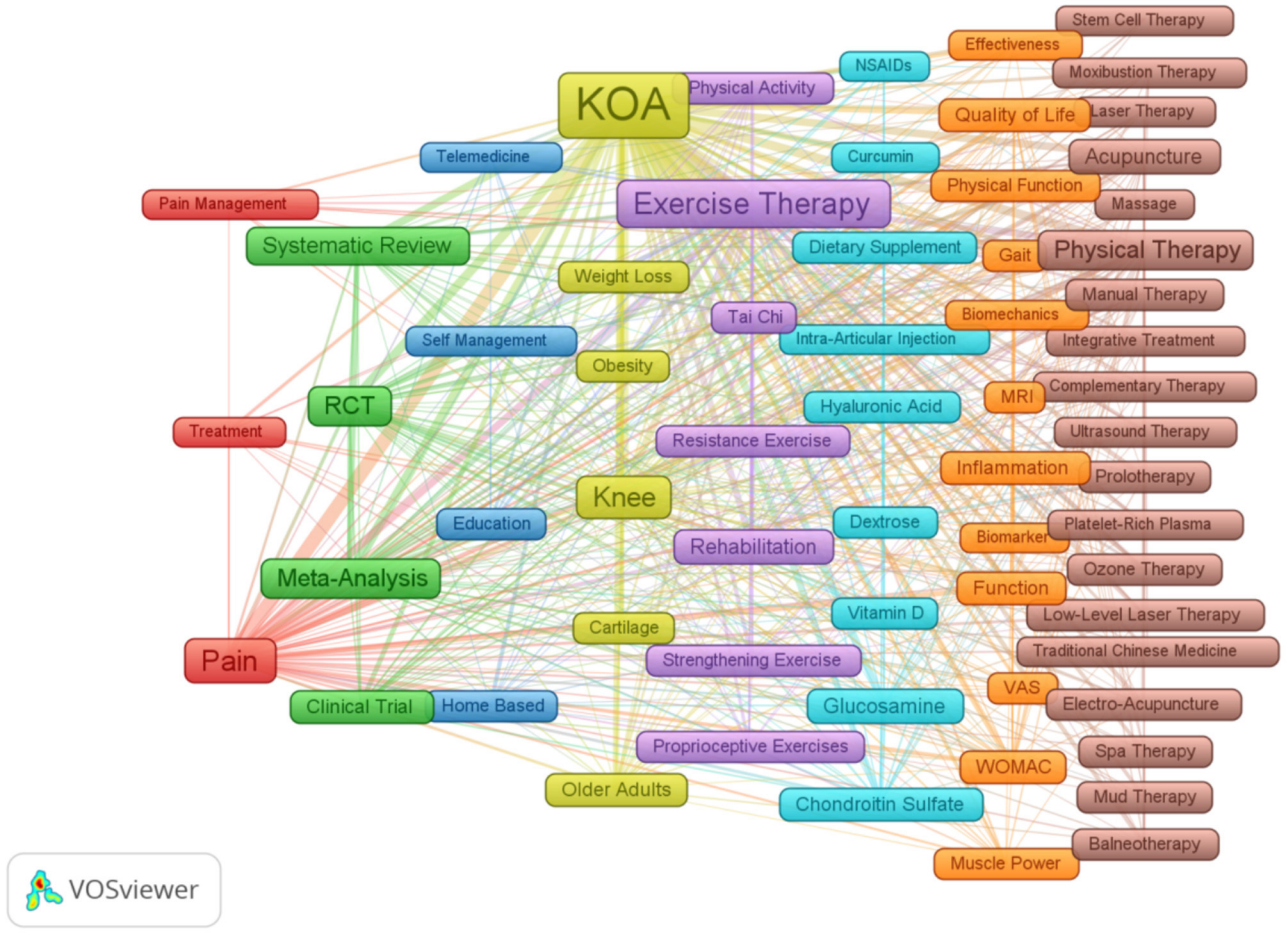
Network visualization of research themes related to KOA. This diagram illustrates the connections between various research themes in the context of KOA treatment, highlighting significant areas such as “Exercise Therapy,” “Pain Management,” and “Rehabilitation.” The nodes represent key topics, while the links indicate the relationships among them, showcasing the complexity and interdisciplinary nature of the research landscape. The same color indicates the same or similar research topic.

We utilized VOSviewer and Pajek software to construct a keyword clustering view, setting the co-occurrence threshold at 10 to reveal the distribution of research themes (Figure 7). In this view, the nodes represent 64 high-frequency keywords that appeared at least 10 times, while the lines connecting the nodes indicate the strength of their associations. The colors of the nodes signify different clusters with similar themes. Based on the grouping of different colors in the clusters, we summarized the following eight research themes: (1) Pain Management and Treatment (Red Cluster). (2) Research and Evidence-Based Practice (Green Cluster). (3) Telehealth and Education (Blue Cluster). (4) Demographics and Populations (Yellow Cluster). (5) Exercise and Rehabilitation (Purple Cluster). (6) Nutritional and Medical Interventions (Cyan Cluster). (7) Assessment and Measurement Tools (Orange Cluster). (8) Therapies and Treatment Modalities (Brown Cluster).

### 3.7 Thematic evolution and future research directions

**Figure 8:**
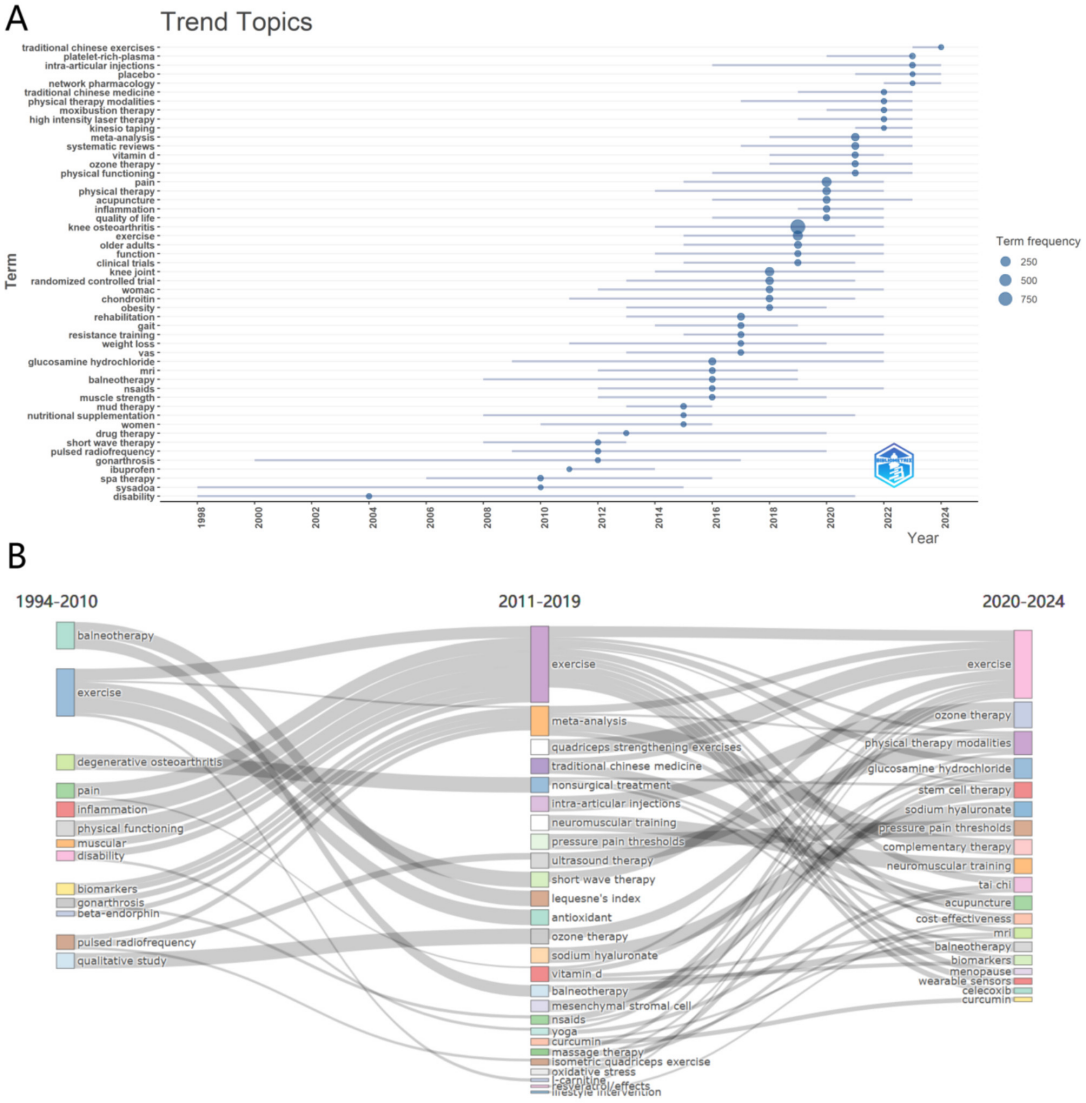
Trend theme and thematic evolution. **(A)** Showed the frequency of various research topics from 1998 to 2024. Each bubble represents a specific research theme, with the size of the bubble being proportional to the frequency of keyword occurrences. Additionally, the gray bars indicate the first and third quartiles of frequency distribution. **(B)** Illustrated the changes and evolution of topics from 1994 to 2024. It is divided into three time periods: 1994–2010, 2011–2019, and 2020–2024. The width of the connecting lines indicates the strength of the associations between different topics.

Figure 8A visually depicted the temporal changes and trends in research topics. Through this illustration, we can see that certain topics have experienced significant growth since 1994, particularly after 2010. For instance, terms like “physical therapy,” “older adults,” “function,” and “exercise” reflect society's increasing focus on the aging population and corresponding health management. At the same time, research methods such as “randomized controlled trial,” “systematic reviews,” and “meta-analysis” have also shown steady growth in frequency, reflecting the growing emphasis on the development and application of evidence-based medicine. Furthermore, apart from traditional themes of physical therapy and elderly health, the increasing occurrences of related terms such as “vitamin D,” “nutritional supplementation,” “pain,” and “inflammation” indicate a modern reconsideration of the relationship between nutrition and health, as well as a multi-faceted approach to disease management. The upward trend of themes related to “traditional Chinese medicine,” “traditional Chinese exercise,” and “moxibustion therapy” indicates increasing openness to discuss these alternative therapies. This may be due to a recognition of the limitations of traditional treatment methods and a greater emphasis on the patient's overall wellbeing.

Figure 8B further revealed how research themes have evolved through the flow direction lines with varying widths, demonstrating thematic changes and developments from 1994 to 2024 (divided into three periods: 1994–2010, 2011–2019, and 2020–2024). The width of the connecting lines between keywords represents the strength of associations between different themes. It can be observed that from 1994 to 2010, the research primarily focused on themes related to “balneotherapy,” “exercise,” “pain,” and “pulsed radiofrequency,” emphasizing attention to basic health management for patients with KOA. From 2011 to 2019, the term “exercise” continued to occupy a central position, highlighting its recognition as a core clinical intervention for KOA management. While not classified as CAIMT, its sustained research focus reflects the investment in promoting physical function and health within this field. At the same time, new research hotspots such as “meta-analysis,” “intra-articular injections,” “neuromuscular training,” “non-surgical treatment,” “ozone therapy,” “quadriceps strengthening exercises,” “traditional Chinese medicine,” “sodium hyaluronate,” and “mesenchymal stromal cell” began to emerge, indicating that researchers started to shift from singular non-pharmacological interventions to the exploration of complex therapies. Between 2020 and 2024, the appearance of keywords like “physical therapy modalities,” “complementary therapy,” “stem cell therapy,” “tai chi,” and “acupuncture” reflects modern medicine's increasing focus on alternative therapies and integrative treatments, leading to a new wave of research enthusiasm. In addition, the research topics have further encompassed aspects related to biology and economics, such as “biomarkers” and “cost-effectiveness,” indicating a growing focus on personalized medicine and economic considerations. Overall, the evolution path illustrated in Figure 8B demonstrated the dynamic shift from early singular treatment methods to the current comprehensive and personalized medical strategies.

### 3.8 Application of CAIMT in KOA

#### 3.8.1 Acupuncture

Acupuncture, as a traditional Chinese medicine therapy, has shown positive effects in the treatment of KOA. Multiple high-quality studies have confirmed the effectiveness of acupuncture in alleviating symptoms of KOA (25, 26). A review article noted that acupuncture may alleviate pain and promote functional recovery in patients with KOA through mechanisms such as inhibiting the overexpression of inflammatory factors like IL-1β, IL-6, and TNF-α, suppressing the activity of the MAPK signaling pathway to reduce inflammation, promoting the release of antioxidants, and inhibiting the hypertrophic differentiation of chondrocytes (27). A randomized controlled trial (RCT) assessed acupuncture as an adjunct therapy to standard care (28). The results demonstrated that acupuncture significantly improved function and alleviated pain in KOA patients. This finding is supported by other studies; for instance, a systematic review pointed out that acupuncture does provide benefits to KOA patients in the short term (29). However, further research is needed on the long-term effects and mechanisms of acupuncture.

#### 3.8.2 Mind-body practices

Mind-body practices, especially Tai Chi, has demonstrated significant effects in the treatment of KOA as a low-intensity and easy-to-implement form of exercise. A study evaluating the effects of Tai Chi on elderly KOA patients used the WOMAC scale to assess pain, and the results showed that Tai Chi practice significantly reduced pain (30). Tai Chi not only improves pain symptoms but also enhances cardiovascular health, muscle strength, and balance, while helping to alleviate stress, anxiety, and depression, ultimately improving health-related quality of life (31). Another study explored the mechanisms of mind-body practices and found that exercise can reduce inflammation, delay cartilage and bone degeneration, the change tendon, and muscle structure (32). This provides a theoretical basis for the application of mind-body practices in the treatment of KOA. However, there remains controversy regarding the optimal implementation strategies for Tai Chi and other forms of mind-body practices (such as frequency, intensity, and duration), necessitating further research (33).

#### 3.8.3 Physical therapies

The application of physical therapy in KOA is widely recognized, encompassing a range of methods such as electrotherapy, thermotherapy, ultrasound therapy, and manual therapy, aimed at alleviating pain, improving joint function, and enhancing the strength of surrounding muscles. Studies have shown that these treatment modalities not only effectively relieve KOA symptoms but also help prevent further deterioration of the condition by promoting blood circulation and enhancing joint stability (34). Moreover, the integration of physical therapy with exercise therapy can establish a more comprehensive treatment strategy, significantly improving patients' quality of life (35). Therefore, physical therapy represents an important component in the management of KOA and holds great clinical application potential, warranting further research into its optimal implementation strategies and long-term effects (36).

#### 3.8.4 Herbal medicine and nutritional supplements

Herbs and nutritional supplements are gradually gaining attention in the management of KOA, with many studies exploring their potential in alleviating pain and improving joint function. For example, curcumin found in turmeric is believed to have significant anti-inflammatory properties that can relieve joint pain and stiffness (37), while omega-3 fatty acids have been shown to reduce inflammatory responses and may improve joint mobility (38). Glucosamine and chondroitin are considered safe and have been reported to provide symptom relief (39). Despite these positive preliminary results, more high-quality randomized controlled trials are needed to verify the effectiveness and safety of these herbs and nutritional supplements. Although the research results on vitamin D supplementation in osteoarthritis show differences, it is currently recommended to supplement only in cases of vitamin D deficiency (40).

#### 3.8.5 Other complementary therapies

Other complementary therapies such as cryotherapy, thermotherapy, massage, and electrical stimulation have also been widely applied. These therapies provide additional relief for patients by alleviating pain, improving joint function, and promoting blood circulation. Research shows that cryotherapy can effectively reduce inflammation and swelling (41), while thermotherapy helps relieve muscle tension and increase joint flexibility (42). In addition, massage and electrical stimulation have also been found to effectively reduce pain and improve quality of life (43, 44). In recent years, the application of regenerative medicine therapies in the treatment of KOA has garnered widespread attention. Intra-articular injections of platelet-rich plasma (PRP) and stem cell therapy have shown certain potential. However, the long-term safety and efficacy of these regenerative medicine therapies still require further investigation.

#### 3.8.6 Integrative therapy

The integration of various complementary therapy regimens is receiving increasing attention. A systematic review analyzed the application of Oriental Medicine (OM) as both a standalone treatment and an adjunct therapy in knee osteoarthritis (KOA). The results indicated that compared to conventional treatments, standalone OM treatment had a “small effect” on symptom improvement (SMD = 0.48), whereas integrated treatment demonstrated a “very large effect” (SMD = 1.52) (45). In addition, a review study on multimodal multidisciplinary management indicated that treatment for moderate to severe KOA pain should adopt a multimodal, multicomponent, multidisciplinary approach, which includes various combinations of non-pharmacological and pharmacological interventions. This may involve the combined use of symptom-modifying slow-acting drugs (SYSADOAs) with NSAIDs, NSAIDs with weak opioids, or SYSADOAs/NSAIDs with intra-articular treatments (46). This suggests that combining complementary therapies with conventional treatments may produce synergistic effects and enhance overall therapeutic outcomes.

## 4 Discussion

The study reviewed the research status of CAIMT for KOA from the database's inception 1994 to 2024 through bibliometric analysis and narrative review, revealing several important findings. Firstly, the research output on CAIMT in KOA has shown steady growth (Figure 2), with modalities such as acupuncture being repeatedly validated for their significant efficacy. Notably, while exercise therapy is not classified as CAIMT but rather recognized as a core clinical intervention in guidelines (47), its parallel research trajectory underscores the scientific community's prioritization of multimodal approaches integrating both conventional and complementary strategies for KOA management. Secondly, “traditional Chinese medicine,” “traditional Chinese exercise,” the keyword co-occurrence cluster analysis suggested eight potential research themes, encompassing pain management, exercise and rehabilitation, nutritional interventions, and evidence-based practices (Figure 7). The high-frequency keyword distribution distinctly reflects scholars' concentrated research priorities, with exercise intervention optimization and pain management mechanisms emerging as the two most prominent foci (Figure 6). Thematic evolution analysis further revealed a progressive shift in research paradigms, transitioning from traditional therapeutic approaches to comprehensive, personalized management frameworks (Figure 8). Finally, the literature review indicates that acupuncture, physical therapy, mind-body practices and Tai Chi have shown good clinical effects in alleviating pain and improving function, gradually being recognized as beneficial complements to traditional treatments. The increase in literature may be closely related to the rising global demand for non-pharmacological treatments. The high productivity efficiency of core authors, along with close collaboration between countries worldwide and financial support (Figures 3, 4), has driven the development of this research field. In summary, we can see that research in the field of KOA has evolved from an initial stage focused on basic therapies, transitioned toward integrated treatments, and is now entering a phase that emphasizes individualized and evidence-based research.

The results of this study are consistent with some findings in the literature. The observed trends reflected society's emphasis on health management for the elderly population (48, 49), particularly in the diversification of treatment methods. With the global aging population and rising obesity rates, the incidence of KOA continues to increase (50, 51). The limitations of conventional treatment have prompted researchers to explore safer and more effective alternatives. For example, acupuncture has been widely proven to effectively alleviate pain and improve function in patients with KOA, which aligns with existing systematic reviews (52, 53). Furthermore, mind-body practices, especially Tai Chi, not only helps to improve knee joint function, enhance muscle strength, and regulate balance but also has positive effects on cardiovascular and mental health (54, 55). Meanwhile, the gradual recognition of herbs and nutritional supplements indicates a preference among patients for non-pharmacological treatments, reflecting their demand for personalized and non-invasive therapies. Although the effectiveness results of different studies vary, overall, CAIMT is significantly associated with improvements in patient's quality of life and symptom relief. The integration of various non-pharmacological treatment methods shows better therapeutic effects compared to conventional therapies, as evidenced by the enhancement of quality of life and functional levels (56). This trend reflects evolving clinical practice patterns that integrate guideline-endorsed core therapies with complementary approaches. Recent consensus guidelines, such as the NICE guidelines (47), prioritize exercise therapy and weight management as first-line treatments while acknowledging select CAM modalities (e.g., acupuncture, mind-body practices) as adjunctive options for specific patient subgroups. Concurrently, the field demonstrates methodological maturation through increased utilization of randomized controlled trials (57) and meta-analyses (58) to evaluate CAM interventions, signaling a commitment to evidence-based integration rather than uncritical adoption.

### 4.1 Advantages and limitations

The methodological integration in this study offers unique advantages. By combining bibliometric analysis (quantitative mapping of 2,137 publications) with narrative review, we have achieved dual objectives: (1) identifying macro-level research trends, collaboration networks, and knowledge gaps through data-driven metrics, and (2) contextualizing these patterns via critical of therapeutic mechanisms, efficacy evidence, and implementation challenges reported in key studies. This dual-lens approach, detailed in Sections 2.3 and 3.4, provides a framework for translational prioritization—helping researchers focus on understudied CAIMT modalities (e.g., mind-body therapies) while equipping clinicians with evidence hierarchies to guide personalized treatment decisions.

However, three limitations require acknowledgment:

(1) Database selection bias: Restricting data sources to Web of Science, while justified for bibliometric consistency (Section 2.1), may exclude regionally focused studies from PubMed or Scopus. For instance, traditional Chinese medicine research disproportionately indexed in CNKI was underrepresented.
(2) Language and evidence granularity: The English-only focus overlooks potentially significant non-English trials, particularly for region-specific therapies like Ayurveda. Furthermore, while bibliometrics reveal publication trends, they cannot assess individual study quality—a gap partially addressed but not fully resolved by our narrative review.
(3) Mechanistic and longitudinal evidence gaps: as shown in Figure 6, only 12% of CAIMT studies investigated long-term (>1 year) outcomes, and mechanistic research constituted merely 8% of the corpus. This underscores the need for preclinical-translational studies to validate observed clinical effects.

## 5 Conclusion

In summary, the study employed bibliometric analysis and narrative review to examine the research landscape of complementary, alternative, and integrative medicine therapies (CAIMT) for knee osteoarthritis (KOA) from the database's inception 1994 to 2024. Utilizing metrics such as the h-index, g-index, and m-index, alongside visualization tools, we identified eight key research clusters, including pain management, exercise therapy, nutritional interventions, and evidence-based practices. The analysis revealed evolving trends, and highlighted the growing emphasis on biomarkers, telehealth, and cost-effectiveness in recent years.

Our findings demonstrate the increasing prominence of non-pharmacological therapies like acupuncture, Tai Chi, and physical therapies in alleviating KOA symptoms and improving patient outcomes. However, critical gaps persist, including limited mechanistic studies (8% of the corpus), insufficient long-term efficacy data (12% addressing outcomes beyond 1 year), and underrepresentation of non-English and region-specific research due to database and language restrictions. These limitations highlight the need for future investigations.

Future research directions may involve the following aspects: (1) Integrating traditional and modern treatment strategies: combining traditional therapies with biotechnological approaches to enhance treatment efficacy. (2) Application of biomarkers: exploring the potential for personalized treatment through biomarkers to monitor patient responses. (3) Cost-effectiveness analysis: conducting economic evaluations of different therapies to optimize resource allocation and treatment strategies. (4) Strengthening evidence-based research: continuing to adopt high-quality randomized controlled trials and systematic reviews to provide a more solid scientific foundation for clinical practice.

## Data Availability

The raw data supporting the conclusions of this article will be made available by the authors, without undue reservation.

## Footnotes

^1^WHO. *Traditional, complementary and integrative medicine*. World Health Organization.
