# Supplementary material for: Complementary, alternative, and integrative medicine therapies for knee osteoarthritis: a visual analytics analysis of the research domain
Source: Front Med (Lausanne). 2025 Jun 11;12:1586357. doi: 10.3389/fmed.2025.1586357 (PMC12187784; doi:10.3389/fmed.2025.1586357)
Supplement: Supplementary file 1 [file Data_Sheet_1.pdf]

Web of Science Search Strategy (v0.1)

Database: Web of Science Core Collection

Entitlements:

- WOS.IC: 1993 to 2024
- WOS.CCR: 1985 to 2024
- WOS.SCI: 1994 to 2024

Searches:

1: TI＝("5-Hydroxytryptophan" or "2-Amino-3-(5-hydroxy-1H-Indol-3-yl)-Propanoic Acid" OR "L-5 Hydroxytryptophan" OR "5 Hydroxy-Tryptophan" OR "5 Hydroxy-Tryptophane" OR "5-Hydroxytryptophane" OR "5-Hydroxy L-Tryptophan" OR "5-Hydroxy L-Tryptophane" OR "5-Hydroxy Tryptophan" OR "5-L-Hydroxytryptophan" OR "L-5 HTP" OR "L-5-Hydroxytryptophan" OR "L-5-Hydroxytryptophane" OR "Oxitriptan" OR "714-X" OR "Abscisic Acid" OR "Acai" OR "Euterpe oleracea" OR "E oleracea" OR "Euterpe badiocarpa" OR "E badiocarpa" OR "Assai" OR "Baie de Palmier Pinot" OR "Cabbage Palm" OR "Chou Palmiste" OR "Palmier d Acai" OR "Active Therap*" OR "Acumoxa" OR "Acupressure" OR "Acupresion" OR "Acupression" OR "Nei Guan" OR "Self-Acupressure" OR "Acupuncture" OR "Acuponcture" OR "Acupuntura" OR "Electroacupuncture" OR "Needle Moxibustion" OR "Acustimulation" OR "Acupoint Stimulation" OR "Transcutaneous Acupoint Electrical Stimulation" OR "Transcutaneous Electrical Acupoint Stimulation" OR "Transcutaneous Electrical Acustimulation" OR "Acutonics" OR "Aerobics" OR "Agrimony" OR "A eupatoria" OR "Agrimone" OR "Agrimonia" OR "Aigremoine" OR "A Eupatoire" OR "Church Steeples" OR "Churchsteeples" OR "Cockeburr" OR "Cocklebur" OR "Da Hua Long Ya Cao" OR "Eupatoire-des-Anciens" OR "Francormier" OR "Herba Agrimoniae" OR "Herbe-de-Saint-Guillaume" OR "Philanthropos" OR "Soubeirette" OR "Sticklewort" OR "The des Bois" OR "The du Nord" OR "Toute-Bonne" OR "Aikido" OR "Gendai Budo" OR "Kata" OR "Aiyishu" OR "Alcoholics Anonymous" OR "Alexander Technique" OR "Alexander Proprioception" OR "Technique Alexander" OR "Tecnica Alexander" OR "Allicin" OR "Aloe" OR "A vera" OR "A barbadensis" OR "A indica" OR "A africana" OR "A arborescens" OR "A natalenis" OR "A frutescens" OR "A ucriae" OR "A ferox" OR "A supralaevis" OR "A perryi" OR "A spicata" OR "A Capensis" OR "A Perfoliata" OR "Aloes" OR "Burn Plant" OR "Chritkumari" OR "Elephant's Gall" OR "Gel de la Feuille d'Aloes" OR "Ghee-Kunwar" OR "Ghi-Kuvar" OR "Ghrita-Kumari" OR "Gvar Patha" OR "Hsiang-Dan" OR "Kanya" OR "Kumari" OR "Latex d'Aloes" OR "Lily of the Desert" OR "Lu-Hui" OR "Miracle Plant" OR "Plant of Immortality" OR "Plante de l Immortalite" OR "Plante de la Peau" OR "Plante de Premiers Secours" OR "Plante Miracle" OR "Plantes des Brulures" OR "Sabila" OR "Alpha-Linolenic Acid" OR "Acide Alpha-Linolenique" OR "Acido Alfa Linolenico" OR "Acide Gras Essentiel" OR "Acide Linolenique" OR "Acide Gras N3" OR "Acide Gras Polyinsature N3" OR "Linolenic Acid" OR "Omega 3" OR "Omega-3" OR "Linum usitatissimum" OR "L usitatissimum" OR "Linum crepitans" OR "L crepitans" OR "Linum humile" OR "L humile" OR "Aceite de Linaza" OR "Acide Alpha-Linolenique" OR "Acide Gras N-3" OR "Aliviraaii" OR "Alasi"

OR "Echter Lein" OR "Flachs" OR "Flachssamen" OR "Graine De Lin" OR "Huile de Lin" OR "Kattan" OR "Keten" OR "Lin" OR "Linho" OR "Lino" OR "Linseed Oil" OR "Malsag" OR "Saatlein" OR "Ta Ma" OR "Tisii" OR "Alpha-Lipoic Acid" OR "1,2-dithiolane-3-pentanoic acid" OR "1,2-dithiolane-3-valeric acid" OR "6,8-thioctic acid" OR "5-(1,2-dithiolan-3-yl) valeric acid" OR "6,8-dithiooctanoic acid" OR "A-Lipoic Acid" OR "Acetate Replacing Factor" OR "Acide Alpha-Lipoique" OR "Acide DL-Alpha- Lipoique" OR "Acide Lipoique" OR "Acide Thioctique" OR "Acide 1,2-dithiolane-3-pentanoique" OR "Acide 1,2-dithiolane-3-valerique" OR "Acide 5 Valerique (1,2-dithiolan-3-yl)" OR "Acide 6,8-dithiooctanoique" OR "Acide 6,8-Thioctique" OR "Acido Alfa Lipoico" OR "Biletan" OR "DHLA" OR "Dihydrolipoic acid" OR "Extrait d'acide Alpha-Lipoique" OR "Lipoic Acid" OR "Lipoicin" OR "R-ALA" OR "R-Alpha-Lipoic Acid" OR "1,2-Dithiolane-3-Pentanoic Acid" OR "R-Dithiolane-3-Pentanoic Acid" OR "R-Lipoic Acid" OR "RS-Alpha-Lipoic Acid" OR "S- Alpha-Lipoic Acid" OR "S-Lipoic Acid" OR "Sodium-R-Lipoate" OR "Thioctacid" OR "Thioctan" OR "Thioctic Acid" OR "Alternative Medicine" OR "Alternative Therap*" OR "Alternative Approach" OR "Alternative Health" OR "Amino Acid Therap*" OR "Amma Therap*" OR "Ammotherap*" OR "Ancient Medicine" OR "Androstenedione" OR "4-androstene-3,17-dione" OR "Androst-4-ene-3,17-dione" OR "4-Androstene 3, 17-dione" OR "Androstene" OR "Androstenediona" OR "Animal Assisted Therap*" OR "Animal-Assisted Activities" OR "Animal-Assisted Education" OR "Animal-Assisted Intervention" OR "Animal-Facilitated Therap*" OR "Animal Companionship" OR "Animals In Human Therap*" OR "Animal Visitation" OR "Canine-Assisted Ambulation" OR "Canine-Assisted Therap*" OR "Canine Therap*" OR "Canine Visitation Therap*" OR "Companion Animal Therap*" OR "Dog-Assisted Therap*" OR "Dolphin-Assisted Therap*" OR "Equine-Assisted Activity" OR "Equine-Assisted Activities and Therapies" OR "Equine-Assisted Therap*" OR "Equine Therap*" OR "Hippotherap*" OR "Horse-Riding Therap*" OR "Pet-Assisted Therap*" OR "Pet-Facilitated Therap*" OR "Pet Therap*" OR "Psychoeducational Horseback Riding" OR "Therapeutic Horseback Riding" OR "Animal Extract" OR "Aniministic Practice" OR "Anma" OR "Anthocyanin" OR "Anthrophysical Medicine" OR "Anthroposophic Medicine" OR "Antineoplastons" OR "3-Phenylacetylamino-2,6-piperidinedione" OR "Phenylacetate" OR "Phenylacetylglutamine" OR "Phenylacetylisoglutamine" OR "Antineoplaston A" OR "Antineoplaston A1" OR "Antineoplaston A10" OR "Antineoplaston A10-1" OR "Antineoplaston A2" OR "Antineoplaston A3" OR "Antineoplaston A4" OR "Antineoplaston A5" OR "Antineoplaston AS2-1" OR "Antineoplaston AS2-5" OR "Antineoplaston AS5" OR "Antineoplaston Ch" OR "Antineoplaston F" OR "Antineoplaston H" OR "Antineoplaston K" OR "Antineoplaston L" OR "Antineoplaston O" OR "Antioxidant" OR "Apitherap*" OR "Apipuncture" OR "Apis Mellifera Venom" OR "Apis Venenum Purum" OR "Apiterapia" OR "Api-Therap*" OR "Api-Treatment" OR "Apitherapie" OR "Api-Venin-Therapie" OR "Bee Sting Therap*" OR "Bee Therap*" OR "Bee Treatment" OR "Bee Venom Therap*" OR "Therapie des Abeilles" OR "Therapie par Venin d'Abeille" OR "Traitement par Piqures d'Abeilles" OR "Applied Biomechanics" OR "Applied Kinesiology" OR "Health Kinesiology" OR "Kinesiologie Appliquee" OR "Kinesiologie Educative" OR "Kinesiology Muscle Test" OR "Quinesiologia" OR "Test Musculaire de la Kinesiologie" OR "Arachidonic Acid" OR "Aristolochia" OR "Aristoloche" OR "Aristoloquia" OR "Birthwort" OR "Guan Mu Tong" OR "Guang Fang Ji" OR "Pelican Flower" OR "Poison de Terre" OR "Pomerasse" OR "Ratelaine" OR "Sangree Root" OR "Sangrel" OR "Sarrasine" OR "Serpentaire" OR "Serpentaria" OR "Snakeroot" OR "Snakeweed" OR "Virginia Serpentary" OR "Arnica" OR "A montana" OR "A fulgens" OR "A

sororia" OR "A latifolia" OR "A cordifolia" OR "A angustifolia" OR "A chamissonis" OR "Arnikabluten" OR "Bergwohlverleih" OR "Doronic d'Allemagne" OR "Fleurs d'Arnica" OR "Kraftwurz" OR "Leopard's Bane" OR "Mountain Snuff" OR "Mountain Tobacco" OR "Plantin des Alpes" OR "Quinquina des Pauvres" OR "Souci des Alpes" OR "Tabac des Savoyards" OR "Tabac des Vosges" OR "Wolf's Bane" OR "Wolfsbane" OR "Wundkraut" OR "Aromatherap*" OR "Aroma" OR "Aromaterapia" OR "Aromatherapie" OR "Aromatic Oils" OR "Aromatic Therap*" OR "Essential Oils" OR "Huiles Aromatiques" OR "Huiles Essentielles" OR "Scent Therap*" OR "Traitement par les Essences de Plantes" OR "Art Therap*" OR "Art Stimulation" OR "Chinese Calligraphy Therap*" OR "Creative Arts Intervention" OR "Creative Arts Therap*" OR "Creative Expression" OR "Absinthe du Desert" OR "Common Wormwood" OR "Common Worm Wood" OR "Desert Wormwood" OR "Herba Alba" OR "Shih" OR "Artichoke" OR "Cynara cardunculus" OR "Cynara scolymus" OR "Alcachofa" OR "Alcaucil" OR "Artichaut" OR "Artischocke" OR "Cardo" OR "Cardon d'Espagne" OR "Cardoon" OR "Cynara" OR "Extrait d'Artichaut" OR "Feuille d'Artichaut" OR "Gemuseartischocke" OR "Kardone" OR "Tyosen-Azami" OR "Asian Diet" OR "Astragaloside" OR "Astragalus" OR "A membranaceus" OR "Phaca membranacea" OR "Astragale" OR "Astragali" OR "Astragalo" OR "Beg Kei" OR "Bei Qi" OR "Buck Qi" OR "Huang Qi" OR "Huang Se" OR "Huanggi" OR "Hwanggi" OR "Membranous Milkvetch" OR "Milk Vetch" OR "Mongolian Milk" OR "Mongolian Milkvetch" OR "Ogi" OR "Reglisse Batarde" OR "Reglisse Sauvage" OR "Atkins" OR "Dr Robert Atkins' Diet" OR "High Protein Diet" OR "Low-Carb Diet" OR "Low- Carbohydrate Diet" OR "Low-Carbohydrate High-Protein Diet" OR "Low Carb Diet" OR "Regime d'Atkins" OR "Regime Hyperproteine" OR "Regime Hyperprotidique" OR "Regime Hypoglucidique" OR "Atractylis gummifera" OR "Auriculotherap*" OR "Auricular Therap*" OR "Autogenic" OR "Autorelaxation Concentrative" OR "Autosuggestion" OR "Entrainement Autogene" OR "Entrenamiento Autogeno" OR "Methode de Schultz" OR "Relaxation Therap*" OR "Training Autoeene" OR "Aversion Therap*" OR "Ayurveda" OR "Ayurvedic Medicine" OR "Medecine Ayurvedique" OR "Medecine Traditionnelle Asiatique" OR "Medecine Traditionnelle Indienne" OR "Traditional Asian Medicine" OR "Ba Wei Di Huang Wan" OR "Shen Qi Wan" OR "Bach" OR "Batch Flower Remedies" OR "Floratherapie" OR "Flower Dilutions" OR "Flower Essence" OR "Flower Remedies" OR "Remedes Floraux" OR "Balance Technique" OR "Balneotherap*" OR "Bain de la Mer Morte" OR "Bain Mineral" OR "Bain de Soufre" OR "Bain Thermal" OR "Balneological Treatment" OR "Balneoterapia" OR "Balneotherapeutics" OR "Balneotherapie" OR "Balneum" OR "Bath Therap*" OR "Bath Treatment" OR "Crenobalneotherap*" OR "Dead Sea Baths" OR "Low-Dose Radon Hyperthermia Balneo Treatment" OR "Mineral Bath" OR "Spa Therap*" OR "Sulfur Baths" OR "Therapeutic Bathing" OR "Therapie Thermale" OR "Thermal Baths" OR "Thermal Mineral Baths" OR "Thermal Therap*" OR "Traitement par le Bain" OR "Water Therap*" OR "Laurus nobilis" OR "Bay Leaf" OR "Bay Tree" OR "Daphne" OR "Laurier d'Apollon" OR "Laurier Noble" OR "Laurier-Sauce" OR "Laurier Vrai" OR "Bear Bile" OR "Behaviour Medicine" OR "Berberine" OR "Berberina" OR "Umbellatine" OR "Beta-Carotene" OR "A-Beta-Carotene" OR "Beta-Caroteno" OR "Carotenes" OR "Carotenoids" OR "Provitamin A" OR "Provitamine A" OR "Bibliotherap*" OR "Bilberry" OR "Vaccinium myrtillus" OR "Airelle" OR "Arandano" OR "Black Whortles" OR "Bleaberry" OR "Brimbelle" OR "Burren Myrtle" OR "Dyeberry" OR "Gueule Noire" OR "Huckleberry" OR "Hurtleberry" OR "Mauret" OR "Myrtille" OR "Myrtilli Fructus" OR "Raisin des Bois" OR "Trackleberry" OR "Whortleberry" OR "Wineberry" OR "Biochemical Therap*" OR "Biodynamic Therap*" OR "Biofeedback" OR "Bio-retroaction" OR "Biorretroalimentacion" OR

"Neurofeedback" OR "Neuro-Retroaction" OR "Retroaction Biologique" OR "Retrocontrole Biologique" OR "Biofunctional Diagnostic Testing" OR "Biologic Treatment" OR "Bioresonance" OR "Appareil Mora" OR "Biocom" OR "Biocommunication" OR "Biophysical Information" OR "Biorresonancia" OR "Information Biophysique" OR "Mora Device" OR "Mora Therap*" OR "Moratherapie" OR "Multicom" OR "Multiresonance" OR "Therapie Mora" OR "Bitter Orange" OR "Citrus amara" OR "Citrus bigarradia" OR "Citrus vulgaris" OR "Aurantii Fructus" OR "Aurantii Pericarpium" OR "Aurantium" OR "Bigarade" OR "Chao Zhi Ke" OR "Chisil" OR "Extrait de Zeste d'Orange" OR "Fleur d'Orange Amere" OR "Flos Citri Auranti" OR "Fructus Aurantii" OR "Green Orange" OR "Kijitsu" OR "Marmalade Orange" OR "Meta-Synephrine" OR "N-Methyltyramine" OR "Naranja Amarga" OR "Neroli Oil" OR "Norsynephrine" OR "Octopamine" OR "Orange Amere" OR "Orange de Seville" OR "Orange Peel Extract" OR "Orange Verte" OR "Seville Orange" OR "Shangzhou Zhiqiao" OR "Sour Orange" OR "Synephrine" OR "Zeste d'Orange Amere" OR "Zhi Ke" OR "Zhi Qiao" OR "Zhi Shi" OR "Black Cohosh" OR "Actaea racemose" OR "Cimicifuga racemose" OR "Actaea macrotys" OR "Actee a Grappes" OR "Actee Noire" OR "Aristolochiaceae Noire" OR "Baie d'actee" OR "Baneberry" OR "Black Aristolochiaceae" OR "Bugbane" OR "Bugwort" OR "Cimicaire a grappes" OR "Cimicifuga" OR "Cimicifuge" OR "Cohosh Negro" OR "Cohosh Noir" OR "Cytise" OR "Macrotys" OR "Phytoestrogen" OR "Phytoestrogene" OR "Racine de Serpent" OR "Racine de Squaw" OR "Racine Noire de Serpents" OR "Rattle Root" OR "Rattle Top" OR "Rattlesnake Root" OR "Rattleweed" OR "Rhizoma Cimicifugae" OR "Sheng Ma" OR "Squaw Root" OR "Piper nigrum" OR "Hu Jiao" OR "Kali Mirchi" OR "Kosho" OR "Marich" OR "Maricha" OR "Miris" OR "Peber" OR "Peper" OR "Peppar" OR "Pepper" OR "Peppercorn" OR "Pfeffer" OR "Pimenta" OR "Pimienta" OR "Pipar" OR "Piper" OR "Piperine" OR "Pippuri" OR "Poivre" OR "Poivrier" OR "Vellaja" OR "Black Seed" OR "Nigella sative" OR "Ajenuz" OR "Aranuel" OR "Baraka" OR "Charnuska" OR "Cheveux de Venus" OR "Cominho Negro" OR "Comino Negro" OR "Fennel Flower" OR "Fitch" OR "Graine de Nigelle" OR "Graine Noire" OR "Habatul Sauda" OR "Habbatul Baraka" OR "Kalajaji" OR "Kalajira" OR "Kalonji" OR "Ketsah" OR "La Grainer Noire" OR "Love in a Mist" OR "Mugrela" OR "Nielle" OR "Nigelle de Crete" OR "Nigelle Cultivee" OR "Nutmeg Flower" OR "Poivrette" OR "Roman-Coriander" OR "Schwarzkummel" OR "Seed of Blessing" OR "Siyah Dane" OR "Shoniz" OR "Small Fennel" OR "Toute Epice" OR "Upakuncika" OR "Blueberry" OR "Blueberries" OR "Vaccinium angustifolium" OR "V angustifolium" OR "Vaccinium brittonii" OR "Vaccinium lamarckii" OR "Vaccinium pensylvanicum" OR "Vaccinium virgatum" OR "Vaccinium ashei" OR "Vaccinium amoenum" OR "Vaccinium corymbosum" OR "Vaccinium constablaei" OR "Vaccinium pallidum" OR "Vaccinium altomontanum" OR "Vaccinium vacillans" OR "V brittonii" OR "V lamarckii" OR "V pensylvanicum" OR "V virgatum" OR "V ashei" OR "Vamoenum" OR "V corymbosum" OR "V constablaei" OR "V pallidum" OR "V altomontanum" OR "V vacillans" OR "Arandano" OR "Bleuet" OR "Bleuets" OR "Myrtille" OR "Rubel" OR "Tifblue" OR "Bobath" OR "Body Control" OR "Body Manipulation" OR "Body Electronics" OR "Bodywork" OR "Bone Setting" OR "Borage" OR "B officinalis" OR "Bee Plant" OR "Beebread" OR "Borago" OR "Borraja" OR "Bourrache" OR "Burage" OR "Burrage" OR "Common Bugloss" OR "Cool Tankard" OR "Langue de Boeuf" OR "Ox's Tongue" OR "Pain-des-Abeilles" OR "Starflower" OR "Talewort" OR "Boron" OR "Atomic number 5" OR "Acide Borique" OR "Anhydride Borique" OR "B symbole chimique" OR "Borate" OR "Borates" OR "Boric Acid" OR "Boric Anhydride" OR "Boric Tartrate" OR "Numero Atomique 5" OR "Botanicals" OR "Bowen Technique" OR "Bowen Manipulative Therap*" OR "Bowen Therap*" OR "Methode Bowen" OR "Tecnica Bowen" OR "Technique

Bowen" OR "Technique de Bowen" OR "Therapeute en Bowen" OR "Therapie Manuelle" OR "Bromelain" OR "A comosus" OR "Pineapple" OR "A duckei" OR "A sativus" OR "Bromelia ananas" OR "Bromelia comosa" OR "B comosa" OR "Ananas" OR "Bromelaine" OR "Bromelains" OR "Bromelainum" OR "Bromelin" OR "Bromelina" OR "Bromeline" OR "Enzyme d'Ananas" OR "Extrait d'Ananas" OR "Fruit Bromelain" OR "Protease" OR "Brown Peterson Technique" OR "Buddhist Tantric Practice" OR "Burdock" OR "A minus" OR "A tomentosum" OR "Arctium" OR "Bardana" OR "Bardana-Minor" OR "Bardanae Radix" OR "Bardane" OR "Beggar's Buttons" OR "Burr Seed" OR "Clotbur" OR "Cocklebur" OR "Cockle Buttons" OR "Fox's Clote" OR "Glouteron" OR "Grande Bardane" OR "Great Bur" OR "Great Burdocks" OR "Happy Major" OR "Hardock" OR "Harebur" OR "Lappa" OR "Love Leaves" OR "Niubang" OR "Niu Bang Zi" OR "Orelha-de-Gigante" OR "Personata" OR "Philanthropium" OR "Rhubarbe du Diable" OR "Thorny Burr" OR "Buteyko Breathing Technique" OR "Buteyko Breathing Training" OR "Buteyko Institute Method" OR "Buteyko Method" OR "Buteyko Technique" OR "Eucapnic Breathing Technique" OR "Eucapnic Buteyko Breathing" OR "Butterbur" OR "Petasites" OR "P hybridus" OR "P officinalis" OR "Tussilago hybrida" OR "Blatterdock" OR "Bog Rhubarb" OR "Bogshorns" OR "Butter Bur" OR "Butter-Dock" OR "Butterfly Dock" OR "Capdockin" OR "Chapeliere" OR "Contre-Peste" OR "Exwort" OR "Flapperdock" OR "Grand Bonnet" OR "Langwort" OR "Pestwurz" OR "Petasite" OR "P Hybride" OR "P Officinal" OR "P Vulgaire" OR "P Vulgaris" OR "Petasitidis Folium" OR "Petasitidis Hybridus" OR "Petasitidis Rhizoma" OR "Plague Root" OR "Umbrella Leaves" OR "P Folium" OR "P Hybridus" OR "P Rhizoma" OR "Bwiti" OR "Calcium" OR "Bone Meal" OR "Calcarea Carbonica" OR "Calcarea Phosphorica" OR "Calcio" OR "Coquilles d'Huitres Moulues" OR "Coquilles d'oeuf" OR "Di-Calcium Phosphate" OR "Dolomite" OR "Hydroxyapatite" OR "Microcrystalline Hydroxyapatite" OR "OsseinHydroxyapatite" OR "Oyster Shell" OR "Phosphate Tricalcium" OR "Poudre d'os" OR "Tricalcium Phosphate" OR "Calendula" OR "C officinalis" OR "Calendule" OR "Fleur de Tous les Mois" OR "Gold-Bloom" OR "Holligold" OR "Marigold" OR "Marybud" OR "Souci des Champs" OR "Souci des Jardins" OR "Souci des Vignes" OR "Souci Officinal" OR "Zergul" OR "Calorie Control" OR "Cannabis" OR "Cannabinoids" OR "C sativa" OR "Anashca" OR "Banji" OR "Bhang" OR "Blunt" OR "Charas" OR "Esrar" OR "Gaga" OR "Ganga" OR "Grass" OR "Haschisch" OR "Hashish" OR "Herbe" OR "Huo Ma Ren" OR "Marie-Jeanne" OR "Mariguana" OR "Marihuana" OR "Marijuana" OR "Mary Jane" OR "Sawi" OR "Sinsemilla" OR "Weed" OR "Cannabidiol" OR "Tetrahydrocannabinol" OR "Capsaicin" OR "Capsicum" OR "C frutescens" OR "C annuum" OR "C chinense" OR "C baccatum" OR "C pubescens" OR "C minimum" OR "African Chillies" OR "Aji" OR "Capsaicine" OR "C Oleoresin" OR "Cayenne" OR "Chili" OR "Chilli" OR "Chillies" OR "Cis-capsaicin" OR "Civamide" OR "Goat's Pod" OR "Grains of Paradise" OR "Ici Fructus" OR "Katuvira" OR "Mexican Chilies" OR "Mirchi" OR "Paprika" OR "Pili-pili" OR "Piment Enrage" OR "Piment Fort" OR "Piment-oiseau" OR "Pimento" OR "Trans-Capsaicin" OR "Zucapsaicin" OR "Zucapsaicine" OR "Caraway" OR "Carum carvi" OR "Carum velenovskyi" OR "C carvi" OR "C velenovskyi" OR "Alcaravea" OR "Anis Canadien" OR "Anis des Pres" OR "Anis des Vosges" OR "Carraway" OR "Carvi" OR "C Commun" OR "C Fructus" OR "Faux Anis" OR "Haravi" OR "Jeera" OR "Jira" OR "Karwiya" OR "Krishan Jeeraka" OR "Krishnajiraka" OR "Kummel" OR "Kummich" OR "Semen Cumini Pratensis" OR "Shahijra" OR "Shiajira" OR "Wiesen-Feldkummel" OR "Carbohydrate Restricted Diet" OR "L-Carnitine" OR "3-carboxy-2-hydroxy-N,N,N-trimethyl-1-propanaminium inner salt" OR "(3-carboxy2-hydroxypropyl) trimethylammonium hydroxide inner salt" OR

"B-hydroxy-Ntrimethyl aminobutyric acid" OR "Beta-hydroxy-gammatrimethylammonium butyrate" OR "L-3-hydroxy-4-(trimethylammonium)-butyrate" OR "(R)-(3-carboxy-2 hydroxypropyl)trimethylammonium hydroxide" OR "(R)-3-hydroxy-4-trimethylammonio-butyrate" OR "3-hydroxy-4-Ntrimethylaminobutyrate" OR "Aminocarnitine" OR "Beta-hydroxyl-gamma-tributyl aminobutyrate" OR "B(t) Factor" OR "Carnitine" OR "Carnitor" OR "D-Carnitine" OR "DLCarnitine" OR "Facteur B(t)" OR "L-b-hydroxy-c-N-trimethylaminobutyric acid" OR "L-Carnitina" OR "Levocarnitine" OR "Vitacarn" OR "Vitamin B(t)" OR "Vitamine B(t)" OR "Acetyl-L-Carnitine" OR "2-(acetyloxy)-3-carboxy-N,N,N-trimethyl-1-propanaminium inner salt" OR "(3-carboxy-2-hydroxypropyl)trimethylammonium hydroxide inner salt acetate" OR "Acetil-L-Carnitina" OR "Acetyl-LCarnitine HCl" OR "Acetyl L-Carnitine Hydrochloride" OR "Acetyl Carnitine" OR "Acetyl-Carnitine" OR "Acetyl-Levocarnitine" OR "Aminocarnitine" OR "Carnitine Acetyl Ester" OR "Dihydrochlorure d'Acetyl-L-Carnitine Arginate" OR "Gamma-Trimethyl-BetaAcetylbutyrobetaine" OR "L-Acetylcarnitine" OR "Levacecarnine" OR "N-Acetyl-Carnitine" OR "N-AcetylCarnitine Hydrochloride" OR "N-Acetyl-L-Carnitine" OR "Vitamin B(t) Acetate" OR "Cartilage Supplement" OR "Shark Cartilage" OR "Squalus acanthias" OR "Spiny Dogsh Shark" OR "Sphyrna lewini" OR "Scalloped Hammerhead Shark" OR "AE-941" OR "Cartilage de Requin" OR "Cartilago de Tiburon" OR "Collagene Marin" OR "Extrait de Cartilage de Requin" OR "Liquide de Cartilage Marin" OR "Marine Collagen" OR "Marine Liquid Cartilage" OR "Neovastat" OR "Poudre de Cartilage de Requin" OR "Bovine Cartilage" OR "Antitumor Angiogenesis Factor" OR "Bovine Tracheal Cartilage" OR "Cartilage Tracheal de Bovins" OR "Cartilago Bovino" OR "Catrix" OR "Catrix-S" OR "Collagen Bovine" OR "Collagene Bovin" OR "Glycosaminoglycan Polysulphuric Acid Complex" OR "Psoriacin" OR "Psoriacin-T" OR "Rumalon" OR "Casein Free Diet" OR "Catgut Implantation" OR "Cat's Claw" OR "Uncaria guianensis" OR "Uncaria tomentosa" OR "U guianensis" OR "U tomentosa" OR "Griffe Du Chat" OR "Liane du Perou" OR "Life-Giving Vine of Peru" OR "Peruvian Liana" OR "Samento" OR "Una De Gato" OR "Cellasene" OR "Cetyl Myristoleate" OR "Chamomile" OR "Chamomilla recutita" OR "Camomele" OR "Camomilla" OR "Camomille" OR "Chamomile" OR "Chamomilla" OR "Echte Kamille" OR "Feldkamille" OR "Fleur de Camomile" OR "Kamillen" OR "Kleine Kamille" OR "Manzanilla" OR "M Alemana" OR "Matricaire" OR "OEil du Soleil" OR "Pin Heads" OR "Chamaemelum nobile" OR "Ormenis nobilis" OR "C nobile" OR "A nobilis" OR "O nobilis" OR "Anthemis" OR "A Odorante" OR "A nobilis" OR "Babounge" OR "Babuna Ke Phool" OR "Chamomillae Ramane Flos" OR "Flores Anthemidis" OR "F Anthemidis" OR "Grosse Kamille" OR "Ground Apple" OR "Manzanilla" OR "M Romana" OR "Romische Kamille" OR "Whig Plant" OR "Chanting" OR "Cheirology" OR "Chelated Minerals" OR "Mineral-Amino Acid Complex" OR "Bore Chelate" OR "Chelated Boron" OR "Chelated Cobalt" OR "Chelated Iron" OR "Chelated Molybdenum" OR "Chelated Potassium" OR "Chelated Selenium" OR "Chelated Trace Minerals" OR "Chelated Vanadium" OR "Chelated Zinc" OR "Cobalt Chelate" OR "Cuivre Chelate" OR "Fer Chelate" OR "Minerales Quelados" OR "Mineraux Chelates" OR "Molybdene Chelate" OR "Potassium Chelate" OR "Selenium Chelate" OR "Vanadium Chelate" OR "Zinc Chelate" OR "Chiropractic" OR "Chiro Therap*" OR "Chirotherap*" OR "Chiropraticien" OR "Chiropractie" OR "Chiropractors" OR "Chiropratique" OR "Chiropraxie" OR "Manipulation Rachidienne" OR "Manipulation Vertebrale" OR "Manipulative Therap*" OR "Physical Medicine" OR "Quiropractica" OR "Spinal Manipulative Therap*" OR "Subluxation" OR "Chitosan" OR "Ascorbate de Chitosane" OR "Chitosan-N-Acetylcysteine" OR "Deacetylated Chitin" OR "Enzymatic Polychitosamine Hydrolisat" OR "Poly-D-Glucosamine" OR

"Poly-N-Acetyl-Glucosamine" OR "Quitosano" OR "Sulfated N-Carboxymethylchitosan" OR "Sulfated O-Carboxymethylchitosan" OR "Choline" OR "Trimethylethanolamine" OR "beta-hydroxyethyl" OR "trimethylammonium hydroxide" OR "Colina" OR "Facteur Lipotropique" OR "Hydroxyde de Trimethylammonium" OR "beta-hydroxyethyl" OR "Intrachol" OR "L-Choline" OR "Lipotropic Factor" OR "Methylated Phosphatidylethanolamine" OR "Trimethylethanolamine" OR "Chondroitin Sulfate" OR "Chondroitin 4-sulfate" OR "Chondroitin 4- and 6-sulfate" OR "Chondroitin Polysulfate" OR "Chondroitin Sodium Sulfate" OR "Chondroitin Sulphate" OR "Chondroitine" OR "Chondroitine Sulfate A" OR "Chondroitine Sulfate B" OR "Chondroitine Sulfate C" OR "Chondroitine 4-Sulfate" OR "Chondroitine 4- et 6-Sulfate" OR "Condroitin" OR "Galactosaminoglucuronoglycan Sulfate" OR "Poly-(1->3)-N-Acetyl-2-Amino-2-Deoxy-3-O-Beta-D-Glucopyranurosyl-4-(or 6-)" OR "Polysulfate de Chondroitine" OR "Shark Chondroitin Sulphate" OR "Sulfate de Chondroitine" OR "Sulfate de Galactosaminoglucuronoglycane" OR "Sulfates de Chondroitine" OR "Sulfato de Condroitina" OR "Chromium" OR "Chlorure Chromique" OR "Chrome" OR "Chromic Chloride" OR "Cromo" OR "Glucose Tolerance Factor-Cr" OR "Kali Bichromicum" OR "Numero Atomique 24" OR "Potassium Bichromate" OR "Chromotherap*" OR "Chromotherapeute" OR "Chromotherapie" OR "Chromotherapist" OR "Color Medicine" OR "Color Therap*" OR "Colorologie" OR "Colorology" OR "Cromoterapia" OR "Therapie des Couleurs" OR "Therapie par les Couleurs" OR "Chronotherap*" OR "Cinnamon" OR "C aromaticum" OR "C cassia" OR "Canelero Chino" OR "Canelle" OR "Cannelier Casse" OR "Cannelier de Chine" OR "Casse" OR "Cassia" OR "C Lignea" OR "Chinazimt" OR "Chinesischer Zimtbaum" OR "Cinnamomi Cassiae Cortex" OR "Cinnamomum" OR "Cinnamon" OR "Cinnamoni Cortex" OR "Cinnamonomi Cortex" OR "Cortex Cinnamomi" OR "Gui Zhi" OR "Kassiakanel" OR "Keishi" OR "Laurier des Indes" OR "Nees" OR "Ramulus Cinnamomi" OR "Rou Gui" OR "Sthula Tvak" OR "Taja" OR "Zimbluten" OR "Zimtcassie" OR "C verum" OR "C zeylanicum" OR "Canela" OR "Canelero de Ceilan" OR "Cannelier de Ceylan" OR "Cannelle de Ceylan" OR "Cannelle de Saigon" OR "Cannelle du Sri Lanka" OR "Ceylonzimt" OR "Ceylonzimtbaum" OR "Cinnamon Bark" OR "Dalchini" OR "Tvak" OR "Xi Lan Rou Gui" OR "Zimtbaum" OR "C loureirii" OR "C loureiroi" OR "Nikkei" OR "Nhucgue" OR "Que Thanh" OR "Saigonkanel" OR "Saigonzimt" OR "Saigonzimtbaum" OR "Yukgyenamu" OR "C burmannii" OR "Birmazimt" OR "Birmazimtbaum" OR "Cannelier de Malaisie" OR "Indonesische Kaneel" OR "Indonesischer Zimt" OR "Jaavakaneli" OR "Kayo Manis Padang" OR "Kayu Manis Padang" OR "Korintje" OR "Padangzimt" OR "Padangzimtbaum" OR "C tamala" OR "Chai Gui" OR "Indian Bay Leaf" OR "Indian Bark" OR "Malobathrum" OR "Talish" OR "Pattri" OR "Tamala" OR "Tamalpatra" OR "Tejpat" OR "Tejpata" OR "Tejpatra" OR "Tejpatta" OR "Tez Pat" OR "Tezpat" OR "Cleansing" OR "Climatotherap*" OR "Clinical Ecology" OR "Clove*" OR "Syzygium aromaticum" OR "Caryophylli Flos" OR "Caryophyllum" OR "Clavo de Olor" OR "Ding Xiang" OR "Flores Caryophylli" OR "Gewurznelken Nagelein" OR "Girofle" OR "Giroflier" OR "Kreteks" OR "Lavang" OR "Lavanga" OR "Coenzyme Q10" OR "Ubiquinol" OR "Ubiquinone" OR "Ubidecarenone" OR "Mitoquinone" OR "Coenzima Q-10" OR "Coenzyme Q-10" OR "Ubidecarenone" OR "Ubiquinone-10" OR "Coffee" OR "C arabica" OR "Coffea canephora" OR "C canephora" OR "Coffea robusta" OR "Coffea bukobensis" OR "Coffea liberica" OR "Coffea arnoldiana" OR "C bukobensis" OR "C liberica" OR "C arnoldiana" OR "Cafe" OR "C Robusta" OR "Coffea Cruda" OR "C Cruda" OR "Espresso" OR "Expresso" OR "Java" OR "Mocha" OR "Cognitive Therap*" OR "Coley's Toxin" OR "Mixed Bacterial Vaccine" OR "Argent Colloidal" OR "Argent Ionique" OR "Argent Natif" OR

"Argentum Metallicum" OR "Plata Coloidal" OR "Proteine d'Argent" OR "Silver" OR "Tetrasilver Tetroxide" OR "Tetroxyde de Tetra-Argent" OR "Complementary Medicine" OR "Complementary Therap*" OR "Complementary Approach" OR "Complementary Health" OR "Conjugated Linoleic Acid" OR "cis-9, trans-11 conjugated linoleic acid" OR "Trans-10,cis-12 conjugated linoleic acid" OR "Acide Linoleique Conjugue" OR "Cis-Linoleic Acid" OR "CLA-Triacylglycerol" OR "Linoleic Acid" OR "Contact Reflex Analysis" OR "Copper" OR "Atomic number 29" OR "Cobre" OR "CupricOxide" OR "Cupric Sulfate" OR "Cuprum Aceticum" OR "Cuprum Metallicum" OR "Gluconate de Cuivre" OR "Numero Atomique 29" OR "Oxyde Cuivrique" OR "Pentahydrate de Sulfate de Cuivre" OR "Sulfate de Cuivre" OR "Sulfate Cuivrique" OR "Sulfate Cuprique" OR "Cordyceps" OR "Ophiocordyceps sinensis" OR "O sinensis" OR "C sinensis" OR "Caterpillar Fungus" OR "Champignon Chenille" OR "Chinese Caterpillar Fungus" OR "Dong Chong Xia Cao" OR "Dong Chong Zia Cao" OR "Hsia Ts'Ao Tung Ch'Ung" OR "Jinshuibao Jiaonang" OR "Jinshuibao Pian" OR "Tochukaso" OR "Vegetable Caterpillar" OR "Trametes versicolor" OR "Polyporus versicolor" OR "Boletus versicolor" OR "Polystictus versicolor" OR "Bolet a Couleurs Variees" OR "Bolet Versicolore" OR "Champignon de Queue de Dinde" OR "Coriolus" OR "Kawaratake" OR "Krestin" OR "Polypore a Couleurs Variees" OR "Polypore Versicolor" OR "PolysaccharideK" OR "Polysaccharide Krestin" OR "Polysaccharide Peptide" OR "Polysaccharopeptide" OR "Yun Chi" OR "Yun Zhi" OR "Yunzhi" OR "Yun-Zhi" OR "Counseling" OR "Cranberry" OR "Vaccinium macrocarpon" OR "V macrocarpon" OR "Oxycoccus macrocarpos" OR "O macrocarpos" OR "Vaccinium oxycoccos" OR "V oxycoccos" OR "Oxycoccus hagerupii" OR "Oxycoccus microcarpus" OR "Oxycoccus palustris" OR "Oxycoccus quadripetalus" OR "Vaccinium hagerupii" OR "Vaccinium microcarpum" OR "Vaccinium palustre" OR "O hagerupii" OR "O microcarpus" OR "O palustris" OR "O quadripetalus" OR "V hagerupii" OR "V microcarpum" OR "V palustre" OR "Agrio" OR "Airelle a Gros Fruits" OR "Airelle Europeenne" OR "Airelle Rouge" OR "Arandano" OR "Atoca" OR "Atoka" OR "Bearberry" OR "Canneberge" OR "Craneberry" OR "Da Guo Yue Jie" OR "Da Guo Yue Ju" OR "Da Guo Suan Guo Man Yue Ju" OR "Kliukva" OR "Kranbeere" OR "Man Yue Ju" OR "Man Yue Mei" OR "Moosebeere" OR "Mossberry" OR "Oomi No Tsuruko Kemomo" OR "Petite Cannberge" OR "Pois de Fagne" OR "Pomme des Pres" OR "Ronce d'Amerique" OR "Tsuru-Kokemomo" OR "Craniosacral Therap*" OR "Approche Craniosacrale" OR "Cranial Osteopathy" OR "Cranial Therap*" OR "Cranio-Occipital Technique" OR "Craniosacral" OR "Craniosacrale Therapie" OR "Osteopathie Cranienne" OR "Terapia Craneosacral" OR "Therapie Cranienne" OR "Therapie Craniosacrale" OR "Therapie Cranio-sacree" OR "Therapie Cranio-sacree Upledger" OR "Upledger Therap*" OR "Crystal Therap*" OR "Cristaux" OR "Crystal Healing" OR "Crystals" OR "Electrocrystal Therap*" OR "Gem Therap*" OR "Gemstone Therap*" OR "Guerison par les Pierres" OR "Lithotherapie" OR "Pierre Precieuse" OR "Terapia de Cristal" OR "Therapie par les Cristaux" OR "Therapie par les Pierres" OR "Cumin" OR "Cuminum cyminum" OR "Cuminum odorum" OR "C cyminum" OR "C odorum" OR "Anis Acre" OR "Comino" OR "Cummin" OR "Jeeraka" OR "Svetajiraka" OR "Zira" OR "Cupping" OR "Fire-Cupping" OR "Hijamat" OR "Pneumatic Pulsation Therap*" OR "Shuiguanfa" OR "Ventosaterapia" OR "Ventousotherapie" OR "Wet-Cupping" OR "Curanderismo" OR "Curandera" OR "Curandero" OR "Folk Healers" OR "Latin American Folk Medicine" OR "Latin American Healing" OR "Mexican American Healing Tradition" OR "Current Therap*" OR "Cymatic Therap*" OR "Dance Therap*" OR "Dandelion" OR "T officinale" OR "T vulgare" OR "T dens-leonis" OR "T mongolicum" OR "T sinicum" OR "T laevigatum" OR "Blowball" OR "Cankerwort" OR "Cochet" OR "Couronne de Moine" OR "Delice

Printanier" OR "Dent-de-Lion" OR "Diente de Leon" OR "Dudal" OR "Endive Sauvage" OR "Fausse Chicoree" OR "Florin d'Or" OR "Florion d'Or" OR "Ghasedak" OR "Herba Taraxaci" OR "Laitue de Chien" OR "Lion's Teeth" OR "Lion's Tooth" OR "Piss-A-Bed" OR "Pisse au Lit" OR "Pissenlit" OR "Priest's Crown" OR "Pu Gong Ying" OR "Salade de Taupe" OR "Swine Snout" OR "Taraxacum" OR "Tete de Moine" OR "Wild Endive" OR "Danshen" OR "Salvia bowleyana" OR "Salvia miltiorrhiza" OR "Salvia przewalskii" OR "Salvia yunnanensis" OR "S bowleyana" OR "S miltiorrhiza" OR "S przewalskii" OR "S yunnanensis" OR "Ch'ih Shen" OR "Chinese Sage" OR "Chinese Salvia" OR "Dan Shen" OR "Dan-Shen" OR "Huang Ken" OR "Pin-Ma Ts'ao" OR "Racine de Salvia" OR "Radix Salviae Miltiorrhizae" OR "Radix Salvie Miltiorrhiae" OR "Red Root Sage" OR "Red Rooted Sage" OR "Red Sage" OR "Salvia Przewalskii Mandarinorum" OR "Salvia Root" OR "Sage Miltiorrhiza" OR "Salviae Miltiorrhizae" OR "Sauge Rouge" OR "Shu-Wei Ts'ao" OR "Tan Seng" OR "Tan-Shen" OR "Tzu Tan-Ken" OR "Dehydroepiandrosterone" OR "Dengzhanhua Preparation" OR "Dervish Dance" OR "Sufi Whirling" OR "Sufi Dance" OR "Detoxification" OR "Colon Cleanse" OR "Enema" OR "Desintoxicacion" OR "Detox" OR "Fad Diet" OR "Gallbladder Cleanse" OR "Gallbladder Flushing" OR "Herbal Cleansing" OR "Hydrotherapie du Colon" OR "Jeune de Jus de Fruits" OR "Juice Fasting" OR "Lavement" OR "Liver Cleanse" OR "Liver Flushing" OR "Nettoyage a Base de Plantes" OR "Nettoyage du Colon" OR "Purification Rundown" OR "Starvation Diet" OR "Traitement de Detoxfiication" OR "Water Diet" OR "Water Fasting" OR "Devil's Claw" OR "Harpagophytum procumbens" OR "Uncaria procumbens" OR "Harpagophytum zeyheri" OR "H procumbens" OR "U procumbens" OR "H zeyheri" OR "Devils Claw" OR "Garra del Diablo" OR "Grapple Plant" OR "Griffe du Diable" OR "Harpagophyti Radix" OR "Racine de Griffe du Diable" OR "Racine de Windhoek" OR "Teufelskrallenwurzel" OR "Wood Spider" OR "Devil's Club" OR "Oplopanax horridus" OR "Echinopanax horridus" OR "Fatsia horrida" OR "O horridus" OR "E horridus" OR "F horrida" OR "Bois Piquant" OR "Cukilanarpak" OR "Devils Club" OR "Devil's Root" OR "Fatsia" OR "Garrote del Diablo" OR "Panax Horridum" OR "Diamond Diet" OR "Dianxianning Pill" OR "Diathermy" OR "Diet Therap*" OR "Dihomogammalinolenic Acid" OR "Dimethylaminoethanol" OR "Dimethylethanolamine" OR "Deanol" OR "Dimethylsulfoxide" OR "Dimethyl Sulfoxide" OR "Dimethyl Sulphoxide" OR "Dimethylis Sulfoxidum" OR "Dimethylsulfoxyde" OR "Dimetilsulfoxido" OR "MethylSulphoxide" OR "Sulfoxyde de Dimethyl" OR "Sulphinybismethane" OR "Chelation Therapy" OR "2,3-dimercaptopropane-1-sulfonate" OR "Dimaval" OR "Docosahexaenoic acid" OR "DHA" OR "Acide Docosahexaenoique" OR "Acide Gras d Huile de Poisson" OR "Acide Gras Omega 3" OR "Acide Gras N-3" OR "Acide Gras W-3" OR "Acido Docosahexaenoico" OR "Neuromins" OR "Omega 3" OR "Doman Delacato Patterning Therapy" OR "Dong Quai" OR "Angelica sinensis" OR "Angelica polymorpha var sinensis" OR "Angelica China" OR "Angelicae Gigantis Radix" OR "Angelique Chinoise" OR "Angelique de Chine" OR "Chinese Angelica" OR "Dang Gui" OR "Danggui" OR "Danguia" OR "Don Quai" OR "Kinesisk Kvan" OR "Ligustilides" OR "Phytoestrogen" OR "Radix Angelicae Gigantis" OR "Radix Angelicae Sinensis" OR "Tan Kue Bai Zhi" OR "Tang Kuei" OR "Tanggwi" OR "Drama Therapy" OR "Dream Therapy" OR "Dukun" OR "Ear Candl*" OR "Auricular Candles" OR "Auriculotherapie" OR "Bougie Creuse" OR "Bougie Hopi" OR "Bougie d Oreille" OR "Chandelles Auriculaires" OR "Conage d Oreille" OR "Ear Candle Therapy" OR "Ear Candle Treatment" OR "Ear Coning" OR "Terapia de Vela en el Oido" OR "Therapie Auriculaire Thermale" OR "Thermal-Auricular Therapy" OR "Echinacea" OR "Brauneria angustifolia" OR "Brauneria pallida" OR "Rudbeckia pallida" OR "Brauneria purpurea" OR "Helichroa purpurea" OR "Rudbeckia purpurea" OR "American Cone

Flower" OR "Black Sampson" OR "Black Susans" OR "Comb Flower" OR "Coneflower" OR "Echinaceawurzel" OR "Echinacee" OR "Equinacea" OR "Fleur A Herisson" OR "Hedgehog" OR "Igelkopfwurzel" OR "Indian Head" OR "Kansas Snakeroot" OR "Narrow-Leaved Echinacea" OR "Narrow-Leaved Purple Coneflower" OR "Narrow-leaved Purple Cone Flower" OR "Pale Coneflower" OR "Pale Flower Echinacea" OR "Pale Purple Coneflower" OR "Purple Cone flower" OR "Purpursonnenhutkraut" OR "Purpursonnenhutwurzel" OR "Racine D echininacea" OR "Red Sunflower" OR "Rock-Up-Hat" OR "Roter Sonnenhut" OR "Rudbeckie Pourpre" OR "Schmallblaettrige Kegelblumenwurzel" OR "Schmallblaettriger Sonnenhut" OR "Scurvy Root" OR "Snakeroot" OR "Sonnenhutwurzel" OR "Eclectic Medicine" OR "Edetic Acid" OR "Edetate" OR "Effleurage" OR "Eicosapentaenoic Acid" OR "20:5n-3" OR "Acide Eicosapentaenoique" OR "Acide Ethyle-Eicosapentaenoique" OR "Acide Gras Essentiel" OR "Acide Gras d Huile de Poisson" OR "Acide Gras N-3" OR "Acide Gras Omega 3" OR "Acide Gras Polyinsature" OR "Acide Gras W3" OR "Acido Eicosapentaenoico" OR "Eicosapentanoic Acid" OR "Ethyl Eicosapentaenoic Acid" OR "Ethyl-EPA" OR "Icosapent Ethyl" OR "PUFA" OR "Electrodermal Testing" OR "Vega Test" OR "Wheatstone Bridge" OR "Electrotherapy" OR "Electrostimulation" OR "Elemental Diet" OR "Ellagic Acid" OR "3,4,3',4'-Hydroxyl-Benzopyranol[5,4,3-c,d,e][1]Benzopyrn-6-6'-Dione" OR "Acide Ellagique" OR "Acido Elagico" OR "Gallogen" OR "Emodin" OR "Emotional Freedom Technique" OR "Tapping" OR "Bioenergie" OR "Biofield Therapies" OR "Environmental Medicine" OR "Enzyme Therapy" OR "Ephedra" OR "Alcaloide d Ephedrine" OR "Belcho" OR "Cao Mahuang" OR "Chinese Joint-Fir" OR "Cao Ma-Huang" OR "Desert Herb" OR "Efedra" OR "Ephedre" OR "Ephedrine" OR "Epitonin" OR "Herbal Ecstasy" OR "Indian Jointfir" OR "Joint Fir" OR "Ma Huang" OR "Mahuanggen (Ma Huang Root)" OR "Popotillo" OR "Raisin de Mer" OR "Sea Grape" OR "Teamsters Tea" OR "The de Desert" OR "Yellow Astringent" OR "Yellow Horse" OR "Zhong Mahuang" OR "Esoteric Therapy" OR "Essaic" OR "Essence Therapy" OR "Estrogen Supplement" OR "Ethnomedicine" OR "Ethylenediaminetetraacetic Acid" OR "Evening Primrose" OR "Oenothera biennis" OR "Oenothera muricata" OR "Oenothera rubricaulis" OR "Oenothera suaveolens" OR "Onagra biennis" OR "Aceite de Onagra" OR "Acide Cis-linoleique" OR "Cis-Linoleic Acid" OR "Evening Star" OR "Fever Plant" OR "Herbe-aux-anes" OR "Huile de Graines d Onagre" OR "Huile D Onagre" OR "Huile de Primerose" OR "Huile de Primevere Vesperale" OR "Jambon de Jardinier" OR "Jambon du Paysan" OR "Kings Cureall" OR "Mache Rouge" OR "Night Willow-Herb" OR "OEnothere" OR "Onagraire" OR "Onagre Bisannuelle" OR "Onagre Commune" OR "Primevere du Soir" OR "Primrose" OR "Scabish" OR "Scurvish" OR "Sun Drop" OR "Exercise" OR "Physical Therapy" OR "Experience Based Medicine" OR "Eye Movement Desensitization and Reprocessing" OR "Fasting" OR "Caloric Restriction" OR "Energy Restriction" OR "Intermittent Energy Restriction" OR "Intermittent Severe Energy Restriction" OR "Time-Restricted Feeding" OR "Total Caloric Desistance" OR "Zero Calorie Diet" OR "Fat-Restricted Diet" OR (Fatty acid*) OR "Acides Gras Cetylated" OR "Acides Gras Cetyles" OR "Acides Gras Esterises" OR "Acides Gras Mono-Insatures Cetyles" OR "Acidos Grasos Cetilados" OR "Cerasomal-cis-9-cetylmyristoleate" OR "Cetyl Decanoate" OR "Cetyl Laurate" OR "Cetyl Laureate" OR "Cetyl Myristate" OR "Cetyl Myristoleate" OR "Cetyl Oleate" OR "Cetyl Palmitate" OR "Cetyl Palmitoleate" OR "Cetyl Stearate" OR "Cetylmyristoleate" OR "Cis-9-cetylmyristoleate" OR "Laureate Cetyl" OR "Myristate Cetyl" OR "Myristoleate Cetyl" OR "Oleate Cetyl" OR "Palmitate Cetyl" OR "Palmitoleate Cetyl" OR "Acides Gras Essentiels N-6" OR "Acides Gras Omega-6" OR "Acides Gras Polyinsatures" OR "Acidos Grasos Omega 6" OR "Huiles d Omega 6" OR "Omega 6 Oils" OR "Feldenkrais Method" OR

"Awareness Through Movement" OR "Feldenkrais Bodywork" OR "Functional Integration" OR "Gestalt Synergy" OR "Feng Shui" OR "Fengshui" OR "Foong Shway" OR "Fung Shway" OR "Fusui" OR "Phong-Thuy" OR "Pung-Su" OR "Fenugreek" OR "Trigonella foenum-graecum" OR "Alholva" OR "Bird's Foot" OR "Bockshornklee" OR "Bockshornsame" OR "Chandrika" OR "Fenogreco" OR "Fenugrec" OR "Foenugraeci Semen" OR "Foenugreek" OR "Greek Clover" OR "Greek Hay" OR "Greek Hay Seed" OR "Hu Lu Ba" OR "Methi" OR "Methika" OR "Medhika" OR "Senegrain" OR "Senegre" OR "Trigonella" OR "Trigonelle" OR "Woo Lu Bar" OR "Feverfew" OR "Tanacetum parthenium" OR "Chrysanthemum parthenium" OR "Chrysanthemum praealtum" OR "Leucanthemum parthenium" OR "Matricaria eximia" OR "Matricaria parthenium" OR "Pyrethrum parthenium" OR "Altamisa" OR "Bachelor's Buttons" OR "Chrysantheme Matricaire" OR "Featerfoiul" OR "Featherfew" OR "Featherfoil" OR "Flirtwort Midsummer Daisy" OR "Grande Camomille" OR "Matricaria" OR "Partenelle" OR "Pyrethre Dore" OR "Pyrethre Mousse" OR "Santa Maria" OR "Tanaceti Parthenii" OR "Tanaisie Commune" OR "Finnish Sauna" OR "Fish Oil" OR "Aceite de Pescado" OR "Acides Gras Omega 3" OR "Acides Gras N-3" OR "Acides Gras Polyinsatures N-3" OR "Acides Gras W-3" OR "Ethyl Ester" OR "Ester Ethylique de l'AEP" OR "Ester Ethylique de l'ADH" OR "Herring Oil" OR "Huile de Foie de Morue" OR "Huile de Hareng" OR "Huile de Menhaden" OR "Huile de Poisson" OR "Huile de Saumon" OR "Huile de Thon" OR "Huile Lipidique Marine" OR "Huile Marine*" OR "Lipides Marins" OR "Marine Lipid Concentrate" OR "Marine Lipids" OR "Marine Oil" OR "Marine Fish Oil" OR "Marine Lipid Oil" OR "Marine Triglyceride" OR "Menhaden Oil" OR "Salmon Oil" OR "Triglycerides Marins" OR "Tuna Oil" OR "Tuna Fish Oil" OR "Chrysin*" OR "5,7-dihydroxy-2-phenyl-4H-chromen-4-one" OR "5,7-Chrysin, 5,7-Dihydroxyavone" OR "Flavone X" OR "Flavonoid*" OR "Galangin*" OR "Flavanone" OR "Diosmin*" OR "3', 5, 7-trihydroxy-4'-methoxyavone-7-rhamnoglucoside" OR "Bioavonoid*" OR "Bioavonoid Complex" OR "Bioavonoid Concentrate" OR "Bioavonoid Extract" OR "Bioavonode* d Agrume*" OR "Citrus Bioavones" OR "Citrus Bioavonoid*" OR "Citrus Bioavonoid Extract" OR "Citrus Flavones" OR "Citrus Flavonoids" OR "Complexe de Bioavonoide*" OR "Concentre de Bioavonoide*" OR "Diosmetin 7-O-rutinoside" OR "Extrait de Bioavonoide*" OR "Micronised Puried Flavonoid Fraction" OR "Quercetin*" OR "3,3',4'5,7-Pentahydroxyavone" OR "Bioavonoide* de Citron" OR "Flavones de Citron" OR "Meletin*" OR "Quercetin Dihydrate" OR "Sophretin*" OR "Hesperidin*" OR "Hesperidin Methyl Chalcone" OR "Trimethylhesperidin-Chalcon" OR "Rutin*" OR "3, 3', 4', 5, 7-pentahydroxyavone-3-rhamnoglucoside" OR "Eldrin" OR "Flavonoides d Agrumes" OR "Quercetin-3-Rhamnoglucoside" OR "Quercetin*-3-Rutinoside" OR "Rutosid*" OR "Sclerutin" OR "Sophorin" OR "Flaxseed" OR "Linum usitatissimum" OR "Linum crepitans" OR "Linum humile" OR "Alasi" OR "Aliviraaii" OR "Echter Lein" OR "Flachs" OR "Flax" OR "Flachssamen" OR "Graine de Lin" OR "Kattan" OR "Keten" OR "Leinsamen" OR "Lignanes de Lin" OR "Lignans" OR "Linaza" OR "Lini Semen" OR "Linho" OR "Lino" OR "Linseed" OR "Lint Bells" OR "Linum" OR "Malsag" OR "Phyto-Oestrogene" OR "Saatlein" OR "Tisii" OR "Winterlien" OR "Flor Essence Formula" OR "Flower Essence" OR "Flower Remedies" OR "Folk Medicine" OR "Frangula purshiana" OR "Free and Easy Wanderer" OR "Fringe Medicine" OR "Fructans" OR "Fructooligosaccharides" OR "Chicory Inulin Hydrolysate" OR "Complexe d'Oligosaccharide" OR "Fructo Oligo Saccharides" OR "Fructo-Oligosacaridos" OR "Inulin Hydrolysate" OR "Oligofructan" OR "Oligofructose" OR "Oligosaccharides" OR "Prebiotic" OR "Prebiotique" OR "Short Chain Fructo-Oligosaccharides" OR "Fu Zheng" OR "Fuzheng" OR "Galactans" OR "Galacto-Oligosaccharides" OR

"Galactooligosaccharides" OR "Gamma Oryzanol" OR "Gama Orizanol" OR "Gamma-OZ" OR "Oryzanol" OR "Gamma-Linolenic Acid" OR "Acide Gammalinolenique" OR "Acide Gamma-Linolenique" OR "Acido Gama Linolenico" OR "Gammalinolenic Acid" OR "Gamolenic Acid" OR "(Z,Z,Z)-Octadeca-6,9,12-Trienoic Acid" OR "Garcinia" OR "Cambogia binucao" OR "Cambogia gemmi-guta" OR "Cambogia solitaria" OR "Garcinia affinis" OR "Garcinia cambogia" OR "Garcinia sulcata" OR "Mangostana cambogia" OR "Brindal Berry" OR "Brindle Berry" OR "Gorikapuli" OR "Kankusta" OR "Kudam Puli" OR "Malabar Tamarind" OR "Tamarinier de Malabar" OR "Vrikshamla" OR "Garlic" OR "Allium sativum" OR "Ajo" OR "Alho" OR "Allii Sativi Bulbus" OR "Allium" OR "Angio D India" OR "Camphor Of The Poor" OR "Echte Rokkenbolle" OR "Knoblauch" OR "Lahsun" OR "Lasun*" OR "Maneul" OR "Nectar Of The Gods" OR "Ninniku" OR "Poor Man's Treacle" OR "Rason" OR "Rocambole" OR "Rockenbolle" OR "Rust Treacle" OR "Schlangenknoblauch" OR "Stinking Rose" OR "Suan" OR "Thoum" OR "Vitlok" OR "Genistein Combined Polysaccharide" OR "Basidiomycetes Polysaccharide" OR "Fermented Genistein" OR "Fermented Isoflavone" OR "Genistein Polysaccharide" OR "Genisteine du Polysaccharide Combine" OR "Genisteine Fermentee" OR "Isoflavone Combined Polysaccharide" OR "Isoflavone Fermentee" OR "Polisacaridos Combinados de Genisteina" OR "Polysaccharide de Genisteine" OR "Polysaccharide des Basidiomycetes" OR "Polysaccharide d Isoflavone de Soja" OR "Soy Isoflavone Polysaccharide" OR "Germander" OR "Teucrium chamaedrys" OR "Camedrio" OR "Chasse-Fievre" OR "Cheneau" OR "Chenette" OR "Germandree" OR "Petit Chene" OR "Teucrium scordium" OR "Chamarras" OR "Escordio" OR "Germandrina de Agua" OR "Gerson Therapy" OR "Gerson Diet" OR "Gestalt Therapy" OR "Ginger" OR "Zingiber officinale" OR "Amomum zingiber" OR "Ardraka" OR "Gan Jiang" OR "Gingembre" OR "Huile Essentielle de Gingembre" OR "Imber" OR "Jengibre" OR "Jiang" OR "Kankyo" OR "Kanshokyo" OR "Nagara" OR "Rhizoma Zingiberi*" OR "Rhizoma Zingiberis Recens" OR "Shen Jiang" OR "Sheng Jiang" OR "Shoga" OR "Shokyo" OR "Shunthi" OR "Srungavera" OR "Sunth" OR "Sunthi" OR "Vishvabheshaja" OR "Zinzeberis" OR "Zinziber Officinale" OR "Zinziber Officinalis" OR "Ginkgo" OR "Abricot Argente Japonais" OR "Adiantifolia" OR "Arbe aux Ecus" OR "Arbe aux Quarante Ecus" OR "Arbe du Ciel" OR "Arbre Fossile" OR "Bai Guo Ye" OR "Baiguo" OR "Fossil Tree" OR "Japanese Silver Apricot" OR "Kew Tree" OR "Maidenhair Tree" OR "Noyer du Japon" OR "Pei Go Su Ye" OR "Salisburia Adiantifolia" OR "Yen Xing" OR "Yinhsing" OR "Ginseng" OR "Panax schinseng" OR "Guigai" OR "Hong Shen" OR "Insam" OR "Jen-Shen" OR "Jinsao" OR "Jintsam" OR "Korean Panax" OR "Mandragore de Chine" OR "Ninjin" OR "Ninzin" OR "Panax Coreen" OR "Racine de Vie" OR "Renshen" OR "Renxian" OR "Sheng Shai Shen" OR "Panax quinquefolius" OR "Baie Rouge" OR "Panax Quinquefoli*" OR "Red Berry" OR "Ren Shen" OR "Shang" OR "Shi Yang Seng" OR "Xi Yang Shen" OR "Glucosamine" OR "3-Amino-6-(Hydroxymethyl)Oxane-2,4,5-Triol Sulfate" OR "(3R,4R,5S,6R)-3-Amino-6-(Hydroxymethyl)Oxane-2,4,5-Triol Hydrochloride" OR "2-Acetamido-2-deoxyglucose" OR "2-Amino-2-Deoxy-Beta-D-Glucopyranose Hydrochloride" OR "2-Amino-2-Deoxy-D-Glucosehydrochloride" OR "2-Amino-2-Deoxy-Beta-D-Glucopyranose" OR "2-Amino-2-Deoxy-D-Glucose Sulfate" OR "Acetylglucosamine" OR "GlcNAc" OR "Chitosamine" OR "Chlorhidrato de Glucosamina" OR "Chlorhydrate de Glucosamine" OR "D-Glucosamine HCl" OR "D-Glucosamine Hydrochloride" OR "D-Glucosamine Sulfate" OR "D-Glucosamine Sulphate" OR "Mono-Sulfated Saccharide" OR "NAcetil Glucosamina" OR "N-Acetylglucosamine" OR "N-Acetyl D-Glucosamine" OR "Poly-N-Acetyl Glucosamine" OR "Poly-NAG" OR "Poly-(1-3)-N-Acetyl-2-Amino-2-Deoxy-3-OBeta-D-Glucopyranurosyl-4-(or 6-) Sul" OR "Saccharide

Mono-Sulfate" OR "Saccharide Sulfate" OR "Sulfate de Glucosamine" OR "Sulfated Monosaccharide" OR "Sulfated Saccharide" OR "Sulfato de Glucosamina" OR "Glutamine" OR "L-(+)-2-Aminoglutaramic acid" OR "Acide Glutamique" OR "Acide L-(+)-2-Aminoglutaramique" OR "Acide L-Glutamique" OR "Alanyl-L-Glutamine Dipeptide" OR "Glutamate" OR "Glutamic Acid" OR "Glutamina" OR "Glutaminate" OR "Levoglutamide" OR "Levoglutamine" OR "L-Alanyl-L-Glutamine" OR "L-Glutamic Acid" OR "L-Glutamine" OR "N-Acetyl-L-Glutamine" OR "(S)-2,5-Diamino-5-oxopentanoic Acid" OR "Gluten-Free Diet" OR "Wheat-Free Diet" OR "Glyconutrients" OR "Ambrotose" OR "Gluconutrientes" OR "Glyconutriments" OR "Manapol" OR "Grahamism" OR "Vitis vinifera" OR "Vitis labrusca" OR "Activin" OR "Calzin" OR "Draksha" OR "Enocianina" OR "Raisins" OR "Feuille de Vigne Rouge" OR "Flame Seedless" OR "Folia Vitis Viniferae" OR "Grapes" OR "Grapeseed" OR "Kali Draksha" OR "Leucoanthocyanin" OR "Muscat" OR "Muskat" OR "Oligomeres Procyanidoliques" OR "Oligomeric Proanthocyanidins" OR "Oligomeric Procyanidins" OR "Petite Sirah" OR "Proanthocyanidines Oligomeriques" OR "Proanthodyn*" OR "Procyanidines Oligomeriques" OR "Procyanidolic Oligomers" OR "Raisin" OR "Red Globe" OR "Red Malaga" OR "Red Vine" OR "Sultanas" OR "Thompson Seedless" OR "Vin Rouge" OR "Gravity Inversion" OR "Inversion Therapy" OR "Greater Celandine" OR "Chelidonium majus" OR "Bai Qu Cai" OR "Celandine" OR "Celidonia Mayor" OR "Chelidonii Herba" OR "Grande Chelidoine" OR "Grande Eclaire" OR "Herbe a Verrues" OR "Herbe aux Verrues" OR "Parties Aeriennes de la Grande Chelidoine" OR "Racine de Chelidoine" OR "Rhizome de Chelidoine" OR "Schollkraut" OR "Swallow Wort" OR "Tetterwort" OR "Verruguera" OR "Green Tea" OR "Camellia sinensis" OR "Camellia thea" OR "Camellia theifera" OR "Thea bohea" OR "Thea sinensis" OR "Thea viridis" OR "Benifuuki" OR "Constituant Polyphenolique de The Vert" OR "Epigallo Catechin Gallate" OR "Epigallo-Catechine Gallate" OR "Epigallocatechin Gallate" OR "Extrait de The Vert" OR "Kunecatechins" OR "Poly E" OR "Polyphenon E" OR "Te Verde" OR "Tea Extract" OR "Tea" OR "Yabukita" OR "Group Therapy" OR "Group Support" OR "Support Groups" OR "Social Support" OR "Gso-Ba Rig-Pa" OR "Guarana" OR "Paullinia cupana" OR "Paullinia sorbilis" OR "Brazilian Cocoa" OR "Cacao Bresilien" OR "Guarana Seed Extract" OR "Guaranine" OR "Guggul" OR "Commiphora wightii" OR "Commiphora mukul" OR "Balsamodendrum wightii" OR "Balsamodendrum mukul" OR "Devadhupa" OR "Gomme Guggul" OR "Gomme-Resine de Guggul" OR "Guggal" OR "Guggulipid*" OR "Guggulsterone*" OR "Guggulu" OR "Guglipid" OR "Gugulipid" OR "Gum Guggal" OR "Indian Bdellium" OR "Indian Guggulipids" OR "Koushika" OR "Mukul Myrrh Tree" OR "Palankasha" OR "Imagerie Guidee" OR "Imagery" OR "Imaginacion Guiada" OR "Visualisation" OR "Visualization" OR "Guiling Pa'an Wan" OR "Gurah" OR "Hair Analysis" OR "Hands-On Healing" OR "Hawthorn" OR "Mespilus laevigata" OR "Aubepine" OR "Bianco Spino" OR "Bois de Mai" OR "Cenellier" OR "Crataegi Flos" OR "Crataegi Folium" OR "Crataegi Fructus" OR "Crataegus" OR "Epine Blanche" OR "Epine de Mai" OR "Espino Blanco" OR "Fructus Crataegi" OR "Haagdorn" OR "Hagedorn" OR "Harthorne" OR "Hawthorne" OR "Hedgethorn" OR "Maybush" OR "Maythorn" OR "Mehlbeebaum" OR "Meidorn" OR "Noble Epine" OR "Shen Zha" OR "Oneseed Hawthorn" OR "Poire d'Oiseaux" OR "Sable Epine" OR "Shanzha" OR "Weissdorn" OR "Whitehorn" OR "Heliotherapy" OR "Heliotrope" OR "Hellerwork" OR "Deep Tissue Bodywork and Movement Education" OR "Structural Integration" OR "Helminth Therapy" OR "Trichuris suis ova" OR "Trichuris trichiura ova" OR "Tisane" OR "High Fiber Diet" OR "Hirudotherapy" OR "Hirudin" OR "Hirudoterapia" OR "Hirudotherapie" OR "Leech" OR "Leeches" OR "Sangsue Anticoagulante" OR "Sangsue Medicinale" OR "Sangsue Officinale" OR "Therapie

par les Sangsues" OR "Holistic Health" OR "Holism" OR "Holographic Resonance Repatterning" OR "Home Remedies" OR "Homeopathy" OR "Homeopathic" OR "Homeopathie" OR "Homeopathique" OR "Homeopatia" OR "Homeoprophylaxis" OR "Maladie Semblable" OR "Medecine Homeopathique" OR "Nosodes" OR "Remede Homeopathique" OR "Similar Disease" OR "Homoharringtonine" OR "Homotoxicology" OR "Hoodia" OR "Cactus" OR "Kalahari Diet" OR "Xhoba" OR "Humulus lupulus" OR "Asperge Sauvage" OR "Couleuvree" OR "Hopfenzapfen" OR "Houblon" OR "Lupuli Strobulus" OR "Lupulin" OR "Lupulo" OR "Pi Jiu Hua" OR "Salsepareille Indigene" OR "Vigne du Nord" OR "Aesculus hippocastanum" OR "Aescin" OR "Buckeye" OR "Castano de Indias" OR "Chataignier de Mer" OR "Chataignier des Chevaux" OR "Chestnut" OR "Conker Tree" OR "Escine" OR "Faux-Chataignier" OR "Hippocastani Cortex" OR "Hippocastani Flos" OR "Hippocastani Folium" OR "Hippocastani Semen" OR "Hippocastanum Vulgare Gaertn" OR "Marron Europeen" OR "Marronnier" OR "Venastat" OR "Venostat" OR "Venostasin Retard" OR "Horticultural Therapy" OR "Hoxsey" OR "Humor Therapy" OR "Huna" OR "Huperzine" OR "Huperzina A" OR "Selagine" OR "Hydrazine Sulfate" OR "Segidrin" OR "Sehydrin" OR "Sulfate d'Hydrazine" OR "Sulfato de Hidracina" OR "Hydrogen Peroxide" OR "Hydrotherapy" OR "Hot and Cold Water Treatment" OR "Watsu" OR "Hypnotherapy" OR "Altered States Of Consciousness" OR "Autogenic Training" OR "Hypnoanalgesia" OR "Hypnobirthing" OR "Hypnosis" OR "Mesmerism" OR "Post-Hypnotic Suggestion" OR "Immune Boosters" OR "Immunoaugmentative Therapy" OR "Immuno-Augmentative Therapy" OR "Immunoaugmentation" OR "Initiatory Medicine" OR "Inositol" OR "Hexahydroxycyclohexane" OR "1,2,3,4,5,6-Cyclohexanehexol, cis-1,2,3,5-trans-4,6-Cyclohexanehexol" OR "D-chiro-inositol" OR "(+)-chiroinositol" OR (1,2,5,3,4,6-inositol) OR "(1S)-inositol" OR "(1S)-1,2,4,3,5,6-inositol" OR "Antialopecia Factor" OR "Cyclohexito" OR "Dambrose" OR "D-Myo-Inositol" OR "Facteur Anti-Alopecique" OR "Inose" OR "Inosite" OR "Inositol Monophosphate" OR "Lipositol" OR "Meso-Inositol" OR "Monophosphate d'Inositol" OR "Mouse Antialopecia Factor" OR "Myo-Inositol" OR "Vitamin* B8" OR "Integrative Medicine" OR "Integrated Medicine" OR "Integrative Therapy" OR "Integrated Therapy" OR "Integrative Approach" OR "Integrated Approach" OR "Integrative Health" OR "Integrated Health" OR "Interactive Metronome" OR "Iodine" OR "Atomic number 53" OR "Iode" OR "Iodide" OR "Iodized Salt" OR "Iodure" OR "Lugol's Solution" OR "Numero Atomique 53" OR "Periodate de Sodium" OR "Potassium Triiodide" OR "Sodium Periodate" OR "Solution de Lugol" OR "Yodo" OR "Ipriflavone" OR "7-isopropoxyisoflavone" OR "7-Isopropoxy Isoflavone" OR "Ipriflavona" OR "Iridology" OR "Bilan Iridologique" OR "Diagnostic par l'Iris" OR "Irido-Diagnostique" OR "Iridiologie" OR "Iridologia" OR "Iridologist" OR "Iridologue" OR "Iris" OR "Iron" OR "Atomic number 26" OR "Elemental Iron" OR "Ferric Iron" OR "Ferric Hydroxide Polymaltose" OR "Ferric Orthophosphate" OR "Ferric Oxide Saccharide" OR "Ferric Sodium Citrate" OR "Ferrous Carbonate Anhydrous" OR "Ferrous Citrate" OR "Ferrous Fumarate" OR "Ferrous Gluconate" OR "Ferrous Iron" OR "Ferrous Pyrophosphate" OR "Ferrous Succinate" OR "Ferrous Sulfate" OR "Ferrum Phosphoricum" OR "Hierro" OR "Orthophosphate Ferrique" OR "Numero Atomique 26" OR "Islamic Medicine" OR "Isoflavones" OR "Jaffe Mellor Techniques" OR "Jamu" OR "Jasmine" OR "Jasminum grandiflorum" OR "Jasminum officinale" OR "Jasmin" OR "Jasmind'Espagne" OR "Jati" OR "Jazmin" OR "Poet's Jessamine" OR "Jin Bu Huan" OR "Jin Li Da Liquor" OR "Jin Shin" OR "Juice Fasting" OR "Juice Cleanse" OR "Juice Detox" OR "Juice Therapy" OR "Juicing" OR "Kampo Medicine" OR "Japanese Medicine" OR "Medecine Japonaise" OR "Medecine Kampo" OR "Medecine Orientale" OR "Medecine Traditionnelle Asiatique" OR "Medicina Kanpo" OR

"Medecine Kampo" OR "Oriental Medicine" OR "Traditional Asian Medicine" OR "Kava" OR "Piper methysticum" OR "Ava Pepper" OR "Ava Root" OR "Awa" OR "Gea" OR "Intoxicating Pepper" OR "Kao" OR "Kavain" OR "Kavapipar" OR "Kawa" OR "Kawapfeffer" OR "Kew" OR "Lawena" OR "Long Pepper" OR "Malohu" OR "Maluk" OR "Meruk" OR "Milik" OR "Poivre des Cannibales" OR "Poivre des Papous" OR "Rauschpfeffer" OR "Sakau" OR "Tonga" OR "Waka" OR "Wurzelstock" OR "Yagona" OR "Yangona" OR "Yaqona" OR "Yaquon" OR "Yongona" OR "Ketogenic Diet" OR "Low-Carbohydrate Diet" OR "Low Glycemic Index Treatment" OR "Medium Chain Triglyceride Diet" OR "Modied Atkin's Diet" OR "Kinesiology Services" OR "Kirlian Photography" OR "Aura Photography" OR "Coronal Discharge Photography" OR "Fingertip Aura" OR "Gas Discharge Visualization" OR "Kirlian Diagnostics" OR "Kirlian Electrophotography" OR "Kirlian-Graphic" OR "Spark Electrography" OR "Tesla Coil Kirlian Photography" OR "Kneipp Cure" OR "Kombucha" OR "Algue de The" OR "Champagne of Life" OR "Champignon de la Charite" OR "Champignon des Heros" OR "Champignon de Longue Vie" OR "Champignon Miracle" OR "Combucha Tea" OR "Fungus Japonicus" OR "Kwassan" OR "Manchurian Fungus" OR "Mushroom Infusion" OR "Petite Mere Japonaise" OR "Spumonto" OR "T'Chai from the Sea" OR "Tschambucco" OR "Krebiozen" OR "Carcalon" OR "Drug X" OR "Substance X" OR "La'au Lapa'au" OR "Laetrile" OR "Amygdalin" OR "Lam Kam San Heklin" OR "2-Amino-5-guanidinopentanoic acid" OR "2-Amino-5-(diaminomethylidene amino) pentanoic acid" OR "(2S)-2-Amino-5-{[amino (imino) methyl]amino}pentanoic Acid" OR "(S)-2-Amino-5- Guanidinopentanoic Acid" OR (Acide 2-Amino-5-Guanidinopentanoique) OR "Arginine" OR "L-Arginina" OR "RGene 10" OR "Laser Therapy" OR "Laughter Therapy" OR "Lavender" OR "Lavandula angustifolia" OR "Lavandula officinalis" OR "Lavandula vera" OR "Lavandula spica" OR "Lavandula dentata" OR "Lavandula latifolia" OR "Lavandula pubescens" OR "Lavandula stoechas" OR "Alhucema" OR "Huile Essentielle de Lavande" OR "Lavanda" OR "Lavande" OR "Ostokhoddous" OR "Leafflower" OR "Phyllanthus" OR "Lecithin*" OR "Ovolecithin*" OR "Phospholipide de Soja" OR "Phospholipide* de Soya" OR "Soy Phospholipid*" OR "Vegilecithin" OR "Vitellin*" OR "Balm" OR "Melissa officinalis" OR "Balsamo de Limon" OR "Cure-All" OR "Dropsy Plant" OR "Honey Plant" OR "Melisa" OR "Melissa" OR "Melissae Folium" OR "Melisse" OR "Melissenblatt" OR "Monarde" OR "Sweet Mary" OR "Toronjil" OR "Lentinan" OR "Lentinane" OR "Lentinus Edodes" OR "Polysaccharide" OR "Xiangguduotang" OR "Xiangguduotangzhusheye" OR "Licorice" OR "G echinate" OR "G glabra" OR "G glandulifera" OR "G uralensis" OR "Acide Glycyrrhizique" OR "Acide Glycyrrhizinique" OR "Alcacuz" OR "Alcazuz" OR "Bois Doux" OR "Bois Sucre" OR "Can Cao" OR "Gan Cao" OR "Gan Zao" OR "Glabra" OR "Glycyrrhiza" OR "Glycyrrhizae" OR "Glycyrrhizic Acid" OR "Glycyrrhizinic Acid" OR "Isoavone" OR "Jethi-Madh" OR "Kanzo" OR "Lakritze" OR "Liquiritiae Radix" OR "Liquirizia" OR "Liquorice" OR "Mulathi" OR "Mulethi" OR "Orozuz" OR "Phytoestrogen" OR "Phyto-oestrogene" OR "Racine Douce" OR "Regalissse" OR "Regaliz" OR "Reglisse" OR "Regliz" OR "Subholz" OR "Sussholz" OR "Sweet Root" OR "Yashtimadhu" OR "Yashti-Madhu" OR "YashtiMadhuka" OR "Zhi Gan Cao" OR "Lifestyle Changes" OR "Light Therap*" OR "Acu-Light Therap*" OR "Balneophototherap*" OR "Bath PUVA" OR "Bright Light Therap*" OR "Dead Sea Climatotherap*" OR "Esogetic Colorpuncture" OR "Fiber-Optic Phototherap*" OR "Irradiation" OR "Light Treatment" OR "Light-Emitting Diode" OR "Phototherap*" OR "PDT Photo Therap*" OR "Photobiology" OR "Photochemotherap*" OR "Photodynamic Therap*" OR "Photomedicine" OR "Photophoresis" OR "Photosensitizers" OR "Ultraviolet therap*" OR "Anodyne Therap*" OR "Monochromatic Infrared Photo Energy" OR

"Lime" OR "Citrus aurantifolia" OR "C aurantifolia" OR "Citrus medica var acida" OR "Citrus acida" OR "Citrus limetta var aromatica" OR "Limonia aurantifolia" OR "Dam's Apple" OR "Bara Nimbu" OR "Bijapura" OR "Citron Vert" OR "Citronnier Vert" OR "Lima" OR "Limette" OR "Limetta" OR "Limettier" OR "Turanj" OR "L-Isoleucine" OR "Liuwei Dihuang Pills" OR "Live Cell Therap*" OR "Livingston Wheeler Therap*" OR "L-Leucine" OR "Low Carbohydrate Diet" OR "Low Fat Diet" OR "Low Glycemic Index Diet" OR "Low GI Diet" OR "Low Protein Diet" OR "L-Valine" OR "Lymph Therap*" OR "Lysine" OR "L-2,6-diaminohexanoic acid" OR "Hydrochlorure de L-Lysine" OR "Lisina" OR "L-Lysine" OR "L-Lysine HCl" OR "L-Lysine Hydrochloride" OR "L-Lysine Monohydrochloride" OR "Macrobiotic Diet" OR "Ma-Pi 2 Diet" OR "Macrobiotics" OR "Macrobiotism" OR "Maggot therap*" OR "Biodebridement" OR "Biosurg*" OR "Biotherap*" OR "Larva* therap*" OR "Larval Debridement Therap*" OR "Maggot Debridement therap*" OR "Magnesium" OR "Dimagnesium Malate" OR "Epsom Salts" OR "Magnesia" OR "Magnesio" OR "Numero Atomique 12" OR "Sels d'Epsom" OR "Magnet therap*" OR "Aimant" OR "Biomagnetism*" OR "Bracelet Magnetique" OR "Collier Magnetique" OR "Electromagnetic therap*" OR "Magnet*" OR "Pulsed Electromagnetic Field Therap*" OR "Therapie par Champ Electromagnetique" OR "Therapie par Champ Electromagnetique Pulse" OR "Therapie Electromagnetique" OR "Therapie Magnetique" OR "Maharishi Amrit Kalish" OR "Lalish Method" OR "Maintenance therap*" OR "G frondose" OR "Champignon Dansant" OR "Champignon des Fous Dansants" OR "Grifola" OR "Hen of the Woods" OR "Maitake" OR "Monkey's Bench" OR "Ram's Head" OR "Roi des Champignons" OR "Sheep's Head" OR "Shelf Fungi" OR "Manganese" OR "Atomic Number 25" OR "Manganeso" OR "Manganum" OR "Sulfate de Manganes" OR "Mantras" OR "Manual Lymphatic Drainage" OR "Marma therap*" OR "Marshmallow" OR "Althaea officinalis" OR "A officinalis" OR "Althaea taurinensis" OR "Altea" OR "Alteia" OR "Althaeae Folium" OR "Althaeae Radi" OR "Althea" OR "Althee" OR "Guimauve" OR "Gulkhairo" OR "Herba Malvae" OR "Mallards" OR "Malvavisco" OR "Marsh Maillo" OR "Mauve Blanche" OR "Mortification Root" OR "Sweet Weed" OR "Wymote" OR "Massage" OR "Bindegewebsmassage" OR "Connective Tissue Manipulation" OR "Myofascial Release" OR "Petrissage" OR "Shiatsu" OR "Tui Na" OR "Medical Hydrology" OR "Medicinal Fungi" OR "Meditation" OR "Meditacion" OR "Mindfulness" OR "Yoga" OR "Asana" OR "Pranayama" OR "Kundaliniyoga" OR "Surya Namaskara" OR "Viniyoga" OR "Yogasana" OR "Yogic Training" OR "Yogoda" OR "Mediterranean Diet" OR "Diet Mediterranean" OR "Mediterranean Eating Pattern" OR "Mediterranean-Style Diet" OR "Medium Chain Triglycerides" OR "1,2,3-Propanetriol Trioctanoate" OR "Acide Caprique" OR "Acide Caprylique" OR "Acide Caproique" OR "Acide Laurique" OR "Capric Acid" OR "Caproic Acid" OR "Caprylic Acid" OR "Caprylic Triglycerides" OR "Laurate-Rich MCTs" OR "Lauric Acid" OR "Medium-Chain Triacylglycerols" OR "Medium-Chain Triglycerides" OR "Triacylglycerols a Chaine Moyenne" OR "Tricaprylin" OR "Triglycerides Capryliques" OR "Trigliceridos de Cadena Media" OR "Trioctanoin" OR "Megavitamins" OR "Melatonin*" OR "N-Acetyl-5-Methoxytryptamine" OR "5-Methoxy-N-Acetyltryptamine" OR "Pineal Hormone" OR "Mental Healing" OR "Meridian System" OR "Mesotherap*" OR "Metamorphic Technique" OR "Micronutrients" OR "Milk Thistle" OR "Silybum marianum" OR "S marianum" OR "Carduus marianus" OR "Artichaut Sauvage" OR "Cardo Lechoso" OR "Cardui Mariae Fructus" OR "Cardui Mariae Herba" OR "Carduus Marianum" OR "Chardon Argente" OR "Chardon de Marie" OR "Chardon de Notre-Dame" OR "Chardon Marbre" OR "Chardon-Marie" OR "Epine Blanche" OR "Holy Thistle" OR "Lady's Thistle" OR "Lait de Notre-Dame" OR "Legalon" OR "Marian Thistle" OR "Mariendistel" OR "Mary Thistle" OR "Shui

Fei Ji" OR "Silibinin" OR "Silybe de Marie" OR "Silybin" OR "Silybum" OR "Silymarin" OR "Silymarine" OR "St Mary Thistle" OR "St Marys Thistle" OR "Mind-Body Medicine" OR "Mineral Water" OR "Mint*" OR "Mentha aquatica" OR "M aquatica" OR "Mentha palustris" OR "Baume d'Eau" OR "Baume de Riviere" OR "Hierbabuena" OR "Menta del Agua" OR "Menta del Pantano" OR "Menta Vellosa" OR "Yerbabuena" OR "Mentha canadensis" OR "M canadensis" OR "Mentha arvensis var piperascens" OR "Bakha" OR "Cornmint" OR "Menta Japonesa" OR "Mentha Arvensis Aetheroleum" OR "Minzol" OR "Poleo" OR "Pudina" OR "Putiha" OR "Spearmint" OR "Mentha spicata" OR "M spicata" OR "Mentha viridis" OR "Mentha cordifolia" OR "Mentha crispa" OR "Hierbabuena" OR "Menta Verde" OR "Pahari Pudina" OR "Putiha" OR "Sage of Bethlehem" OR "Yerba Buena" OR "Yerbabuena" OR "English Horsemint" OR "Mentha longifolia" OR "M longifolia" OR "Mentha sylvestris" OR "Menta de Caballo" OR "Pudina" OR "Peppermint" OR "Mentha x piperita" OR "Mentha lavanduliodora" OR "Extract of Mentha Piperita" OR "Extrait de Mentha Piperita" OR "Herba Menthae" OR "Huile de Mentha Piperita" OR "M Balsamea" OR "Menta Pepperita" OR "Menta Piperita" OR "Mentha Balsamea" OR "Mentha Oil" OR "Mentha Piperita Extract" OR "Mentha Piperita Oil" OR "Menthae Piperitae Aetheroleum" OR "Menthae Piperitae Folium" OR "Menthe" OR "Menthol" OR "Paparaminta" OR "Mistletoe" OR "Phoradendron leucarpum" OR "P leucarpum" OR "Phoradendron avescens" OR "Phoradendron serontium" OR "Viscum leucarpum" OR "Viscum avescens" OR "Phoradendron macrophyllum" OR "Phoradendron tomentosum" OR "Muerdago Americano" OR "Viscum album" OR "V album" OR "All-Heal" OR "Banda" OR "Blandeau" OR "Bois de Sainte-Croix" OR "Bouchon" OR "Devil's Fuge" OR "Drudenfuss" OR "Eurixor" OR "Guerit-Tout" OR "Gui" OR "Helixor" OR "Herb de Chevre" OR "Hexenbesen" OR "Hurchu" OR "Iscador" OR "Isorel" OR "Leimmistel" OR "Mistlekraut" OR "Mistletein" OR "Muerdago Europeo" OR "Mystyldene" OR "Nid de Sorciere" OR "Pain de Biques" OR "Rini" OR "Verquet" OR "Vert-Bois" OR "Vert de Pommier" OR "Visci" OR "Vogelmistel" OR "Vysorel" OR "Molybdenum" OR "Atomic number 42" OR "Ammonium Molybdate" OR "Chelate de Molybdate" OR "Chelated Molybdenum" OR "Citrate de Molybdene" OR "Etrathiomolybdate" OR "Molibdeno" OR "Molybdate d'Ammonium" OR "Molybdate de Sodium" OR "Molybdene" OR "Sodium Molybdate" OR "Morita therap*" OR "Movement therap*" OR "Moxibustion" OR "Acu-Moxi" OR "Chinetsukyu (Japanese)" OR "Kyutoshin (Japanese)" OR "Moxa" OR "Okyu (Japanese)" OR "Mud therap*" OR "Peloid" OR "Mudras" OR "Mugwort" OR "A vulgaris" OR "Altamisa" OR "Armoise" OR "Artemise" OR "Artemisia" OR "Artemisiae Vulgaris Herba" OR "Artemisiae Vulgaris Radix" OR "Carline Thistle" OR "Felon Herb" OR "Gemeiner Beifuss" OR "Hierba de San Juan" OR "Nagadamni" OR "Remise" OR "Sailor's Tobacco" OR "St. John's Plant" OR "Tabac de Saint-Pierre" OR "Wild Wormwood" OR "Mullein" OR "Verbascum densiflorum" OR "V densiflorum" OR "Verbascum phlomides" OR "V phlomides" OR "Verbascum thapiforme" OR "V thapiforme" OR "Verbascum thapsus" OR "V thapsus" OR "Aaron's Rod" OR "Adam's Flannel" OR "Beggar's Blanket" OR "Blanket Herb" OR "Blanket Leaf" OR "Bouillon Blanc" OR "Bouillon Jaune" OR "Candleflower" OR "Candlewick" OR "Cierge Cotonneux" OR "Cierge de Notre-Dame" OR "Clot-Bur" OR "Clown's Lungwort" OR "Cuddy's Lungs" OR "Duffle" OR "Faux Bouillon-Blanc" OR "Feltwort" OR "Flannelflower" OR "Fleur de Grand Chandelier" OR "Fluffweed" OR "Gidar Tamaku" OR "Gordolobo" OR "Hag's Taper" OR "Hare's Beard" OR "Hedge Taper" OR "Higtaper" OR "Jacob's Staff" OR "Longwort" OR "Molene" OR "Oreille de Loup" OR "Oreille de Saint Cloud" OR "Our Lady's Flannel" OR "Queue de Loup" OR "Rag Paper" OR "Shepherd's Club" OR "Shepherd's Staff" OR "Tabac du Diable" OR "Torches" OR "Velvet Plant" OR "Verbasci Flos" OR

"Wild Ice Leaf" OR "Woolen" OR "Contingent Music" OR "Evocative Music" OR "Expressive therap*" OR "Group Chanting And Singing" OR "Group Drumming" OR "Guided Imagery and Music" OR "Individualized Music-Focused Auditory therap*" OR "Karaoke therap*" OR "Lullaby therap*" OR "Lyric Analysis" OR "Mandalas" OR "Medical Resonance therap* Music" OR "Music And Movement" OR "Music-Assisted Progressive Muscle Relaxation" OR "Music-Assisted Reframing" OR "Music-Based Imagery" OR "Music-Based Intervention" OR "Music Exposure therap*" OR "Music In therap*" OR "Music Intervention" OR "Music Listening Intervention" OR "Music Stimulation" OR "Music therap*" OR "Music-Reinforced therap*" OR "Music-Video therap*" OR "Musical Games" OR "Musical Motor Feedback" OR "Musicokinetic therap*" OR "Ragas" OR "Myofascial Release" OR "MFR therap*" OR "Myotherap*" OR "Soft Tissue Mobilization" OR "Myrrh" OR "Myrrha" OR "Myrrhe" OR "Commiphora molmol" OR "Commiphora habessinica" OR "Commiphora abyssinica" OR "Balsamodendrum habessinicum" OR "Commiphora madagascariensis" OR "Commiphora kataf" OR "Commiphora erythraea" OR "Amyris kataf" OR "Hemprichia erythraea" OR "Commiphora species" OR "Bdellium" OR "Bola" OR "Commiphora" OR "Didin" OR "Didthin" OR "Heerabol" OR "Mirra" OR "Mirrh" OR "Mo Yao" OR "Murrah" OR "Opopanax" OR "Resina Commiphorae" OR "N-Acetylcysteine" OR "Nambudripad's Allergy Elimination therap*" OR "Napratherap*" OR "Nasal Irrigation" OR "Hypertonic Saline Rinse" OR "Irrigacion Nasal" OR "Irrigation Nasale" OR "Jala Neti" OR "Lavage Nasal" OR "Nasal Rinsing" OR "Nasal Saline Irrigation" OR "Neti Pot" OR "Nose Bidet" OR "Pot de Neti" OR "Saline Irrigation" OR "Saline Nasal Irrigation" OR "Sinus Flush" OR "Sinus Rinse" OR "Sinus Rinsing" OR "Natural Medicine" OR "Nature therap*" OR "Naturopat*" OR "Nettle*" OR "Urtica dioica" OR "U dioica" OR "Urtica urens" OR "U urens" OR "Bichu" OR "Feuille d'Ortie" OR "Graine d'Ortie" OR "Ortie" OR "Ortiga" OR "Urtica*" OR "Lamium album" OR "L album" OR "Archangel" OR "Archangelique" OR "Lamier Blanc" OR "Lamii Albi Flos" OR "Neural therap*" OR "Interference Eld" OR "Interference Zone" OR "Neuromuscular therap*" OR "New Age therap*" OR "Noni" OR "Morinda citrifolia" OR "M citrifolia" OR "Ba Ji Tian" OR "Bois Douleur" OR "Canarywood" OR "Cheese Fruit" OR "Hai Ba Ji" OR "Hog Apple" OR "Luoling" OR "Mengkudu" OR "Menkoedoe" OR "Mora de la India" OR "Morinda" OR "Mulberry" OR "Mure Indienne" OR "Nhau" OR "Nono" OR "Nonu" OR "Pau-Azeitona" OR "Rotten Cheese Fruit" OR "Ruibarbo Caribe" OR "Ura" OR "Wild Pine" OR "Wu Ning" OR "Yor" OR "Non-Mainstream Medicine" OR "Non-Orthodox Practice" OR "Non-Pharmacological Intervention" OR "Non-Traditional Medicine" OR "Nuad Bo Rarn" OR "Nutraceuticals" OR "Nutrition therap*" OR "Oatmeal Bath" OR "Ojeok-San" OR "Oleander" OR "Nerium indicum" OR "Nerium odorum" OR "Thevetia peruviana" OR "Cascabela thevetia" OR "Cerbera thevetia" OR "Thevetia neriifolia" OR "Adelfa" OR "Baladre" OR "Exile Tree" OR "Huang Hua Jia" OR "Jia Zhu Tao" OR "Kaner" OR "Karvir" OR "Karvira" OR "Laurel" OR "Laurel Rosa" OR "Laurier Rose" OR "Laurose" OR "Lorier Bol" OR "Nerier a Feuilles de Laurier" OR "Nerion" OR "Oleanderblatter" OR "Oleandre" OR "Oleandri Folium" OR "Rose Bay" OR "Rose Laurel" OR "Soland" OR "Olive*" OR "Olea europaea" OR "O europaea" OR "Acide Gras Insature" OR "Acide Gras Mono-Insature" OR "Acide Gras n-9" OR "Acide Gras Omega 9" OR "Feuille d'Olivier" OR "Huile d'Assaisonnement" OR "Huile d'Olive" OR "Jaitun" OR "Oleae Folium" OR "Olivae Oleum" OR "Olivo" OR "Pulpe d'Olive" OR "Salad Oil" OR "Sweet Oil" OR "M aquifolium" OR "Barberry" OR "Berberis" OR "Mahoni*" OR "Mountain-Grape" OR "Oregon Grape-Holly" OR "Scraperoot" OR "Uva de Oregon" OR "Vigne de l'Oregon" OR "Water-Holly" OR "Organic Food" OR "Alimentation Biologique" OR "Alimentos Organicos" OR "Green Labels" OR

"National Organic Program" OR "Natural Food" OR "Organic Farming" OR "Organic Foods Production Act" OR "USDA Organic" OR "Organotherap*" OR "Ortho-Molecular Medicine" OR "Osteopathic Medicine" OR "Osteopathic Manipulative Treatment" OR "Osteopathy" OR "Joint Manipulation" OR "Musculoskeletal Manipulation" OR "Otikon Otic" OR "Oxygen therap*" OR "Hyperbaric Oxygen" OR "Oxymedicine" OR "Hyperbaric Oxygen" OR "Hyperoxigenation therap*" OR "Bio-Oxidative therap*" OR "Ozonated Autohemotherap*" OR "Ozone" OR "Ozonetherap*" OR "Ozonoterapia" OR "Therapeute par l'Ozone" OR "Therapie a l'Ozone" OR "Therapie par l'Ozone" OR "Paleo Diet" OR "Caveman Diet" OR "Hunter-Gatherer Diet" OR "Paleo Diet" OR "Paleolithic Diet" OR "Stone Age Diet" OR "Palmistry" OR "Palo Santo" OR "Bursera graveolens" OR "B graveolens" OR "Panchakarma" OR "Pancreatic Extract" OR "Passion Flower" OR "Passiflora incarnata" OR "P incarnata" OR "Apricot Vine" OR "Burucuya" OR "Corona de Cristo" OR "Fleischfarbige" OR "Fleur de la Passion" OR "Fleur de Passiflore" OR "Flor de Passion" OR "Granadilla" OR "Grandilla" OR "Grenadille" OR "Madre Selva" OR "Maracuja" OR "Maracuya" OR "Maypop" OR "Pasionari*" OR "Pasiflora" OR "Passiflor*" OR "Passion Vine" OR "Passionaria" OR "Passionblume" OR "Passionflower" OR "Passionsblomma" OR "Passionsblumenkraut" OR "Water Lemon" OR "Passion Fruit" OR "Passiflora edulis" OR "P edulis" OR "Past Life therap*" OR "Pastoral Care" OR "Pau d'Arco" OR "Tabebuia impetiginosa" OR "T impetiginosa" OR "Handroanthus impetiginosus" OR "Tabebuia avellanedae" OR "Tabebuia heptaphylla" OR "Tabebuia palmeri" OR "Tecoma impetiginosa" OR "Benier de Guyane" OR "Ebene Vert" OR "Ipe*" OR "Lapacho" OR "Lebene" OR "Pink Trumpet Tree" OR "Quebracho" OR "Taheebo" OR "Trumpet Bush" OR "PC-SPES" OR "Pectin*" OR "Acide Pectique" OR "MCP" OR "Pennyroyal" OR "Mentha pulegium" OR "M pulegium" OR "Pulegium vulgare" OR "Hedeoma pulegioides" OR "Melissa pulegioides" OR "Dictame de Virginie" OR "Fretillet" OR "Lurk-In-The-Ditch" OR "Mosquito Plant" OR "Piliolerial" OR "Poleo" OR "Pouliot" OR "Pulegium" OR "Run-By-The-Ground" OR "Squaw Balm" OR "Squawmint" OR "Stinking Balm" OR "Tickweed" OR "Pennywort" OR "Gotu Kola" OR "Centella asiatica" OR "C asiatica" OR "Hydrocotyle asiatica" OR "Brahma-Buti" OR "Brahma-Manduki" OR "Brahmi" OR "Bua-Bok" OR "Centella" OR "Centellase" OR "Divya" OR "Hydrocotyle" OR "Indischer Wassernabel" OR "Idrocotyle" OR "Indian Water Navelwort" OR "Ji Xue Cao" OR "Khulakhudi" OR "Luei Gong Gen" OR "Luo De Da" OR "Madecassol" OR "Mandukaparni" OR "Manduk Parani" OR "Mandukig" OR "Marsh Penny" OR "Talepetrako" OR "Tsubo-kusa" OR "Tungchian" OR "White Rot" OR "Bacopa" OR "B monnieri" OR "Herpestis monniera" OR "Moniera cuneifolia" OR "Andri" OR "Brahmi" OR "Herb of Grace" OR "Herpestis Herb" OR "Hysope d'Eau" OR "Jalanimba" OR "Jal-Brahmi" OR "Jalnaveri" OR "Nira-Brahmi" OR "Sambrani Chettu" OR "Thyme-Leaved Gratiola" OR "Water Hyssop" OR "Peony" OR "Paeonia lactiflora" OR "P lactiflora" OR "Paeonia albiflora" OR "P albiflora" OR "Paeonia mascula" OR "P mascula" OR "Paeonia arietina" OR "P arietina" OR "Paeonia caucasica" OR "P caucasica" OR "Paeonia corallina" OR "P corallina" OR "Paeonia coriacea" OR "P coriacea" OR "Paeonia daurica" OR "P daurica" OR "Paeonia kavachensis" OR "P kavachensis" OR "Paeonia triternata" OR "P triternata" OR "Paeonia obovata" OR "P obovata" OR "Paeonia japonica" OR "P japonica" OR "Paeonia obovata" OR "P obovata" OR "Paeonia willmottiae" OR "P willmottiae" OR "Paeonia officinalis" OR "P officinalis" OR "Paeonia microcarpa" OR "P microcarpa" OR "Paeonia paradoxa" OR "P paradoxa" OR "Paeonia suffruticosa" OR "P suffruticosa" OR "Paeonia arborea" OR "P arborea" OR "Paeonia moutan" OR "P moutan" OR "Paeonia anomala" OR "P anomala" OR "Paeonia veitchii" OR "P veitchii" OR "Paeonia beresowskii" OR "P beresowskii" OR "Paeonia

woodwardii" OR "P woodwardii" OR "Bai Shao" OR "Chi Shao" OR "Cortex Moutan" OR "Jiu Chao Bai Shao" OR "Moutan" OR "Mu Dan Pi" OR "Paeoniae Alba" OR "Paeoniae Flos" OR "Paeoniae Radix" OR "Peonia" OR "Piney" OR "Pivoine" OR "Radix Paeoniae" OR "Radix Paeoniae Rubra" OR "Shakuyaku" OR "Shao Yao" OR "Ud Saleeb" OR "Udsalam" OR "Udsalap" OR "Phenylalanine" OR "2-amino-3-phenyl-propanoic acid" OR "Acide Alpha-aminohydrocinnamique" OR "Alpha aminohydrocinnamic Acid" OR "Beta-phenyl-alanine" OR "Beta-phenyl-alanine" OR "D-Phenylalanine" OR "DL-Phenylalanine" OR "Fenilalanina" OR "L-Phenylalanine" OR "Phosphatidyl Choline" OR "Phosphatidylserine" OR "Fosfatidilserina" OR "Phosphatidyl Serine" OR "PtdSer" OR "Soy-PS" OR "Physical therap*" OR "Physical Manipulation" OR "Physical Medicine" OR "Manupulative therap*" OR "Soft Tissue Manipulation" OR "Phytotherap*" OR "Phytomedicine" OR "Phytoestrogen" OR "Phytoestrol" OR "Phytonutrient" OR "Phytopharma" OR "Phytosterol" OR "Pilates" OR "Controlog*" OR "Plant-Based Medicines" OR "Floral therap*" OR "Pine-Bark Extract" OR "Placebo Plant" OR "Plant Extracts" OR "Plant Oils" OR "Plant-Based Medications" OR "Play therap*" OR "Polarity therap*" OR "Energy Medicine" OR "Energy therap*" OR "Energy Work" OR "Polarit*" OR "Terapia de Polaridad" OR "Therapie Energetique" OR "Therapie de l'Energie" OR "Pomegranate" OR "Punica granatum" OR "P granatum" OR "Anardana" OR "Dadim" OR "Dadima" OR "Delima" OR "Fruit of the Dead" OR "Gangsalan" OR "Granaatappel" OR "Granad*" OR "Granatapfel" OR "Grenad*" OR "Limoni" OR "Melogranato" OR "Melograno Granato" OR "Pomme Grenade" OR "Pomo Granato" OR "Pomo Punico" OR "PPE" OR "Roma" OR "Romazeira" OR "Romeira" OR "Shi Liu Gen Pi" OR "Shi Liu Pi" OR "Tab Tim" OR "Positive Intention Practice" OR "Postural Realignment" OR "Potassium" OR "Atomic number 19" OR "Numero Atomique 19" OR "Potasio" OR "Pranic Healing" OR "Prayer" OR "Distant Healing" OR "Faith Healing" OR "Spiritual therap*" OR "Christian Science" OR "Mysticism" OR "Absent Healing" OR "Attitudinal Healing" OR "Compassion And Healing" OR "Compassionate Intention" OR "Distant Healing" OR "Divining" OR "External Qigong" OR "Intentionality" OR "Kahuna Healing" OR "Native American Faith Healing" OR "Noetic therap*" OR "Psychic Healing" OR "Quantum Healing" OR "Reiki" OR "Remote Healing" OR "Spiritual Healing" OR "Sufi Healing" OR "Prebiotics" OR "Preventative Medicine" OR "Primitive Medicine" OR "Pritkin Diet" OR "Probiotics" OR "Progressive Muscular Relaxation" OR "Progressive Relaxation" OR "Prolotherap*" OR "Nonsurgical Reconstructive therap*" OR "Proliferative therap*" OR "Reconstructive therap*" OR "Propolis" OR "Acide de Cire d'Abeille" OR "Bee Glue" OR "Beeswax Acid" OR "Cire d'Abeille Synthetique" OR "Colle d'Abeille" OR "Hive Dross" OR "Penicilline Russe" OR "Propoleos" OR "Russian Penicillin" OR "Synthetic Beeswax" OR "Yellow Propoli" OR "Protein Diet" OR "Protein Restricted Diet" OR "Psychic Medicine" OR "Psychic Healing" OR "Psychoneuroimmunology" OR "Psychotherap*" OR "Psychodrama" OR "Psyllium" OR "Plantago arenaria" OR "P arenaria" OR "Plantago afra" OR "Plantago indica" OR "Dietary Fiber" OR "Erva-das-Pulgas" OR "Fibre Alimentaire" OR "Fleaseed" OR "Fleawort" OR "Flohkraut" OR "Flohsamen" OR "Herbes aux Puces" OR "OEil-de-Chien" OR "Pilicaire" OR "Plantain" OR "Psyllii Semen" OR "Psyllion" OR "Psyllios" OR "Puciere" OR "Pucilaire" OR "Scharzer Flohsame" OR "Zaragatona" OR "Plantago ovata" OR "P ovata" OR "Plantago fastigiata" OR "Plantago insularis" OR "Plantago ispaghula" OR "Plantago decumbens" OR "Blond Plantago" OR "Che Qian Zi" OR "Dietary Fiber" OR "Englishman's Foot" OR "Fibre Alimentaire" OR "Indian Plantago" OR "Ipagula" OR "Isabgola" OR "Isabgul" OR "Ispaghul" OR "Ispaghula" OR "Ispagol" OR "Plantaginis Ovatae Semen" OR "Plantaginis Ovatae Testa" OR "Psilio" OR "Psillium Blond" OR "Spogel" OR "Puerarin"

OR "Pulsed therap*" OR "Pumpkin Seed" OR "Purple Sweet Potato" OR "Purple Yam" OR "Pygeum" OR "Prunus africana" OR "P africana" OR "African Plum Tree" OR "African Prune" OR "Amande Amere" OR "Ciruelo Africano" OR "Prunier d'Afrique" OR "Pyrrolizidine Alkaloid" OR "Qi Gong" OR "Ba Duan Jin" OR "Biyun Method" OR "Chan Mi Gong" OR "Chi'I Kung" OR "Chi Gung" OR "Chi Kung" OR "Dantian" OR "Energy Healing" OR "Energy Health" OR "Guolin" OR "Hua Gong" OR "Qigong" OR "Qigongology" OR "Wuqinxi" OR "Yi Jia Gong" OR "Quantum Healing" OR "Quantum Medicine" OR "Radiance Technique" OR "Radiation therap*" OR "Radiesthesia" OR "Radisthesis" OR "Rapid Eye Technology" OR "Rebirthing" OR "Trifolium pratense" OR "Beebread" OR "Clovone" OR "Daidzein" OR "Genistein" OR "Miel des Pres" OR "Phytoestrogen" OR "Trebol Rojo" OR "Trefle Commun" OR "Trefle des Pres" OR "Trefle Pourpre" OR "Trefle Rouge" OR "Trefle Rougeatre" OR "Trefle Violet" OR "Trefoil" OR "Trifolium" OR "Red Yeast Rice" OR "Monascus purpureus" OR "Arroz de Levadura Roja" OR "Cholestin" OR "Hong Qu" OR "Hongqu" OR "Koji Rouge" OR "Mevinolin" OR "Monacolin K" OR "Monascus" OR "Red Koji" OR "Red Rice" OR "Riz Rouge" OR "Rotschimmelreis" OR "XueZhiKang" OR "Xue Zhi Kang" OR "Zhibituo" OR "Zhitai" OR "Zhi Tai" OR "Reflex Locomotion" OR "Reflexology" OR "Foot Therap*" OR "Reflexologia" OR "Reflexologie" OR "Reflexologist" OR "Reflexologue" OR "Reflexotherap*" OR "Reflexotherapie" OR "Therapie de Zone" OR "Zone Therap*" OR "Regression Therap*" OR "Reichian" OR "Bioenergy Therap*" OR "Biofield Energy Therap*" OR "Energie Universelle de Vie" OR "Healing Touch" OR "Ray-kee" OR "Therapeutic Touch" OR "Therapie manuelle Energetique" OR "Therapie par le Toucher" OR "Universal Life Energy" OR "Champignon Basidiomycete" OR "Champignon d'Immortalite" OR "Ganoderma" OR "Ling Chih" OR "Ling Zhi" OR "Mannentake" OR "Mushroom*" OR "Reishi" OR "Rei-Shi" OR "Spirit Plant*" OR "Relaxation Therap*" OR "Applied Relaxation" OR "Relaxation Appliquee" OR "Relaxation Training" OR "Relaxation Treatment" OR "Terapia de Relajacion" OR "Therapeutic Relaxation" OR "Therapie de Relaxation" OR "Traitement de Relaxation" OR "Relaxation Class*" OR "Relaxation Response*" OR "Relaxation Tape*" OR "Relaxation Technique*" OR "Guided Relaxation" OR "Religious Therap*" OR "Religion and Medicine" OR "Religious Healing" OR "Restricted Environmental Stimulation Therap*" OR "Resveratrol*" OR "3,4',5-stilbenetriol" OR "3,5,4'-trihydroxystilbene" OR "3,4',5-trihydroxystilbene" OR "3,5,4'-trihydroxy-trans-stilbene" OR "3,5,4' TriHydroxy-Transstibene" OR "(E)-5-(4-hydroxystyryl)benzene-1,3-diol" OR "Cis-Resveratrol" OR "Extrait de Vin" OR "Kojo-Kon" OR "Phytoalexin*" OR "Phytoestrogen" OR "Phyto-oestrogene" OR "Pilule de Vin" OR "Protykin" OR "Trans-Resveratrol" OR "Wine Extract" OR "Wine Pill" OR "Revici Method" OR "Rhodiola" OR "Sedum rosea" OR "Arctic Root" OR "Golden Root" OR "Hongjingtian" OR "Hong Jing Tian" OR "King's Crown" OR "Lignum Rhodium" OR "Orpin Rose" OR "Racine d'Or" OR "Racine Doree" OR "Rhodiole" OR "Rodia Riza" OR "Rose Root" OR "Rosenroot" OR "Roseroot" OR "Rosewort" OR "Snowdown Rose" OR "Rice Bran" OR "Oryza sativa" OR "Cereal Fiber" OR "Dietary Fiber" OR "Fibre Alimentaire" OR "Fibre Cerealiere" OR "Ricebran Oil" OR "Riz de Son" OR "Salvado de Arroz" OR "Son de Riz" OR "Rolfing" OR "Integration Structurelle" OR "Manipulative therap*" OR "Medecine Physique" OR "Physical Medicine" OR "Rolfer" OR "Therapie Manuelle" OR "Rongoa" OR "Rosen Method" OR "Rubenfeld Synergy" OR "Sacred Healing" OR "Safflower Yellow Injection" OR "Saffron" OR "Crocus sativus" OR "Autumn Crocus" OR "Azafran" OR "Azafron" OR "Croci Stigma" OR "Crocus Cultive" OR "Kashmira" OR "Kesar" OR "Kumkuma" OR "Safran" OR "Zafran" OR "Salacia" OR "Chundan" OR "Kothala Himbutu" OR "Ponkoranti" OR "S oblonga" OR "Diviners Sage" OR "Divinorin" OR "Feuilles de la Bergere" OR

"Feuilles de la Vierge" OR "Herb-of-the-Virgin" OR "Herb of Mary" OR "Herba de Maria" OR "Hierba de la Virgen" OR "Hierba Maria" OR "Hojas de la Pastora" OR "Hojas de Maria" OR "La Hembra" OR "Leaf of Mary" OR "Menthe Magique" OR "Mexican Sage" OR "Pipiltzintzintli" OR "Sadi" OR "Sally-D" OR "Salvia" OR "Salvinorin" OR "Sage of the Seers" OR "Sauge des Devins" OR "Sauge Divinatoire" OR "Shepherdess" OR "Ska Maria" OR "Ska Pastora" OR "Yerba de Maria" OR "Yerba Maria" OR "Samadhi" OR "S-adenosyl-L-methionine" OR "Ademetionine" OR "Adenosylmethionine" OR "S-Adenosyl Methionine" OR "S-Adenosyl-L-Methionine" OR "S-Adenosylmethionine" OR "SAM-e" OR "Samyr" OR "Samyama" OR "Sanchi Preparation" OR "Sand Bath" OR "Sauna" OR "Saw Palmetto" OR "Serenoa repens" OR "S repens" OR "Serenoa serrulata" OR "S serrulata" OR "Sabal serrulata" OR "American Dwarf Palm Tree" OR "Baies du Palmier Scie" OR "Cabbage Palm" OR "Chou Palmiste" OR "Ju-Zhong" OR "Palma Enana Americana" OR "Palmier de Floride" OR "Palmier Nain" OR "Palmier Scie" OR "Sabal" OR "Saw Palmetto Berry" OR "Scraping" OR "Coining" OR "Gua Sha" OR "Kerokan Spooning" OR "Seaweed" OR "Sectarian Medicine" OR "Selenium" OR "Atomic number 34" OR "Ebselen" OR "L-Selenomethionine" OR "Levure Selenisee" OR "Numero Atomique 34" OR "Selenio" OR "Selenite" OR "Selenized Yeast" OR "Selenomethionine" OR "Self Help" OR "Self-Awareness" OR "Self-Care" OR "Self-Healing Abilities" OR "Self-Help" OR "Self-Massage" OR "Self-Medication" OR "Senna" OR "Casse" OR "Fan Xie Ye" OR "Sen" OR "Sena Alejandrina" OR "Sene" OR "Sennae Folium" OR "Sennae Fructus" OR "Sennosides" OR "Sensory Therap*" OR "Shamanism" OR "Shamanic Healing" OR "Shengmai" OR "Shenmai" OR "Shenqi Fuzheng" OR "Shensu" OR "Shenfu" OR "Shexiang" OR "Shiatsu" OR "Finger Pressure" OR "Medecine Energetique" OR "Ohashiatsu" OR "Sante Energetique" OR "Shi-astsu" OR "Shiatsupractor" OR "Lentinus edodes" OR "Lenticus edodes" OR "Lentinula edodes" OR "Tricholomopsis edodes" OR "Champignon Noir" OR "Champignon Parfume" OR "Hua Gu" OR "Lentin" OR "Lentinula" OR "Pasania Fungus" OR "Shiitake" OR "Shitake" OR "Snake Butter" OR "Xiang-Gu" OR "Shuanghuanglian" OR "Siddha" OR "Silicon" OR "Si" OR "Atomic number 14" OR "Acide Orthosilicique" OR "Numero Atomique 14" OR "Orthosilicic Acid" OR "Polysilicone-11" OR "Silica" OR "Silice Hydride" OR "Silicea" OR "Silicio" OR "Silicium" OR "Sodium Silicate" OR "Sintergetic Medicine" OR "Snake Venom" OR "Snoezelen" OR "Controlled Multisensory Environment" OR "Social Thermalism" OR "Sodium Restricted Diet" OR "Somatherap*" OR "Sophrology" OR "Soul Retrieval" OR "Sound Healing" OR "Vibration Healing" OR "Soy" OR "Glycine max" OR "Glycine gracilis" OR "G gracilis" OR "Glycine hispida" OR "Phaseolus max" OR "Daidzein*" OR "Edamame" OR "Estrogene Vegetal" OR "Genistein*" OR "Isoflavone*" OR "Legume" OR "Miso" OR "Natto" OR "Phytoestrogen" OR "Phyto-oestrogene" OR "Plant Estrogen" OR "Shoyu" OR "Soja" OR "Sojabohne" OR "Soya" OR "Soybean" OR "Tempeh" OR "Texturized Vegetable Protein" OR "Tofu" OR "Touchi" OR "Spa" OR "Speleotherap*" OR "Spinal Manipulation" OR "Spot therap*" OR "St John's Wort" OR "Hypericum perforatum" OR "H perforatum" OR "Amber" OR "Barbe de Saint-Jean" OR "Chasse-Diable" OR "Demon Chaser" OR "Fuga Daemonum" OR "Goatweed" OR "Hardhay" OR "Hierba de San Juan" OR "Hypereikon" OR "Hyperici Herba" OR "Hypericum" OR "Klamathaweed" OR "Millepertuis" OR "Rosin Rose" OR "Saynt Johannes Wort" OR "Stanol therap*" OR "Sitostanol" OR "3-beta,5-alpha-stigmastan-3-ol" OR "24-alpha-ethylcholestanol" OR "Beta-sitostanol" OR "Dihydro-beta-sitosterol" OR "Fucostanol" OR "Phytostanol" OR "Plant Stanol" OR "Stanol Vegetal" OR "Stigmastanol" OR "Plant Sterol" OR "(24S)-5,22-Stigmastadien-3-beta-ol" OR "3-beta-stigmast-5-en-3-ol, 22,23-dihydrostigmasterol"

OR "24-beta-ethyl-delta-5-cholesten-3-beta-ol" OR "24-ethyl-cholestero" OR "Avenasterol" OR "B-sitosterol 3-B-D-glucoside" OR "B-Sitosterolin" OR "B-Sitosterols" OR "Beta-sitosterine" OR "Beta-Sitosterol" OR "Betasitosterol" OR "Brassicasterol" OR "Campest-5-en-3beta-ol" OR "Campesterol" OR "Cinchol" OR "Cupreol" OR "Dihydro-beta-sitosterol" OR "Ester de Sterol Vegetal" OR "Esters de Sterol Derives d'huile Vegetale" OR "Phytosterol*" OR "Plant Sterolins" OR "Quebrachol" OR "Rhamnol" OR "Sitosterin" OR "Sitosterol*" OR "Sterinol" OR "Sterolin*" OR "Sterols Vegetaux" OR "Stigmasterin" OR "Stigmasterol" OR "Vegetable Oil Sterol Esters" OR "Vegetable Sterol Esters" OR "Staphage Lysate" OR "Stem Cell Therap*" OR "Commercial Stem Cell Transplant*" OR "Mesenchymal Stromal Cell Therap*" OR "Stem Cell-Based Interventions" OR "Stem Cell Clinics" OR "Stem Cell Tourism" OR "Stem Cell Treatments" OR "Stevia" OR "Eupatorium rebaudianum" OR "Mustelia eupatoria" OR "Azucacaa" OR "Caa-He-E" OR "Caa'Inhem" OR "Ca-A-Jhei" OR "Ca-A-Yupi" OR "Capim Doce" OR "Chanvre d'Eau" OR "Eira-Caa" OR "Erva Doce" OR "Estevia" OR "Kaa Jhee" OR "Plante Sucree" OR "Reb A" OR "Rebaudioside A" OR "Rebiana" OR "Stevioside" OR "Sweet Herb" OR "Sweet Leaf of Paraguay" OR "Sweetleaf" OR "Yerba Dulce" OR "Strength Training" OR "Stress Reduction" OR "Stretch" OR "Hold Relax" OR "Sulfinic Acid" OR "Suxiao Jiuxin Wan" OR "Symbolic Action" OR "Symbolic Chant" OR "Symbolic Movement" OR "Tabebuia" OR "Tactile therap*" OR "Tai Chi" OR "Art Martial Interne" OR "Internal Martial Art" OR "Meditation en Mouvement" OR "Moving Meditation" OR "Tai-Chi" OR "Taichi Quan" OR "Tai Ji Quan" OR "Taiji" OR "Taijiquan" OR "Tie Chee" OR "Tao" OR "Taurine" OR "2-Aminoethane Sulfonic Acid" OR "2-Aminoethylsulfonic Acid" OR "Aminoethanesulfonate" OR "Aminoethylsulfonique" OR "Dibicor" OR "L-Taurine" OR "Taurina" OR "Taurine Ketoisocaproic Acid" OR "Melaleuca alternifolia" OR "M alternifolia" OR "Aceite del Arbol de Te" OR "Huile de Melaleuca" OR "Huile de Theier" OR "Huile Essentielle de Theier" OR "Melaleuca Oil" OR "Oil of Melaleuca" OR "Oleum Melaleucae" OR "Tea Tree" OR "Ti Tree Oil" OR "Transcutaenous Electrical Nerve Stimulation therap*" OR "Testosterone Enhancement" OR "Thalassotherap*" OR "Hydrotherap*" OR "Therapeutic Counsel" OR "Therapeutic Crisis Intervention" OR "Thermotherap*" OR "Heat Therap*" OR "Therapeutic Hypothermia" OR "Therapeutic Hyperthermia" OR "Induced Hypothermia" OR "Induced Hyperthermia" OR "Thomsonianism" OR "Thought Field therap*" OR "Mental Field therap*" OR "Thought Therap*" OR "Threonine" OR "L-Threonine" OR "Treonina" OR "Thunder God Vine" OR "Tripterygium wilfordii" OR "Huang-T'eng Ken" OR "Lei Gong Teng" OR "Lei-Kung T'eng" OR "Seven Step Vine" OR "Taso-Ho-Hua" OR "Threewingnut" OR "Tonnerre de la Vigne de Dieu" OR "Tripterigium Wilfordii" OR "Vigne du Tonnerre Divin" OR "Yellow Vine" OR "Thyme" OR "Farigoule" OR "Farigoulette" OR "Frigoule" OR "Mignotise des Genevois" OR "Pote" OR "Serpolet" OR "Thym" OR "Thymi herba" OR "Tomillo" OR "Van Ajwayan" OR "Vanya Yavani" OR "Iper" OR "Serpyllum" OR "Complexe de Peptides Thymiques" OR "Extracto de Timo" OR "Extrait Thymique" OR "Proteine Thymique" OR "Thymic Extract" OR "Thymic Peptide" OR "Thymic Protein" OR "Thymomodulin" OR "Thymosin*" OR "Thymostimulin" OR "Thymostimuline" OR "Thymus" OR "Thymus-Derived Polypeptides" OR "Tianmadingxian Capsule" OR "Tinospora cordifolia" OR "Ambervel" OR "Amrita" OR "Amritha" OR "Gilo" OR "Giloe" OR "Giloy" OR "Glunchanb" OR "Guduchi" OR "Gulancha Tinospora" OR "Gulvel" OR "Gurcha" OR "Heavenly Elixir" OR "Indian Tinospora" OR "Jetwatika" OR "Mehahara" OR "Mehaghna" OR "Moonseed" OR "Pramehahara" OR "Pramehaghna" OR "Sindal" OR "Sittamrytu" OR "Somida" OR "Tinospora" OR "Tinosporia Cordifolus" OR "Tissue Therap*" OR "Topical Therap*" OR "Bioenergy Healing" OR "Biofield Therap*" OR "Spiritual Healing" OR

"Toque Terapeutico" OR "Touch Therap*" OR "Toucher Therapeutique" OR "Toucher Guerisseur" OR "TT" OR "Traditional Medicine" OR "First Nation Tradition" OR "Indigenous Medicine" OR "Native American Medicine" OR "Prophetic Medicine" OR "Traditional Ancestral Medicine" OR "Traditional Herbal Medicine" OR "Traditional Asian Healing" OR "Traditional Bhutanese Medicine" OR "Traditional Birth Attendants" OR "Traditional Cautery" OR "Traditional Chinese Herbal Remedies" OR "Traditional Chinese Medicine" OR "Traditional Eastern And Western Medicine" OR "Traditional European Healing Methods" OR "Traditional European Medicine" OR "Traditional Healer" OR "Traditional Healing Practices" OR "Traditional Indian Medicine" OR "Traditional Korean Medicine" OR "Traditional Malay Medicine" OR "Traditional Maori Healing" OR "Traditional Medication" OR "Traditional Midwivery" OR "Traditional Mongolian Medicine" OR "Traditional Oriental Medicine" OR "Traditional Persian Medicine" OR "Traditional South American Medicine" OR "Traditional Therapeutic Exercises" OR "Traditional Tongan Medicine" OR "Approche Pyscho-Corporelle" OR "Manual Therap*" OR "Mentastics" OR "Therapie Corporelle" OR "Therapie Manuelle" OR "Trager" OR "Transcendental Medicine" OR "Transcendental Meditation" OR "Transcranial Magnetic Stimulation" OR "Transpersonal Psychology" OR "Trigger Point Therap*" OR "Dry Needling" OR "Neuromuscular Therap*" OR "Positional Release Therap*" OR "Trigger Point Injection" OR "Trigger Point Management" OR "Turmeric" OR "Curcuma" OR "Curcumae Longa" OR "Curcumae Longae Rhizoma" OR "Curcumin*" OR "Halada" OR "Haldi" OR "Haridra" OR "Indian Saffron" OR "Nisha" OR "Pian Jiang Huang" OR "Radix Curcumae" OR "Rajani" OR "Rhizoma Cucurmae Longae" OR "Yu Jin" OR "Curcumae Xanthorrhizae Rhizoma" OR "Temoe-Lawacq" OR "Temoe-Lawaq" OR "Temu Lawak" OR "Temu Lawas" OR "Tewon Lawa" OR "B aristata" OR "Chitra" OR "Citra" OR "Darhahed" OR "Darhald" OR "Daruhaldi" OR "Daruharidra" OR "Darurajani" OR "Darvi" OR "Epine-Vinette Aristee" OR "Hint Amberparisi" OR "Indian Berberry" OR "Indian Lycium" OR "Pisse Vinaigre" OR "Vinettier Ariste" OR "Tyrosine" OR "2-Acetylamino-3-(4-Hydroxyphenyl)-Propanoic Acid" OR "2-Amino-3-(4-Hydroxyphenyl)-propionic acid" OR "Acetyl-L-Tyrosine" OR "L-Tyrosine" OR "N-Acetyl-L-Tyrosine" OR "N-Acetyl-Tyrosine" OR "Tirosina" OR "Tyrosinum" OR "Graeco-Arabic Medicine" OR "Medecine Greco-Arabe" OR "Unaani" OR "Unani" OR "Yunani" OR "Unconventional Medicine" OR "Unconventional therap*" OR "Unconventional Approach" OR "Unconventional Health" OR "Unorthodox Practice" OR "Urine therap*" OR "Amaroli" OR "Auto-Urine therap*" OR "Auto-Urotherap*" OR "Mutra Paribhasa" OR "Mutra Varga" OR "Naramutra" OR "Shivambu" OR "Uro-therap*" OR "Urotherap*" OR "Valerian" OR "All-Heal" OR "Amantilla" OR "Baldrian" OR "Baldrianwurzel" OR "Garden Heliotrope" OR "Guerit Tout" OR "Tagar" OR "Tagar-Ganthoda" OR "Tagara" OR "Valeriana" OR "Valerianae Radix" OR "Valeriane" OR "Centranthus ruber" OR "Alfenique" OR "Barbe de Jupiter" OR "Bouncing Bess" OR "Bovis and Soldier" OR "Centranthe Rouge" OR "Centranto" OR "Delicate Bess" OR "Drunken Sailor" OR "Fox's-Brush" OR "Jupiter's Beard" OR "Lilas d'Espagne" OR "Milamores" OR "Pretty Betsy" OR "Vanadium" OR "Atomic number 23" OR "Metavanadate" OR "Orthovanadate" OR "Vanadate" OR "Vanadio" OR "Vanadyl" OR "Vanilla" OR "Myrobroma fragrans" OR "Vainilla" OR "Vanille" OR "Vegan Diet" OR "Vegetarian Diet" OR "Flexitarian Diet" OR "Fruitarianism Diet" OR "Lacto-Vegetarian Diet" OR "Ovo-Vegetarian Diet" OR "Pescotarian Diet" OR "Plant-Based Diet" OR "Semi-vegetarian Diet" OR "Vegetarianism" OR "Whole Food Plant-Based Diet" OR "Vitamin A" OR "3-Dehydroretinol" OR "Antixerophthalmic Vitamin" OR "Axerophtholum" OR "Dehydroretinol" OR "Fat-Soluble Vitamin" OR "Oleovitamin A" OR "Retinoid*" OR "Retinol" OR

"Retinyl Acetate" OR "Retinyl Palmitate" OR "Retinyl Palmitate" OR "Vitamin A1" OR "Vitamin A2" OR "Vitamina A" OR "Vitamine A" OR "Vitamine A1" OR "Vitamine A2" OR "Vitamine Liposoluble" OR "Vitaminum A" OR "Vitamin B12" OR "Cyanocobalamin*" OR "Hydroxocobalamin*" OR "Methylcobalamin*" OR "B-12" OR "B12" OR "B Complex" OR "Bedumil" OR "Cobalamin*" OR "Cobamin*" OR "Complexe Vitaminique B" OR "Cycobemin" OR "Hydroxocobemine" OR "Idrossocobalamina" OR "Vitadurin*" OR "Vitamina B12" OR "Vitamine B12" OR "Vitamin B2" OR "Riboflavin*" OR "Complexe de Vitamines B" OR "Flavin*" OR "Lactoflavin*" OR "Vitamin G" OR "Vitamina B2" OR "Vitamine B2" OR "Vitamine G" OR "Vitamin B3" OR "Niacin*" OR "3-Pyridinecarboxylic Acid" OR "Nicotinic acid" OR "Acide Nicotinique" OR "Acide Pyridine-Carboxylique-3" OR "Anti-Blacktongue Factor" OR "Antipellagra Factor" OR "Facteur Anti-Pellagre" OR "Nicosedine" OR "Pellagra Preventing Factor" OR "Vitamin PP" OR "Vitamina B3" OR "Vitamine B3" OR "Vitamine PP" OR "Niacinamide" OR "Pyridine-3-carboxamide" OR "3-Pyridine Carboxamide" OR "3-Pyridinecarboxamide" OR "Amide de l'Acide Nicotinique" OR "Niacinamida" OR "Nicamid" OR "Nicosedine" OR "Nicotinamide" OR "Nicotinic Acid Amide" OR "Nicotylamidum" OR "Vitamin B3a" OR "Vitamina B3" OR "Vitamine B3" OR "Vitamin B5" OR "D-Pantothenic Acid" OR "Pantothenic Acid" OR "Acide D-Pantothenique" OR "Acide Pantothenique" OR "Acido Pantotenico" OR "Alcool Pantothenylique" OR "Calcii P antothenas" OR "D-Panthenol" OR "D-Pantothenate de Calcium" OR "D-Pantothenyl Alcohol" OR "Dexpanthenol" OR "Dexpanthenolum" OR "Pantethine" OR "Panthenol" OR "Pantothenate" OR "Pantothenate" OR "Pantothenol" OR "Pantothenylol" OR "Vitamin B-5" OR "Vitamina B5" OR "Vitamine B5" OR "Vitamin B6" OR "Pyridoxine" OR "Pyridoxal" OR "Pyridoxamine" OR "Pyridoxine-5'-Phosphate" OR "Pyridoxal-5'-Phosphate" OR "Pyridoxamine-5'-Phosphate" OR "Adermine Chlorhydrate" OR "Adermine Hydrochloride" OR "B6" OR "Phosphate de Pyridoxal" OR "Piridoxina" OR "Pyridoxal Phosphate" OR "Pyridoxal 5 Phosphate" OR "Pyridoxal-5-Phosphate" OR "Pyridoxine-5-Phosphate" OR "P5P" OR "P-5-P" OR "Vitamin B-6" OR "Vitamina B6" OR "Vitamine B6" OR "Vitamin B7" OR "Cis-hexahydro-2-oxo-1H-thieno[3,4-d]-imidazole-4-valeric acid" OR "Biotin" OR "Biotina" OR "Biotine" OR "Biotine-D" OR "Coenzyme R" OR "D-Biotin" OR "Vitamin H" OR "Vitamine B7" OR "Vitamine H" OR "W Factor" OR "Vitamin C" OR "Ascorbic Acid" OR "Acide Ascorbique" OR "Acide Cevitamique" OR "Acide Iso-Ascorbique" OR "Acide L-Ascorbique" OR "Acido Ascorbico" OR "Antiscorbutic Vitamin" OR "Ascorbate" OR "Ascorbyl Palmitate" OR "Cevitamic Acid" OR "Iso-Ascorbic Acid" OR "L-Ascorbic Acid" OR "Palmitate d'Ascorbyl" OR "Vitamina C" OR "Vitamine Antiscorbutique" OR "Vitamine C" OR "Vitamin D" OR "1, 25-Dihydroxycholecalciferol" OR "25-Hydroxycholecalciferol" OR "Alfacalcidol" OR "Calcifediol" OR "Calcipotriene" OR "Calcitriol" OR "Cholecalciferol" OR "Dihydrotachysterol" OR "Ergocalciferol" OR "Paricalcitol" OR "Alfacalcidol" OR "1-Alpha-Hydroxycholecalciferol" OR "1-Alpha-Hydroxycholecalciferol" OR "1alpha(OH)D3" OR "Vitamina D" OR "Vitamine D" OR "25-HCC" OR "25-hydroxycholecalciferol" OR "25-hydroxyvitamin D3" OR "25-hydroxyvitamine D3" OR "25-OHCC" OR "25-OHD3" OR "Calcipotriene" OR "Calcipotriol" OR "1,25-DHCC" OR "1,25-dihydroxycholecalciferol" OR "1,25-dihydroxyvitamin D3" OR "1,25-dihydroxyvitamine D3" OR "1,25-diOHC" OR "1,25(OH)2D3" OR "Cholecalciferol" OR "7-dehydrocholesterol Active" OR "Activated 7-dehydrocholesterol" OR "Colecalciferol" OR "Vitamin D3" OR "Dihydrotachysterol" OR "dihydrotachysterol 2" OR "dichysterol" OR "Vitamine D3" OR "Ergocalciferol" OR "Activated Ergosterol" OR "Calciferol" OR "Ergocalciferol" OR "Ergocalciferolum" OR "Ergosterol Active" OR "Irradiated Ergosterol" OR

"Ergosterol Irradie" OR "Viosterol" OR "Vitamin D2" OR "Vitamine D2" OR "Paricalcitol" OR "19-nor-1,25-dihydroxyvitamin D2" OR "19-nor-1,25-dihydroxyvitamine D2" OR "Paracalcin" OR "Vitamin E" OR "Alpha-Tocopherol" OR "Beta-Tocopherol" OR "Delta-Tocopherol" OR "Gamma-Tocopherol" OR "Acetate d'Alpha Tocopheryl" OR "Acetate de Tocopherol" OR "Acetate de Tocopheryl" OR "All-Rac-Alpha-Tocopherol" OR "Beta-Tocotrienol" OR "D-Alpha-Tocopherol" OR "D-Alpha-Tocopheryl" OR "DL-Alpha-Tocopherol" OR "DL-Alpha-Tocopheryl" OR "D-Tocopherol" OR "DL-Tocopherol" OR "D-Beta-Tocopherol" OR "D-Delta-Tocopherol" OR "Delta-Tocotrienol" OR "Delta-Tocopherol" OR "D-Gamma-Tocopherol" OR "D-Gamma-Tocotrienol" OR "DL-Tocopherol" OR "Gamma-Tocotrienol" OR "RRR-Alpha-Tocopherol" OR "Succinate Acide de Tocopheryl" OR "Tocopherol*" OR "Tocotrienol*" OR "Vitamina E" OR "Vitamine E" OR "Vitamine Liposoluble" OR "Vitamin K" OR "Phytonadione" OR "Menaquinone" OR "Menadione" OR "Menadiol" OR "4-amino-2-methyl-1-naphthol" OR "Vitamin K1" OR "Methylphytyl Naphthoquinone" OR "Phylloquinone" OR "Phytomenadione" OR "Phytonadione" OR "2-Methyl-3-Phytyl-1,4-Naphthoquinone" OR "Vitamina K" OR "Vitamine K" OR "Vitamine Liposoluble" OR "Vitamine Soluble dans les Graisses" OR "Vitamin K2" OR "Menatetrenone" OR "Vitamin K3" OR "2-Methyl-1,4-Naphthoquinone" OR "Vitamin K4" OR "Menadiolum Solubile Methylnaphthohydroquinone" OR "Vitamin K5" OR "Vitamins" OR "Agneau du Moine" OR "Agneau-chaste" OR "Agni Casti" OR "Agnocasto" OR "Agnolyt" OR "Agnus-Castus" OR "Chaste Berry" OR "Chaste Tree" OR "Chasteberry" OR "Chastetree" OR "Gattilier" OR "Hemp Tree" OR "Mang Jing Zi" OR "Panj-Angosht" OR "Pimiento del Monje" OR "Vitex" OR "Viticis Fructus" OR "Vojta Method" OR "Voodoo" OR "Water Aerobics" OR "Western Herbal Medicine" OR "Native American Herbs" OR "Western Herbalism" OR "Western Herbs" OR "White Willow" OR "Salix alba" OR "European Willow" OR "Whole Diet" OR "Whole Medical Systems" OR "Whole Systems" OR "Whole Practices" OR "Whole-Body Approach" OR "Whole-Body Vibration therap*" OR "Wigmore Diet" OR "Wild Medicine" OR "Avellano de Bruja" OR "Cafe du Diable" OR "Hamamelis" OR "Hazel" OR "Noisetier des Sorcieres" OR "Snapping Tobacco Wood" OR "Spotted Elder" OR "Winter Bloom" OR "Witchcraft therap*" OR "Occult therap*" OR "Xiaxingci Granule" OR "Yarrow" OR "Achilee" OR "Achillee" OR "Achillea" OR "Acuilee" OR "Band Man's Plaything" OR "Bauchweh" OR "Birangasifa" OR "Birangasipha" OR "Biranjasipha" OR "Bloodwort" OR "Bumadaran" OR "Civan Percemi" OR "Devil's Nettle" OR "Devil's Plaything" OR "Erba Da Cartentieri" OR "Erba Da Falegname" OR "Gandana" OR "Gemeine Schafgarbe" OR "Green Arrow" OR "Katzenkrat" OR "Little Feather" OR "Milefolio" OR "Milenrama" OR "Milfoil" OR "Millefeuille" OR "Millefolii Flos" OR "Millefolii Herba" OR "Millefolium" OR "Millegoglie" OR "Nosebleed" OR "Plumajillo" OR "Rajmari" OR "Roga Mari" OR "Sanguinary" OR "Sourcil de Venus" OR "Staunchweed" OR "Tausendaugbram" OR "Thousand-Leaf" OR "Wound Wort" OR "Yohimbe" OR "Corynanthe Johimbe" OR "Johimbi" OR "Yohimbehe" OR "Yohimbine" OR "Yohimbinum Muriaticum" OR "Zero Balancing" OR "Zhixian I Pill" OR "Zinc" OR "Zn" OR "Atomic number 30" OR "Numero Atomique 30" OR "Polaprezinc" OR "Zincum Aceticum" OR "Zincum Gluconicum" OR "Zincum Metallicum" OR "Zincum Valerianicum" OR "Zishen Tongli Jianonang")

Date Run: Sat Dec 21 2024 02:54:05 GMT+0800 (中国标准时间)

Results: 4154282

2: TI ＝ (“knee osteoarthritis” OR “Knee Osteoarthritides” OR “Knee Osteoarthritis” OR

“Osteoarthritis of the Knee” OR “Osteoarthritis of Knee” OR “Osteoarthritis, Knee” OR “KOA”) Date Run: Sat Dec 21 2024 02:59:26 GMT+0800 (中国标准时间) Results: 17240

3: #2 AND #1 Date Run: Sat Dec 21 2024 02:59:41 GMT+0800 (中国标准时间) Results: 2859

4: #2 AND #1 and Book Review or Data Paper or Note or Retraction or Early Access or Correction or Proceeding Paper or Retracted Publication or News Item or Editorial Material or Letter or Meeting Abstract (Exclude – Document Types) Date Run: Sat Dec 21 2024 03:02:06 GMT+0800 (中国标准时间) Results: 1834

5: #2 AND #1 and Book Review or Data Paper or Note or Retraction or Early Access or Correction or Proceeding Paper or Retracted Publication or News Item or Editorial Material or Letter or Meeting Abstract (Exclude – Document Types) and Japanese or Korean or Spanish or Portuguese or Italian or Turkish or Russian or German (Exclude – Languages) Date Run: Sat Dec 21 2024 03:04:00 GMT+0800 (中国标准时间) Results: 1815
